# Activity of Fluoro-Substituted 3,5-Di[(*E*)-Benzylidene]piperidin-4-Ones with *N*-Arylsulfonyl Appendages Against KRAS-Mutant Pancreatic Ductal Adenocarcinoma Cells

**DOI:** 10.3390/ijms272110013

**Published:** 2026-11-08

**Authors:** Hindole Ghosh, Duha Qamar Azaz, Addhyan Saxena, Nicole Bodenhausen, Rainer Schobert, Bernhard Biersack, Prasad Dandawate

**Affiliations:** 1 Cancer Biology, University of Kansas Medical Center, 3901 Rainbow Boulevard, Kansas City, KS 66160, USA; hghosh@kumc.edu (H.G.); duhaq.azaz@gmail.com (D.Q.A.); addhyansaxena@gmail.com (A.S.); nibo2020@icloud.com (N.B.); 2 Organic Chemistry 1, University of Bayreuth, Universitaetsstrasse 30, 95440 Bayreuth, Germany; rainer.schobert@uni-bayreuth.de

**Keywords:** curcumin, sulfonamide, anticancer agents, apoptosis, pancreatic ductal adenocarcinoma

## Abstract

KRAS-mutant pancreatic ductal adenocarcinoma (PDAC) is characterized by a high mortality that still lacks efficient therapies. Monoketone-based bisarylidene curcuminoid derivatives have shown promising activities against various PDAC models. Based on our previous findings on anticancer-active *N*-acryloyl-modified piperidin-4-one derivatives, we replaced the reactive acryloyl moiety by a series of *N*-arylsulfonyl bis-difluoro/trifluoromethylbenzylidene piperidin-4-one derivatives and tested them for antiproliferative activity against KRAS-mutant PDAC cells. Furthermore, apoptosis induction based on caspase-3/7 activation and STAT3 inhibition were investigated for the most promising compound. Notably, the *N*-tosyl derivative **3a** with 4-trifluoromethyl substituents at the benzylidene rings exhibited the highest antiproliferative activities against PDAC cells (IC_50_ = 0.22–0.26 µM, 48 h). Derivative **3a** also strongly induced apoptosis in PDAC cells and suppressed STAT3 activation/phosphorylation, which was supported by molecular docking of this curcuminoid into the SH2 domain of STAT3 and by **3a**-STAT3 binding confirmed using CETSA in MiaPaCa-2 cells. These findings identify compound **3a** as a promising lead for further preclinical evaluation in KRAS-mutant PDAC models and in other STAT3-dependent cancer types.

## 1. Introduction

The curative therapy of pancreatic cancer is still an unmet health issue, and thus pancreatic cancer remains one of the deadliest human tumor diseases. Clinically applied treatment options such as surgery, chemotherapy (e.g., gemcitabine, erlotinib, FOLFIRINOX), and radiotherapy can prolong patient survival but usually do not protect from tumor relapse and recurrence [1,2]. Pancreatic ductal adenocarcinoma (PDAC) is an especially perilous cancer type, and the development of new efficient drugs against PDAC has been complicated [3]. Notably, approximately 90% of all PDAC cases are KRAS-mutant, which has an impact on the overall survival of PDAC patients [4]. Thus, recent therapeutic advances include daraxonrasib, a multi-selective RAS inhibitor approved by the U.S. Food and Drug Administration, for certain adults with metastatic pancreatic adenocarcinoma after prior systemic therapy or for those who are not candidates for multi-agent systemic therapy [5].

**Figure 1:**
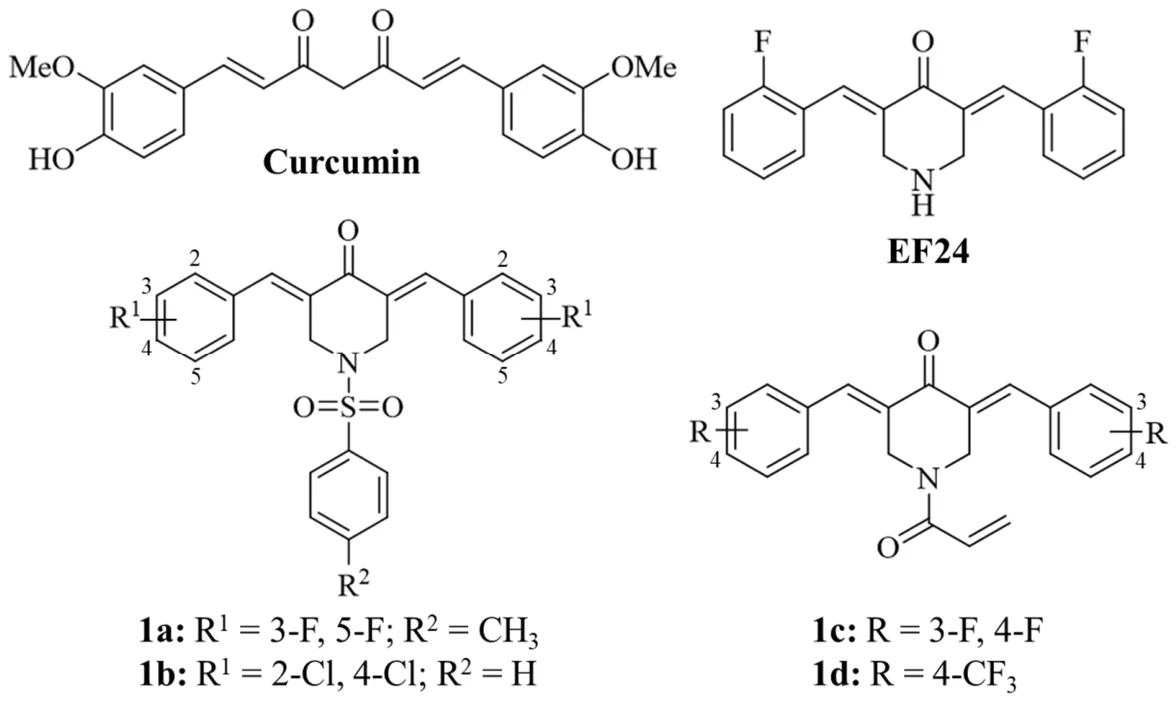
Structures of curcumin and of the anticancer active piperidin-4-one-based curcuminoids EF24, arylsulfonamides **1a** and **1b**, and *N*-acryloyl derivatives **1c** and **1d**.

However, along with the increasing relevance of RAS inhibitors to KRAS-mutant PDAC therapy, the problem of RAS inhibitor resistance is also rising [6]. The EGFR/HER2-4 inhibitor afatinib and the STAT3 suppressor SD36 were recommended as combination partners of daraxonrasib to prevent adaptive RAS inhibitor resistance in PDAC models [7]. In particular, natural STAT3 inhibitors have shown promising activities against cancers and associated inflammatory processes [8]. Curcumin (diferuloylmethane), a biologically active constituent of *Curcuma longa* root powder used as a spice in Indian cuisine, is a prominent example since it has shown both STAT3- and EGFR-suppressing effects in cancer (Figure 1) [9,10]. In addition, curcumin was shown to be a potent inhibitor of drug-resistant cancers and exhibited promising activities against PDACs, including suppression of HRAS, AKT1, and EGFR [11,12]. However, curcumin has poor bioavailability, limiting its clinical application as an anticancer drug [13].

To improve activity and pharmacological properties in cancer, researchers developed various monocarbonyl-based curcuminoids, including the most prominent example, the piperidin-4-one derivative EF24 (Figure 1) [14,15]. Meanwhile, numerous curcuminoid derivatives, such as modified curcumins and monoketone-based EF24-type curcuminoids, have been described as active compounds against PDAC [16]. Notably, a curcumin-sulfonamide hybrid was reported to be active against lung and gastric cancer cells [17]. In terms of *N*-sulfonyl-modified EF24 derivatives, the monoketone-based *N*-arylsulfonamides **1a** and **1b** were identified as having considerable anticancer activity against the NCI panel of 60 cancer cell lines (Figure 1) [18]. However, PDAC cell lines were not included in these tests. Notably, analogous aryl sulfonamides showed high activities against human pathogenic protozoal parasites such as *Leishmania major* and *Toxoplasma gondii* [19]. Various other sulfonamide derivatives have been reported as pro-apoptotic agents (Bcl-2 inhibitors) [20]. Moreover, anticancer-active sulfonamides have been described as inhibitors of relevant tumor targets such as protein kinases, carbonic anhydrases, matrix metalloproteinases, and histone deacetylases [21]. To target STAT3 in KRAS-mutant PDAC cells, our group recently designed and identified *N*-acryloyl derivatives of EF24, such as compounds **1c** and **1d**, with improved activity against PDAC (Figure 1) [22]. However, reactive *N*-acryloyl moieties can have detrimental pharmacological properties. Aryl sulfonyl fragments can be suitable, more stable alternatives to acryl derivatives and can lead to new promising drug candidates for PDAC treatment.

Since there are no anticancer data for the *N*-arylsulfonyl curcuminoids **1a** and **1b** from PDAC cells available yet, we decided to fill this gap, prepared a series of known and new *N*-arylsulfonamide-based monocarbonyl curcuminoids with difluoro- or trifluoromethylbenzylidene moieties, and tested them for anticancer activity against PDACs together with suitable control compounds.

## 2. Results

### 2.1. Curcuminoid Test Compounds

**Scheme 1:**
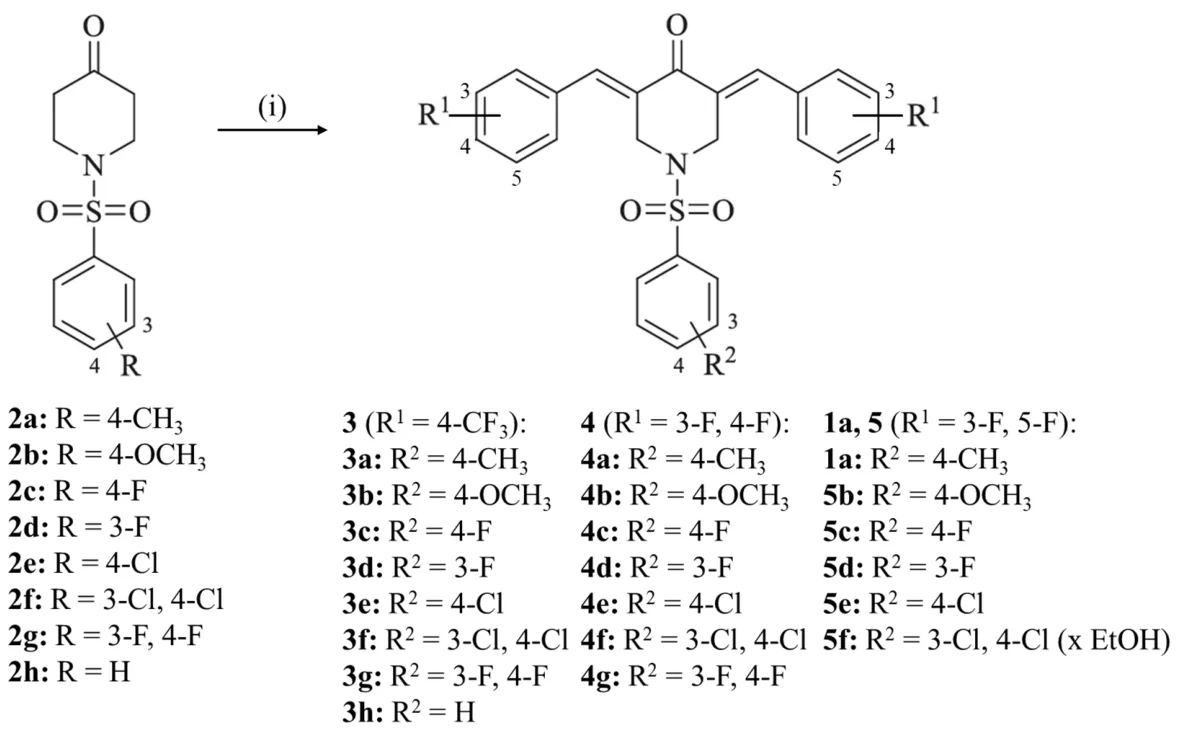
Reagents and conditions: (i) 4-trifluoromethylbenzaldehyde or 3,4-/3,5-difluorobenzaldehyde, conc. HCl, EtOH, reflux, 24 h, 30–61%.

The *N*-arylsulfonyl-modified bis-3,5-(4-trifluoromethylbenzylidene)-piperidin-4-ones **3a–h** were prepared from the condensation reaction of the *N*-aryl sulfonyl piperidin-4-ones **2a–h** with 4-trifluoromethylbenzaldehyde under acidic conditions in refluxing EtOH, which was described previously (Scheme 1) [18,19].

Analogously to compounds **3a–h**, the 3,4-difluorobenzylidenes **4a–g** and 3,5-difluorobenzylidenes **1a** and **5b–f** were obtained (Scheme 1). Among these compound series, only the *N*-tosyl derivatives **1a** and **4a** have already been published, whereas **4b–g** and **5b–f** are new sulfonamide-based curcuminoids (Scheme 1, Figure 1) [18]. The new sulfonamides were obtained as yellow solids in accordance with the color and aggregation state of the known sulfonamide analogs.

**Scheme 2:**
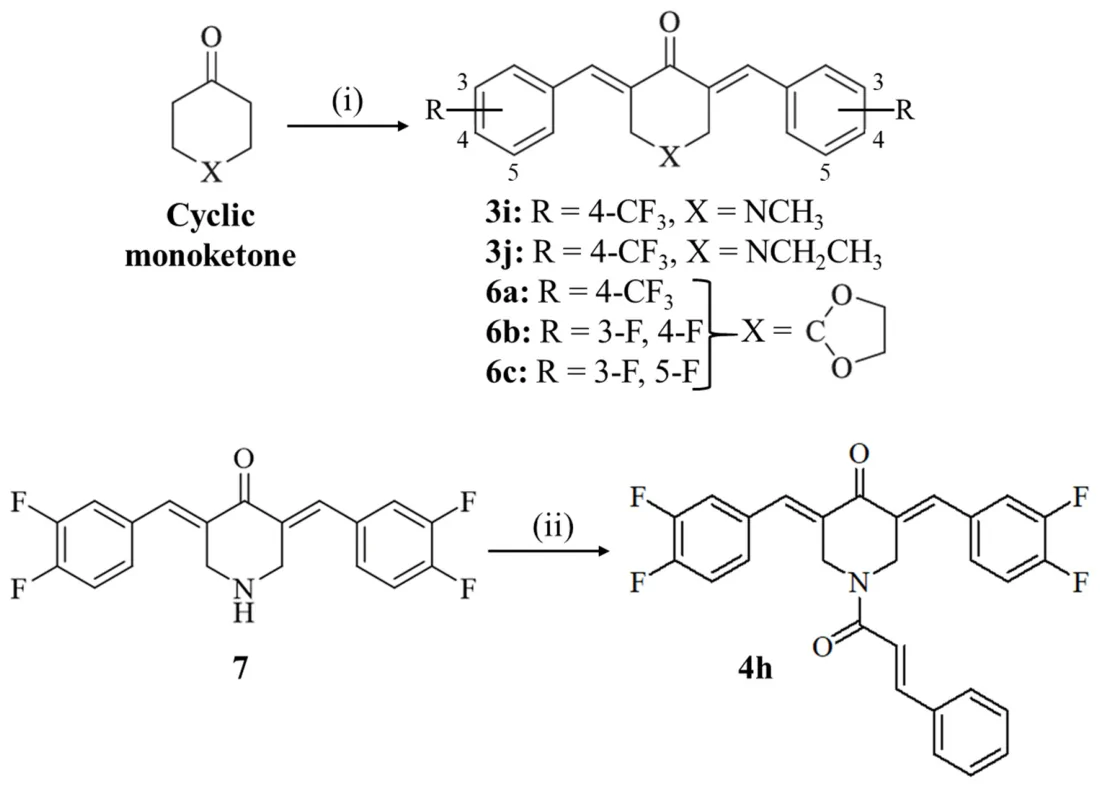
Reagents and conditions: (i) 4-trifluoromethylbenzaldehyde or 3,4-/3,5-difluorobenzaldehyde, aq. NaOH, MeOH, r.t., 1 h, 40–44%; (ii) cinnamoyl chloride, aq. K_2_CO_3_, acetone, r.t., 24 h, 75%.

The known *N*-(m)ethyl curcuminoids **3i** and **3j** were applied for comparison purposes and were prepared under basic conditions in MeOH at room temperature (Scheme 2) [19]. The monoethylene acetals **6a–c** were also prepared under basic conditions in MeOH at room temperature (Scheme 2). The ethylene acetal moiety has two oxygen atoms that can mimic, in theory, the sulfonyl oxygen atoms of the sulfonamides. Compound **6a** was published in a previous study, whereas **6b** and **6c** are new derivatives [19]. Moreover, a new cinnamide **4h** was prepared from the reaction of the known piperidin-4-one-based curcuminoid and EF24 analog **7** with cinnamoyl chloride for comparison with the sulfonamide analogs (Scheme 2) [19]. The new acetals **6b** and **6c**, as well as the cinnamide **4h**, were also obtained as yellow solids. NMR spectra of the target compounds are provided in the Supplementary Materials (Figures S1–S42).

### 2.2. Anticancer Activity

#### 2.2.1. Antiproliferative Activity

The curcuminoids described in Section 2.1. were tested for their antiproliferative activities against two KRAS-mutant PDAC cell lines, MiaPaCa-2 and S2-007 (Table 1). The *N*-tosyl derivative **3a** of the 4-trifluoromethylphenyl series was the most active compound with IC_50_ values of 0.22–0.26 µM in both PDAC cell lines. Compound **3a** was more active than the *N*-alkyl analogs (**3i** and **3j**) and the approved anticancer drug 5-fluorouracil (5-FU, a component of the FOLFIRINOX regimen). The replacement of the methyl group of the tosyl fragment by a chloro substituent (**3e**) led to slightly lower activities (IC_50_ = 0.47–0.55 µM), while analogs with fluoro (**3c**) and methoxy (**3b**) substituents, as well as with 3,4-difluoro (**3g**) and 3,4-dichloro (**3f**) substituents, were distinctly less active. The besylate **3h** was also less active, underscoring the importance of the aryl sulfonyl substituent for activity against PDAC cells. Only the 3-fluorophenylsulfonamide **3d** showed IC_50_ values below 1 µM and was thus more active than its 4-fluorophenyl congener **3c**. Furthermore, **3b** and **3h** showed activity only against S2-007 cells (IC_50_ = 1.18 µM and 3.03 µM, respectively) but were inactive against MiaPaCa-2 cells (IC_50_ >10 µM).

Notably, the tosyl fragment was also present in the most active compounds **1a** (IC_50_ = 0.67–0.70 µM) and **4a** (IC_50_ = 0.55–0.83 µM) in the 3,5-difluorophenyl and 3,4-difluorophenyl curcuminoid series, respectively, and these were only slightly less active than **3a** (Table 1). The 3,4-difluorophenyl analogs **4b**, **4e**, and **4f** exhibited virtually the same or slightly better activities than **4a** against both PDAC cell lines (IC_50_ = 0.44–0.73 µM). Compounds **4c**, **4d**, and **4g** showed slightly lower activity, with IC_50_ values still below or around 1 µM. Cinnamide **4h** was only slightly less active than the sulfonamide analogs.

Among the 3,5-difluorophenyl compounds, only tosylate **1a** was active against PDAC cell lines at concentrations below 1 µM (Table 1). Derivative **5e** reached the activity of **1a** only against S2-007 cells. The other compounds were distinctly less active, with **5d** being the least active analog.

Notably, the monoethylene acetals **6a–c** were much less active than the sulfonamide derivatives (Table 1). All three compounds were inactive against MiaPaCa-2 cells (IC_50_ > 10 µM), whereas only the 4-trifluoromethylphenyl analog **6a** exhibited moderate activity against S2-007 cells (IC_50_ = 4.68 µM).

**Figure 2:**
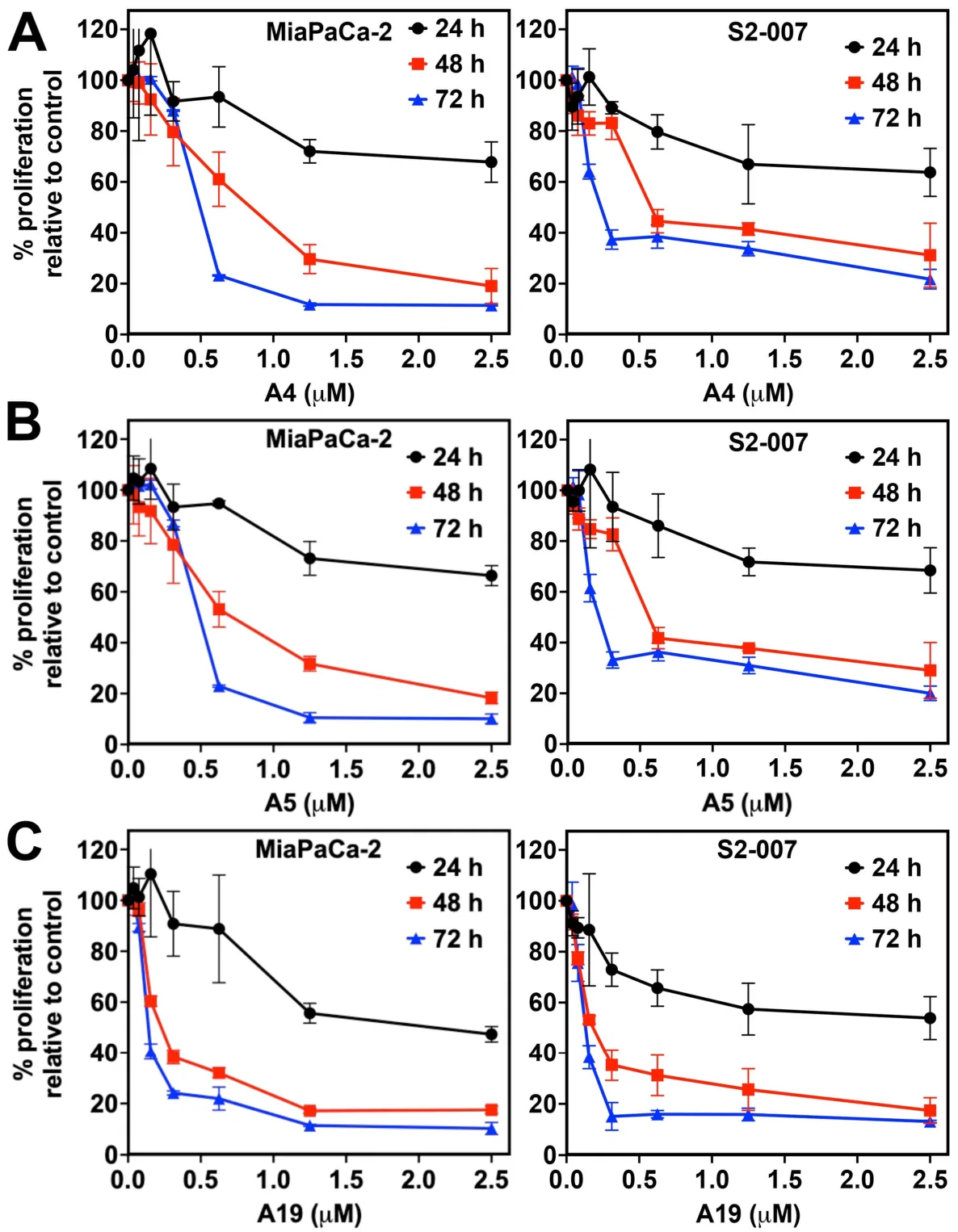
Dose- and time-dependent antiproliferative activity of **4b** (A4, panel (**A**)), **4a** (A5, panel (**B**)), and **3a** (A19, panel (**C**)) against MiaPaCa-2 and S2-007 PDAC cells. Cells were treated with 0–2.5 µM of each compound for 24–72 h, and proliferation was measured by hexosaminidase assay and expressed as a percentage of untreated control. Data are presented as mean ± SD of three independent experiments.

We also investigated the time- and dose-dependent antiproliferative activities of **3a** (A19), **4a** (A5), and **4b** (A4) against MiaPaCa-2 and S2-007 cells (Figure 2). All three curcuminoids showed their highest activities after 72 h (IC_50_ = 0.13–0.14 µM for **3a**, 0.21–0.49 µM for **4a**, 0.24–0.49 µM for **4b**). In addition, considerable antiproliferative activity (approximately 60% proliferation in comparison with control) of these curcuminoids was already achieved after 24 h at higher concentrations (between 1 and 2.5 µM). But only **3a** exhibited a 24 h–IC_50_ value below 2.5 µM (2.09 µM) in the MiaPaCa-2 cells, indicating a particularly early sensitivity of these PDAC cells to this curcuminoid.

**Figure 3:**
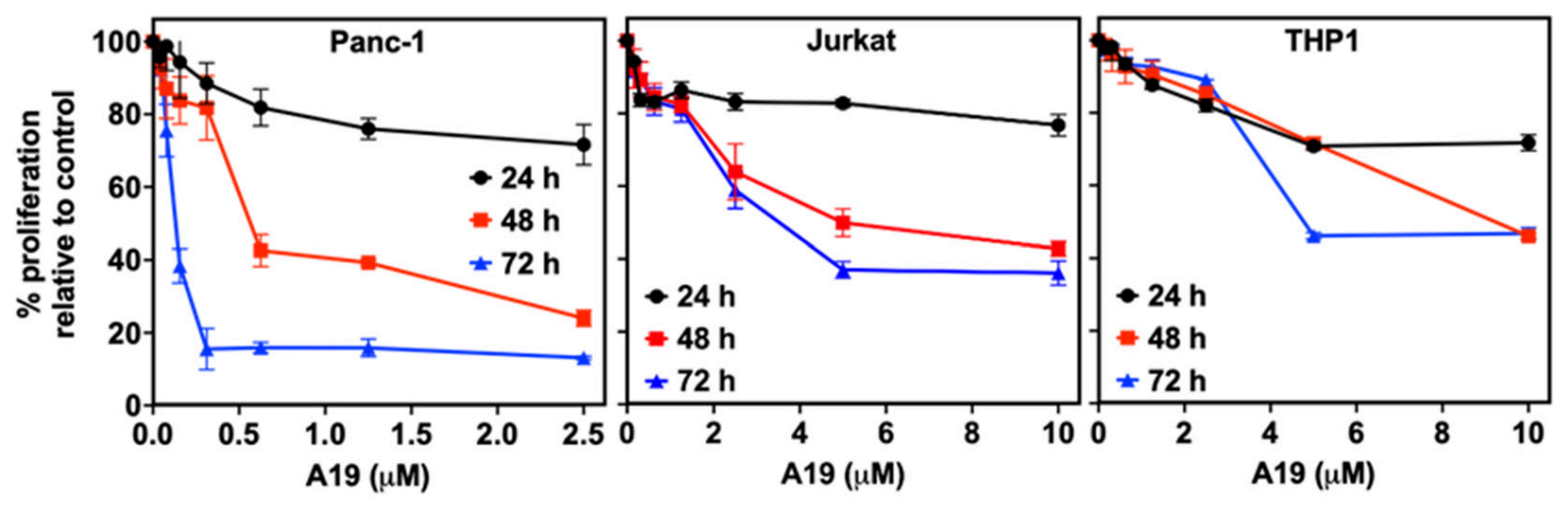
Dose- and time-dependent antiproliferative activity of **3a** (A19) against Panc-1 PDAC cells (0–2.5 µM), Jurkat, and THP-1 cells (0–10 µM) after 24–72 h of compound treatment. Proliferation was measured by hexosaminidase assay (Panc-1) or CCK-8 assay (Jurkat and THP-1) and expressed as a percentage of untreated control. Data are presented as mean ± SD of three independent experiments.

To assess the selectivity, compound **3a** was also tested against Panc-1 PDAC cells and two human hematopoietic cell lines, Jurkat (an immortalized line of human T lymphocyte cells) and THP-1 monocytes (Figure 3). The results were compared with its activities against MiaPaCa-2 and S2-007 cells (Table 2). At shorter incubation times (24 h and 48 h), **3a** was somewhat less effective against Panc-1 cells than against MiaPaCa-2 and S2-007 cells. Its 48 h IC_50_ value in Panc-1 cells (0.56 µM) was about twice that obtained in MiaPaCa-2 and S2-007 cells (0.22–0.26 µM). After 72 h, however, **3a** inhibited Panc-1 proliferation as potently as in the other two PDAC cell lines, with an identical IC_50_ value of 0.13 µM (Figure 3).

In contrast, **3a** was distinctly less active against Jurkat and THP-1 cells, which required considerably higher concentrations (up to 10 µM) for growth inhibition (Figure 3). Neither cell line reached 50% inhibition after 24 h (IC_50_ > 10 µM). After 48 h and 72 h, the IC_50_ values were 5.59 µM and 3.44 µM for Jurkat cells and 9.23 µM and 4.75 µM for THP-1 cells, respectively (Table 2). Based on the 72 h IC_50_ values, **3a** was approximately 25- to 26-fold more active against the three PDAC cell lines than against Jurkat cells and approximately 34- to 37-fold more active than against THP-1 cells (Supplementary Table S1). This indicates a considerable preference for PDAC cells after 72 h incubation across all three PDAC cell lines.

#### 2.2.2. Apoptosis Induction

**Figure 4:**
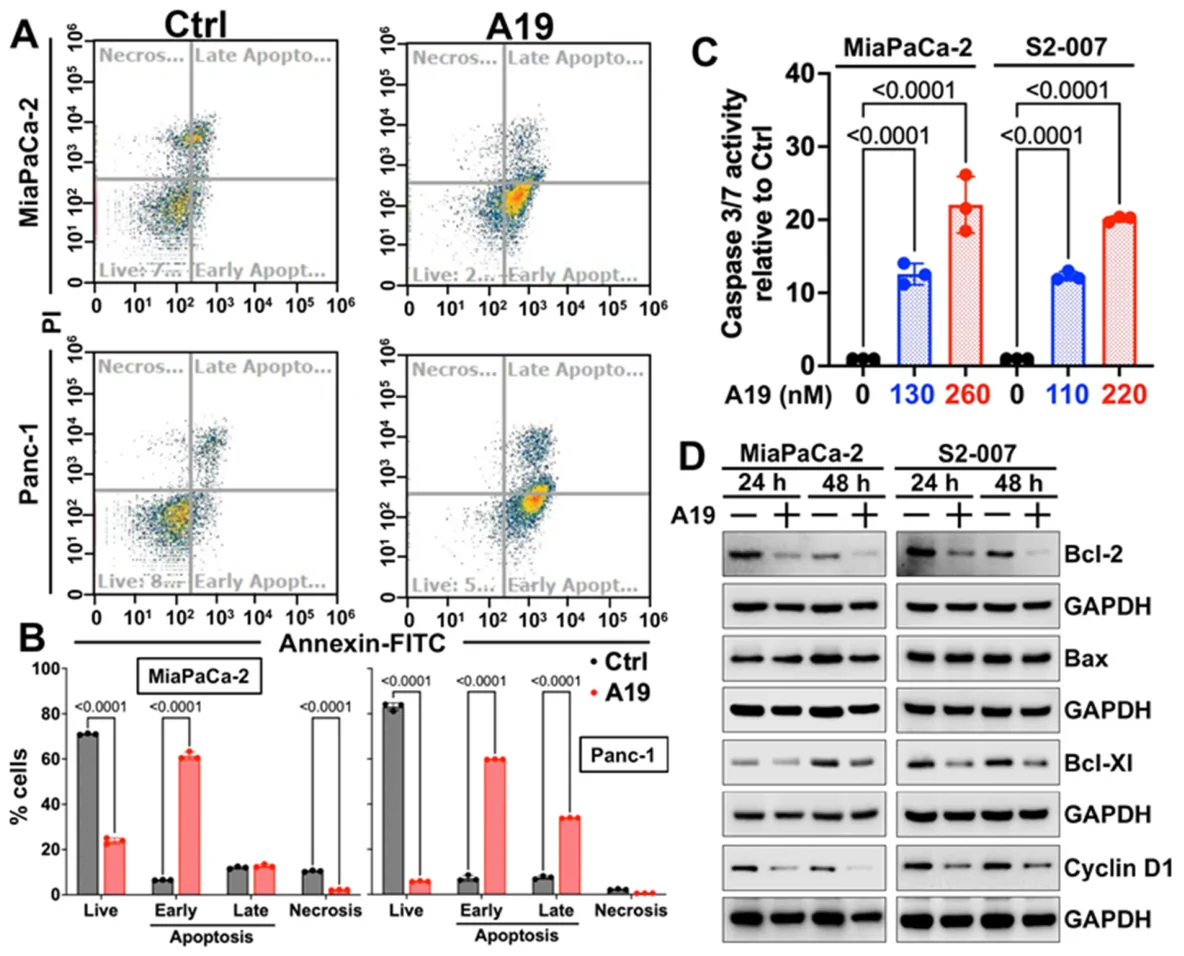
Compound **3a** induces apoptosis in PDAC cells. (**A**) Representative Annexin V-FITC/PI flow cytometry dot plots of MiaPaCa-2 (top) and Panc-1 (bottom) cells treated with vehicle (Ctrl) or **3a** (A19) at IC_50_ (260 nM MiaPaCa-2; 560 nM Panc-1) for 48 h. Quadrants show live (Annexin V^−^/PI^−^), early apoptotic (Annexin V^+^/PI^−^), late apoptotic (Annexin V^+^/PI^+^), and necrotic (Annexin V^−^/PI^+^) cells. (**B**) Quantification of the percentage of cells in each quadrant for MiaPaCa-2 (left) and Panc-1 (right) cells. Dots represent independent experiments (*n* = 3); *p*-values were determined by an unpaired two-tailed Student *t*-test for each quadrant. (**C**) Caspase-3/7 activity in MiaPaCa-2 and S2-007 cells after 48 h of treatment with **3a** (A19) at ½ IC_50_ (130 nM MiaPaCa-2; 110 nM S2-007) or IC_50_ (260 nM MiaPaCa-2; 220 nM S2-007), expressed relative to untreated control (Ctrl, 0 nM). Data are presented as mean ± SD; dots represent independent experiments (*n* = 3). *p*-values were determined by one-way ANOVA with Bonferroni–Šídák correction for multiple comparisons versus control. (**D**) Effect of **3a** (A19) on apoptosis-related protein expression in MiaPaCa-2 and S2-007 cells. Western blots show reduced Bcl-2, Bcl-Xl, and cyclin D1 levels in cells treated with **3a** at its IC_50_ concentration (+) for 24 h or 48 h compared with vehicle (−), while Bax expression remained unchanged. GAPDH was used as a loading control. Densitometric quantification from three independent experiments is shown in Figure S43.

To directly confirm apoptosis induction by **3a** (A19), apoptosis was quantified by Annexin V-FITC/PI flow cytometry in MiaPaCa-2 and Panc-1 cells following treatment with **3a** at its IC_50_ concentration for 48 h (Figure 4A,B). Treatment with **3a** markedly reduced the live cell population and increased both early and late apoptotic fractions in both cell lines relative to vehicle-treated controls. In MiaPaCa-2 cells, the combined early and late apoptotic population increased from 18.5% to 74.1% (*p* < 0.0001). An even more pronounced shift was observed in Panc-1 cells, where the combined apoptotic fraction rose from 14.7% to 93.7% (*p* < 0.0001). Necrotic cell fractions remained low and were not increased by **3a** treatment in either cell line, consistent with an apoptotic rather than necrotic mechanism of cell death.

The ability of **3a** to induce apoptosis via caspase-3/7 activation was further assessed in MiaPaCa-2 and S2-007 cells (Figure 4C). Cells were treated for 48 h at the ½IC_50_ (130 nM MiaPaCa-2; 110 nM S2-007) and IC_50_ (260 nM MiaPaCa-2; 220 nM S2-007) concentrations of **3a**, and caspase-3/7 activity was measured relative to untreated controls. At ½IC_50_, **3a** produced a 12- to 13-fold increase in caspase-3/7 activity in both cell lines relative to untreated controls (*p* < 0.0001). At the IC_50_, this increased further to 20- to 22-fold, indicating clear dose-dependent induction of apoptosis by **3a** in both PDAC lines. The pro-apoptotic effects of **3a** (A19) on MiaPaCa-2 and S2-007 cells were further examined by monitoring the expression of pro- and anti-apoptotic proteins, including Bcl-2, Bcl-Xl, and Bax (Figure 4D and Figure S43). Treatment with **3a** (A19) at its IC_50_ concentration markedly suppressed the anti-apoptotic proteins Bcl-2 and Bcl-Xl in both cell lines at 24 h and 48 h. The expression of pro-apoptotic Bax remained unaffected by **3a** (A19) at either time point in either cell line. Cyclin D1 was also suppressed by **3a** in both cell lines at both time points.

#### 2.2.3. PDAC Spheroid Growth and Colony Formation

**Figure 5:**
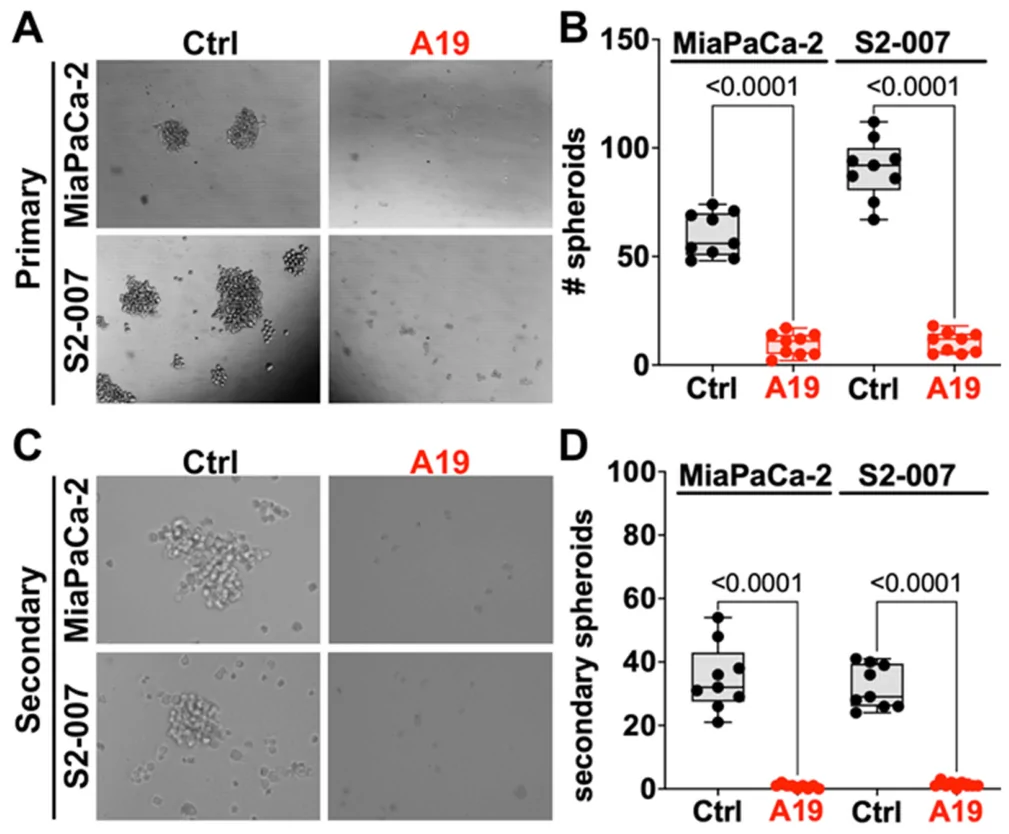
Compound **3a** inhibits primary and secondary spheroid formation in PDAC cells. (**A**) Representative brightfield images of primary MiaPaCa-2 (top) and S2-007 (bottom) spheroids after 7 days in the absence (Ctrl) or presence of **3a** (A19) at its IC_50_ concentration. (**B**) Quantification of primary spheroid number per well for MiaPaCa-2 and S2-007 cells. (**C**) Representative brightfield images of secondary MiaPaCa-2 (top) and S2-007 (bottom) spheroids formed after primary spheroids from control and **3a**-treated wells were dissociated and re-plated in drug-free spheroid medium for 5 days. (**D**) Quantification of secondary spheroid number per well for MiaPaCa-2 and S2-007 cells. Data in (**B**) and (**D**) are shown as box-and-whisker plots (median, interquartile range, and min–max) with individual data points; *p*-values were determined by unpaired two-tailed Student’s *t*-test. Dots represent individual wells (*n* = 9 per group, 3 wells from each of 3 independent experiments).

The growth of PDAC spheroids derived from MiaPaCa-2 and S2-007 cells was investigated in the absence and presence of **3a** (Figure 5). Indeed, **3a** treatment at its IC_50_ concentration produced a strong, significant inhibition of primary spheroid formation in both cell lines (*p* < 0.0001) (Figure 5A,B). To assess whether **3a** also affects the self-renewal capacity of spheroid-forming cells, primary spheroids were dissociated and re-plated in drug-free medium to form secondary spheroids (Figure 5C,D). Cells derived from control spheroids readily formed secondary spheroids. In contrast, cells derived from **3a**-treated spheroids almost completely failed to form secondary spheroids, with counts reduced in both cell lines (*p* < 0.0001). Secondary spheroid formation remained suppressed after drug removal, indicating that **3a** impairs the self-renewal of PDAC cells with stem-like properties.

**Figure 6:**
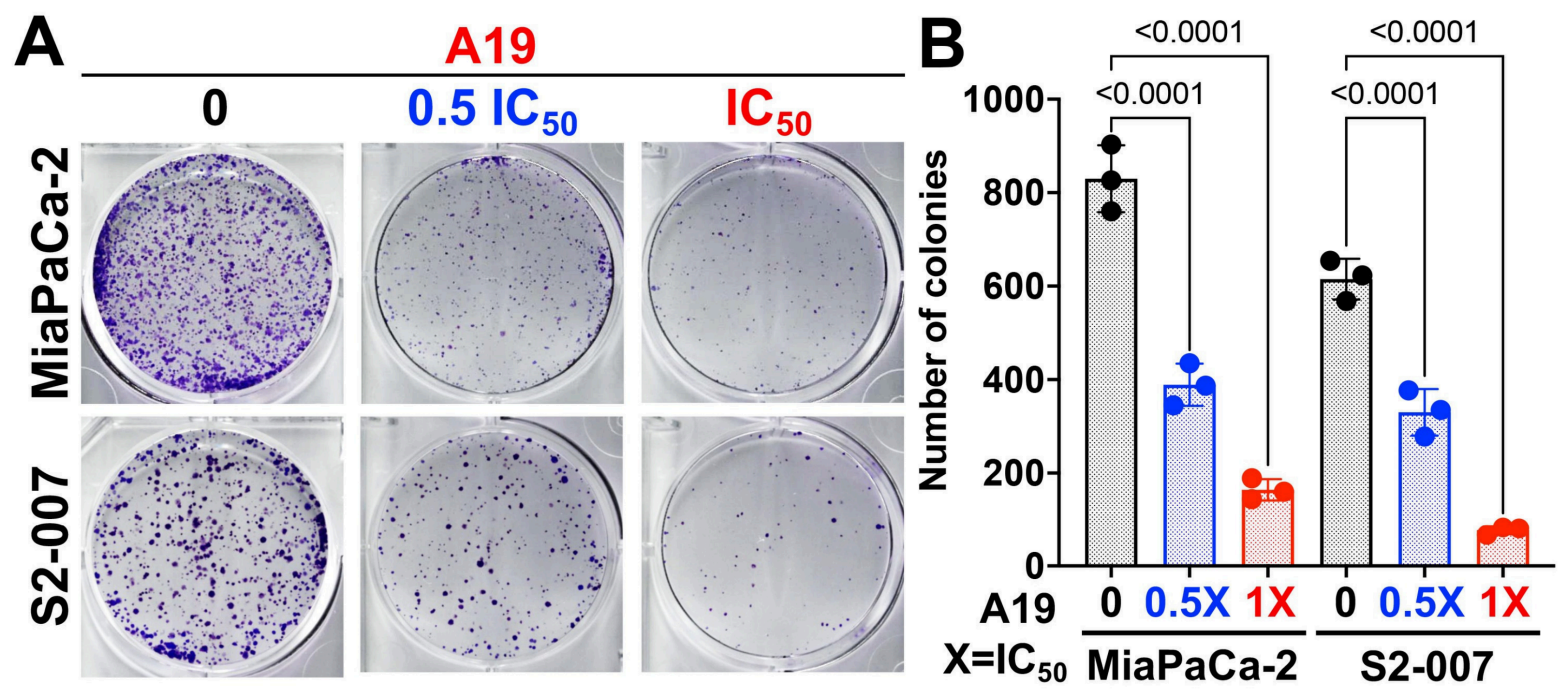
Compound **3a** inhibited colony formation in PDAC cells. (**A**) Representative crystal-violet-stained wells of MiaPaCa-2 (top) and S2-007 (bottom) cells 10–12 days after a 48 h treatment with 0, ½ IC_50_ (0.5X), or IC_50_ (1X) concentrations of **3a** (A19) followed by drug-free recovery. (**B**) Quantification of colony number for MiaPaCa-2 and S2-007 cells under the same conditions. Data are presented as mean ± SD; dots represent independent experiments (*n* = 3). *p*-values were determined by one-way ANOVA with Bonferroni–Šídák correction for multiple comparisons versus untreated control.

Moreover, the inhibition of colony formation by **3a** was evaluated in MiaPaCa-2 and S2-007 cells (Figure 6A,B). At its ½IC_50_ concentration, **3a** reduced colony formation by approximately 50% in both cell lines compared with untreated cells. At the IC_50_ concentration, colony formation was strongly abolished in MiaPaCa-2 and S2-007 cells.

#### 2.2.4. STAT3 Inhibition

**Figure 7:**
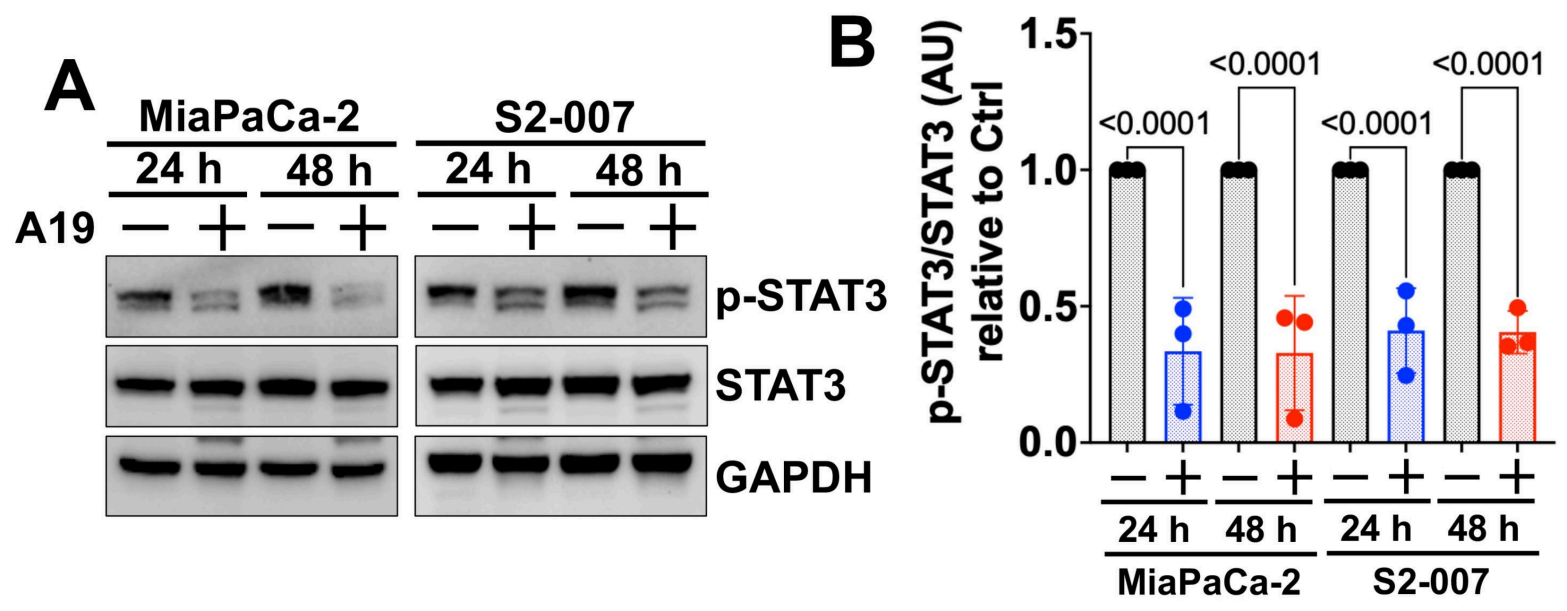
Compound **3a** inhibited STAT3 phosphorylation (Tyr705) in PDAC cells. (**A**) Western blots of p-STAT3 in MiaPaCa-2 and S2-007 cells treated with vehicle or **3a** (A19) at its IC_50_ concentration for 24 h or 48 h; GAPDH served as a loading control. (**B**) Densitometric quantification of p-STAT3 (Tyr705) normalized to total STAT3, relative to the respective untreated control, for each cell line and time point. Data are presented as mean ± SD; dots represent independent experiments (*n* = 3). *p*-values were determined by an unpaired two-tailed Student *t*-test versus the time-matched vehicle control.

The inhibition of STAT3 phosphorylation (Tyr705) by **3a** in PDAC cells (MiaPaCa-2 and S2-007) was investigated by Western blot (Figure 7A) and quantified relative to total STAT3 (Figure 7B). While **3a** did not affect total STAT3 expression, it markedly reduced levels of activated/phosphorylated p-STAT3 in both cell lines as early as 24 h, with suppression sustained through 48 h (*p* < 0.0001 at both time points in both cell lines).

**Figure 8:**
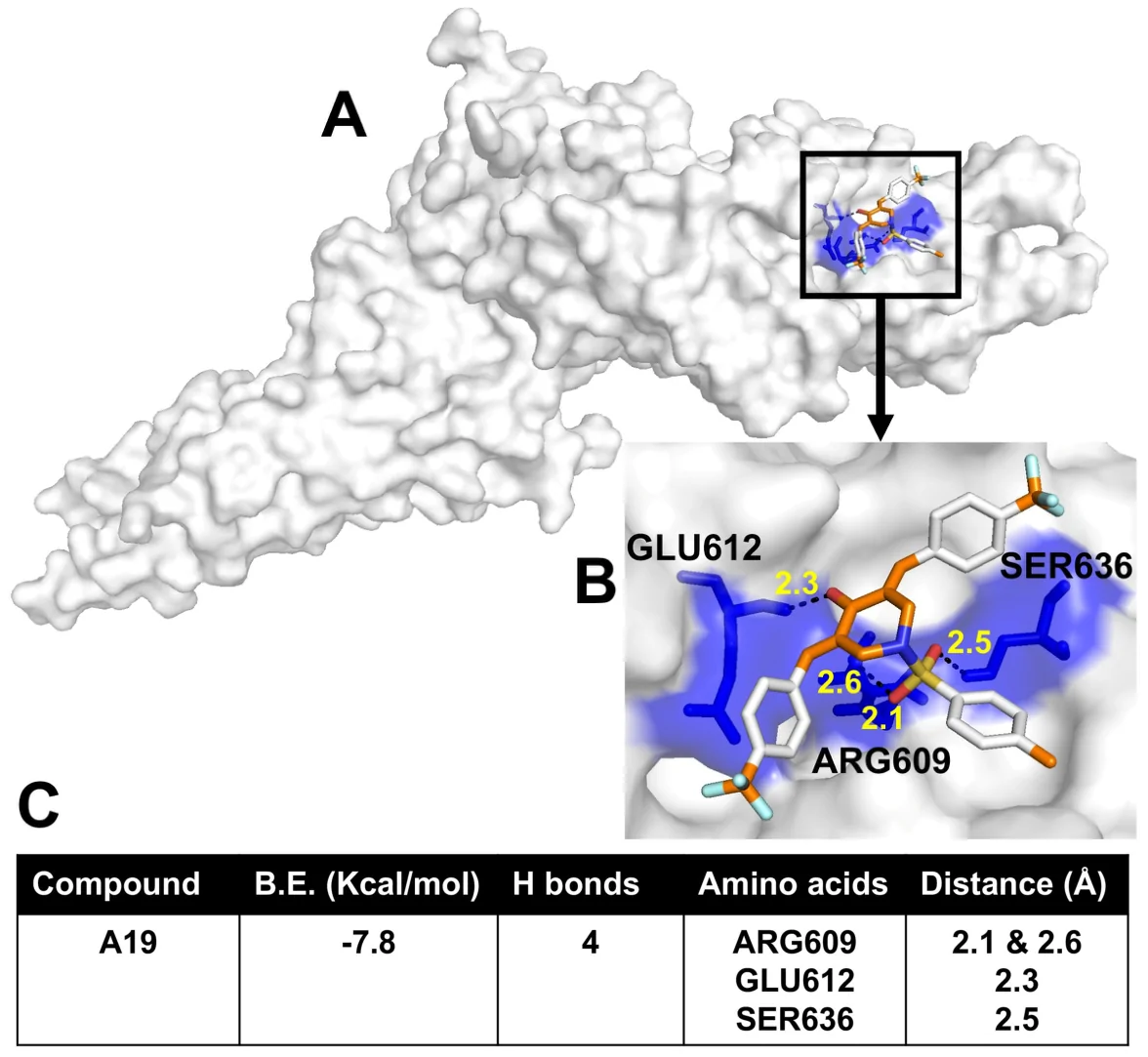
Docking of **3a** into the SH2 domain of STAT3. (**A**) Overview of the STAT3 surface (PDB ID: 6NJS; grey-white), with the predicted binding site of **3a** boxed. (**B**) Close-up of the boxed region. **3a** is shown as sticks with carbon atoms in orange (aromatic rings in white), oxygen in red, nitrogen in blue, sulfur in yellow and fluorine in cyan. Binding-site residues are shown as blue sticks, and the surface they contribute to is shaded blue; the three residues that hydrogen-bond to **3a** are labeled. Hydrogen bonds are drawn as black dashed lines, with the corresponding donor–acceptor distances given in Å (yellow). (**C**) Summary of the docking result for **3a** (A19): binding energy, number of hydrogen bonds, interacting residues, and hydrogen-bond distances.

The inhibition of STAT3 phosphorylation by **3a** suggests an interaction with the protein. A possible binding of **3a** to STAT3 was evaluated by molecular docking using a suitable STAT3 structure (PDB ID: 6NJS). Molecular docking of **3a** into the STAT3 SH2 domain revealed a considerable binding energy (B.E. = −7.8 kcal/mol). The formation of four H-bonds with the STAT3 amino acids ARG609 (two H-bonds via a sulfonyl oxygen of **3a**; 2.1 and 2.6 Å), GLU612 (one H-bond via the piperidin-4-one carbonyl oxygen of **3a**; 2.3 Å), and SER636 (one H-bond via a sulfonyl oxygen of **3a**; 2.5 Å) was determined, indicating a prominent role of the central *N*-sulfonyl-4-piperidone moiety in the interaction with the SH2 domain (Figure 8A–C).

**Figure 9:**
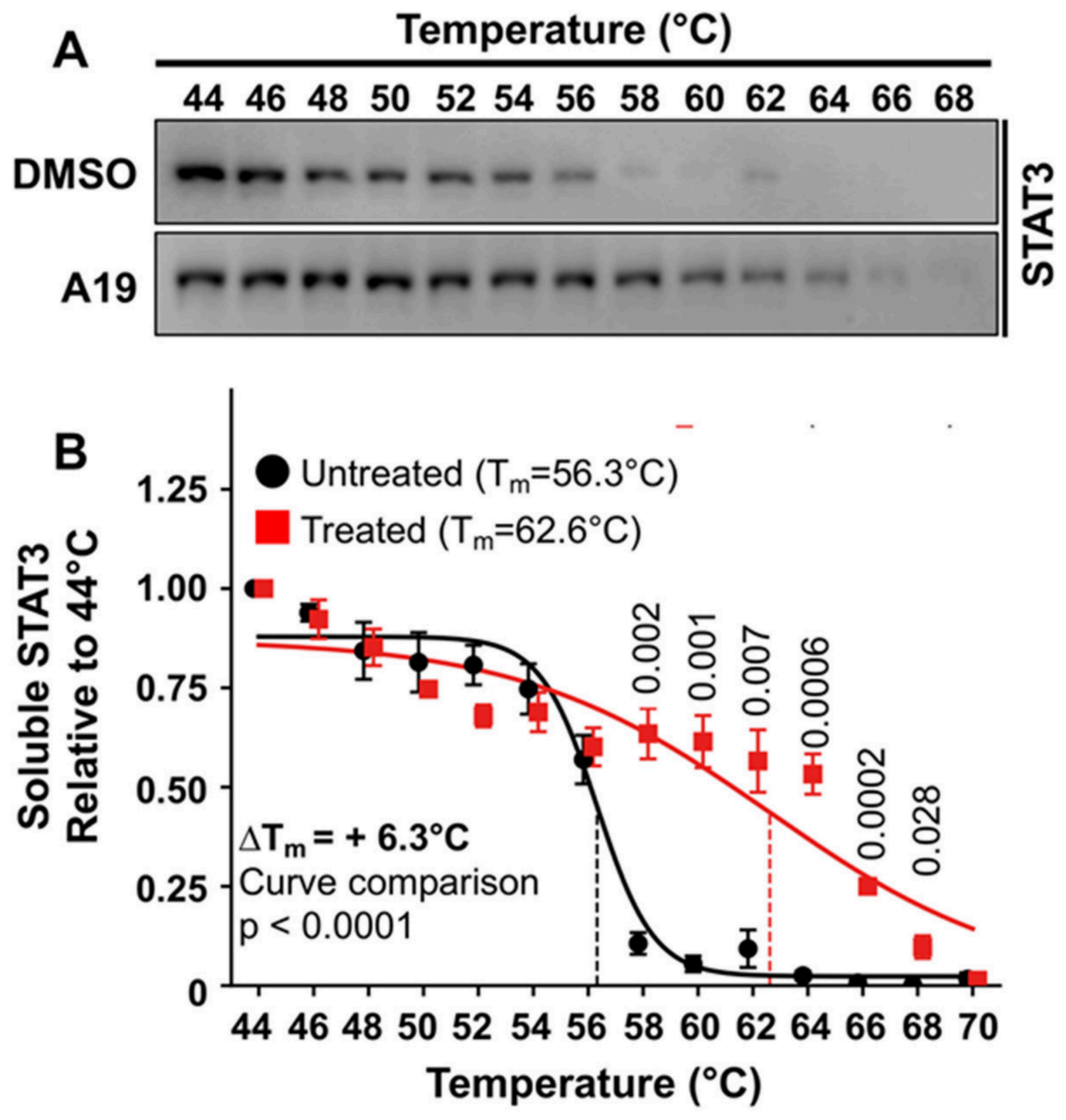
Cellular thermal shift assay (CETSA) showing that **3a** binds STAT3. (**A**) Representative Western blots of soluble STAT3 in MiaPaCa-2 cell lysates incubated with vehicle (DMSO) or **3a** (A19) at 20 µM for 4 h, then heated at the indicated temperatures (44–68 °C) for 3 min. (**B**) Melting curves of soluble STAT3, quantified by densitometry and normalized to the 44 °C sample of each group. Curves were fitted by nonlinear (Boltzmann sigmoidal) regression. Derivative **3a** raised the melting temperature (T_m_) of STAT3 from 56.3 °C (vehicle) to 62.6 °C (ΔT_m_ = +6.3 °C), indicating thermal stabilization of STAT3 by direct ligand binding. Data are presented as mean ± SD (*n* = 3). *p*-values above individual temperatures compare vehicle with **3a** at each temperature; the overall curve comparison (extra sum-of-squares F-test) gave *p* < 0.0001.

To confirm direct engagement of STAT3 by **3a**, a cellular thermal shift assay (CETSA) was performed in MiaPaCa-2 cell lysates (Figure 9). In vehicle-treated cell lysates, soluble STAT3 levels declined sharply above 54 °C, and the protein was largely precipitated at 58 °C and higher (Figure 9A). In cell lysates treated with **3a** at 20 µM for 4 h, soluble STAT3 remained detectable up to 64–66 °C. Quantification of the melting curves showed that **3a** shifted the melting temperature (Tm) of STAT3 from 56.3 °C to 62.6 °C (ΔTm = +6.3 °C; curve comparison *p* < 0.0001) (Figure 9B). Soluble STAT3 levels were significantly higher in **3a**-treated cell lysates at all temperatures between 58 °C and 68 °C (*p*-value = 0.0002–0.028). This thermal stabilization of STAT3 supports a direct interaction of **3a** with STAT3 in PDAC cells, consistent with the binding mode predicted by molecular docking.

## 3. Discussion

PDAC is a highly aggressive and lethal form of cancer with limited treatment options [3]. Research efforts to develop new potent drugs against PDAC include therapies targeting RAS, p53, CDKN2A mutants, NRG1 fusions, and TGF-β, as well as immune and CAR-T-cell therapies [23]. In this study, several *N*-aryl sulfonyl-modified monoketone curcuminoids were identified with high antiproliferative activity (i.e., nanomolar IC_50_ values) against PDAC cells. Except for a few examples (**3b**, **3h**, **5d**), the *N*-aryl sulfonamides were generally superior to the acetal analogs **6a–c**. The *N*-tosyl derivative **3a**, which is substituted with *para*-CF_3_ groups at both benzylidene rings, turned out to be the most active compound with a reasonable selectivity for PDAC cells. It was equally potent at nanomolar concentrations against all three PDAC cell lines in a dose- and time-dependent manner and was about 26- to 37-fold less active against the Jurkat and THP-1 cell lines. It should be noted, however, that Jurkat and THP-1 are leukemia-derived cell lines. These data therefore indicate preferential activity towards PDAC cells relative to cells of a different lineage, rather than selectivity over non-malignant cells. Evaluation in non-transformed pancreatic cells, such as hTERT-HPNE or HPDE, and in normal fibroblasts will be necessary to establish a therapeutic window for **3a**. The CF_3_ group is frequently applied in drug design and development because of its beneficial pharmacological properties [24]. However, in the present study, the CF_3_ group also conveyed higher antiproliferative activity in the *N*-tosylate **3a** in comparison with the analogous 3,4- and 3,5-difluorobenzylidene compounds **1a** and **4a**. Nevertheless, the tosyl group likely plays an important role in anticancer activity since *N*-tosylates **1a** and **4a** were also the best (3,5-difluoro analogs) or among the best sulfonamides (3,4-difluoro analogs) of the two difluorophenyl curcuminoid series. Thus, the tosylates **1a**, **3a**, and **4a** add well to the current arsenal of sulfonamide-based anticancer drugs and drug candidates [20,21]. Moreover, their activities against PDAC cells fill the gap left by a study from 2014, which reported the anticancer activity of **1a** against the NCI 60 cell line panel excluding PDAC cells [18]. The antiproliferative activities of **3a** were also in the activity range of the *N*-acryl derivatives **1c** and **1d** against MiaPaCa-2 and Panc-1 cells, while **3a** was distinctly more active than EF24 and definitely superior to the *N*-(m)ethyl analogs **3i** and **3j** [22]. Thus, the tosyl moiety is a promising *N*-attachment for 4-piperidone-based curcuminoids and a suitable surrogate for the *N*-acryl moiety of **1c** and **1d**.

Taken together, further experimental results support **3a** as a promising anticancer agent against PDAC. Notably, the compound inhibited PDAC spheroid growth and colony formation, which indicates that **3a** can suppress clonogenic survival and tumor sphere formation. Moreover, cells derived from **3a**-treated spheroids almost completely lost their ability to form secondary spheroids, even after replating them in drug-free medium. Secondary sphere formation is a measure of self-renewal, a defining property of cancer stem cells. Confirmation with established PDAC stem cell markers, such as CD44, CD133 and ALDH1, will be required to define the effect of **3a** on the cancer stem cell compartment more precisely. These data therefore suggest that **3a** does not merely delay sphere growth but durably impairs the stem-like cell population that drives tumor relapse and drug resistance in PDAC. These effects are in line with reports that curcumin and its derivatives can target cancer stem cells and suppress or prevent tumor growth and regrowth in various cancers, including PDAC [16,25]. Annexin V/PI flow cytometry and activation of caspases 3 and 7 required for apoptosis induction underscored the excellent cell-killing properties of **3a** without causing necrosis. This shows that **3a** kills PDAC cells mainly by apoptosis rather than by nonspecific necrotic toxicity. Consistent with these findings, **3a** downregulated the anti-apoptotic proteins Bcl-2 and Bcl-Xl in PDAC cells. The inhibition of apoptosis, or programmed cell death, is a hallmark of cancer; therefore, potent apoptosis inducers such as **3a** are needed as new effective anticancer agents [26]. The development and clinical approval of the Bcl-2 inhibitor venetoclax underscored the importance of apoptosis in cancer therapy; however, resistance limited its application [27]. Curcumin was reported to downregulate cyclin D1 [28]. Cyclin D1 can promote cell cycle progression in pancreatic cancer cells and maintain levels of the anti-apoptotic proteins Bcl-2 and Bcl-Xl [29]. Notably, **3a** also suppressed cyclin D1 expression. Since cyclin D1 has been reported to maintain the levels of the anti-apoptotic proteins Bcl-2 and Bcl-Xl in pancreatic cancer cells [29], its downregulation may contribute to the pro-apoptotic activity of **3a**, rather than reflecting cell-cycle arrest.

In addition to these promising cell death-associated mechanisms, **3a** also suppressed STAT3 activation in PDAC cells. Docking of **3a** into the SH2 domain (a protein–protein interaction domain required for STAT3 dimerization) revealed a considerable binding energy and the formation of four H-bonds between the *N*-sulfonyl-4-piperidone core fragment and crucial STAT3 amino acids. This predicted interaction was supported experimentally by CETSA. Derivative **3a** increased the thermal stability of STAT3 in MiaPaCa-2 cell lysates by 6.3 °C, a shift that indicates direct binding of the compound to the protein [30,31]. Together, docking and CETSA suggest that the reduced STAT3 phosphorylation in **3a**-treated PDAC cells results, at least in part, from direct engagement of STAT3 rather than only from effects on upstream kinases. Future studies should directly confirm binding of **3a** to recombinant STAT3 by surface plasmon resonance and determine whether this interaction disrupts STAT3 dimerization, a process mediated by reciprocal SH2-domain engagement between STAT3 monomers that would be expected to be impaired by occupancy of this domain by the *N*-sulfonyl-4-piperidone core fragment of **3a**, as predicted by molecular docking. In addition, because the thermal shift assay was performed in cell lysates, it demonstrates a direct interaction between **3a** and STAT3 but does not exclude additional contributions from upstream signaling in intact cells. Analysis of upstream kinases such as JAK2 and Src will therefore be required to define the mechanism of p-STAT3 suppression fully. These findings add to the current knowledge of other STAT3-inhibitory curcumin derivatives, since the compound class of curcuminoids is a treasure trove of potent STAT3 inhibitors [16]. Actually, STAT3 is a valuable drug target in KRAS-mutant PDAC because STAT3 expression and the hyper-activation of associated signaling pathways (e.g., JAK/STAT signaling, as well as STAT3 activation by upstream tyrosine kinases and mTOR) were correlated with vital mechanisms such as tumor cell survival, angiogenesis, metastasis formation, and immune escape [32]. Moreover, several STAT3 inhibitors have entered clinical trials for PDAC, underscoring STAT3 as an important drug target in PDAC [33]. STAT3-targeting drugs may be beneficial in combination with RAS and RTK inhibitors for treating PDAC [7]. STAT3 inhibitors may also prevent immune evasion and enhance the activity of immune therapies, which are particularly challenging in PDAC because it is classified as an immunologically “cold” tumor [34,35].

Hence, the tosylamide-based curcuminoid **3a** has the potential to become a valuable anticancer drug for the treatment of PDAC. While its activity as a single agent is already pronounced, studies with suitable combination partners, such as currently used PDAC therapies (e.g., FOLFIRINOX, gemcitabine, erlotinib) or emerging drug candidates such as RAS inhibitors, appear promising for future development [23,36]. The anti-PDAC activity of **3a** also warrants further studies in suitable in vivo models to determine its suitability as a new anticancer drug. In addition to PDACs, the previously reported broad-spectrum anticancer activity of **1a** against the NCI panel of cancer cell lines may suggest further cancer types, especially cancers dependent on oncogenic STAT3 (e.g., breast cancers, head-and-neck cancers, leukemias, lymphomas, and myelomas), as possible therapeutic targets of **3a** [18,37].

## 4. Materials and Methods

### 4.1. General Procedures

Column chromatography: silica gel 60 (230–400 mesh). Melting points (uncorrected), Electrothermal 9100 (ThermoFisher Scientific, Geel, Belgium); IR spectra, Perkin-Elmer Spectrum One FT-IR spectrophotometer with ATR sampling unit (Perkin Elmer, Rodgau, Germany); NMR spectra, Bruker Avance 300/500 spectrometer (Bruker, Billerica, MA, USA); chemical shifts (*δ*) are given in parts per million (ppm) downfield from tetramethylsilane as internal standard; Mass spectra, Thermo Finnigan MAT 8500 (EI) (ThermoFisher Scientific, Geel, Belgium); elemental analyses: Perkin-Elmer 2400 CHN elemental analyzer (Perkin Elmer, Rodgau, Germany).

### 4.2. Materials

Starting compounds and reagents were obtained from Alfa Aesar (Karlsruhe, Germany), Sigma-Aldrich (Darmstadt, Germany), and TCI (Zwijndrecht, Belgium). The curcuminoids **1a**, **3a–j**, and **4a** were prepared and published in previous studies [18,19]. The syntheses of the *N*-sulfonylpiperidin-4-one starting compounds **2a–h** and of curcuminoid **7** were also reported in previous studies [18,19].

### 4.3. Synthesis of New Curcuminoids

***(3E,5E)-3,5-Bis-(3,4-difluorobenzylidene)-1-(1-methoxybenzene-4-sulfonyl)-piperidone (4b)—Typical procedure***

1-(4-Methoxybenzenesulfonyl)-4-piperidinone (213 mg, 0.79 mmol) and 3,4-difluorobenzaldehyde (213 mg, 1.5 mmol) were dissolved in EtOH (10 mL) and conc. HCl (1 mL) was added. The reaction mixture was refluxed for 24 h. After cooling down to room temperature, the formed precipitate was collected, washed with EtOH and dried in vacuum. Yield: 125 mg (0.24 mmol, 31%); yellow solid of m.p. 238 °C; *υ*_max_(ATR)/cm^−1^ 3964, 2846, 1677, 1596, 1518, 1496, 1440, 1422, 1342, 1300, 1274, 1261, 1233, 1176, 1151, 1116, 1092, 1042, 1019, 997, 972, 950, 927, 876, 849, 830, 773, 721, 694, 675; ^1^H NMR (300 MHz, DMSO-d_6_) *δ* 3.86 (3 H. s), 4.61 (4 H, s), 7.05 (2 H, d, J = 9.0 Hz), 7.3–7.4 (2 H, m), 7.45 (2 H, d, J = 9.0 Hz), 7.52 (2 H, s), 7.6–7.7 (4 H, m); ^13^C NMR (75.5 MHz, DMSO-d_6_) *δ* 46.4, 55.8, 114.5, 118.0, 118.2, 119.3, 119.6, 127.6, 127.7, 128.9, 129.5, 131.5, 131.6, 135.1, 147.7, 147.8, 148.2, 148.4, 150.9, 151.1, 162.9, 183.9; *m*/*z* (%) 517 (7) [M^+^], 346 (100), 317 (6), 194 (7), 180 (16), 171 (15), 151 (23); anal. calcd. for C_26_H_19_F_4_NO_4_S: C, 60.35; H, 3.70. Found: C, 60.25; H, 3.65.

***(3E,5E)-3,5-Bis-(3,4-difluorobenzylidene)-1-(1-fluorobenzene-4-sulfonyl)-piperidone (4c)***

Analogously to the synthesis of **4b**, compound **4c** was obtained from 1-(4-fluorobenzenesulfonyl)-4-piperidinone (203 mg, 0.79 mmol) and 3,4-difluorobenzaldehyde (213 mg, 1.5 mmol) in EtOH (10 mL) and conc. HCl (1 mL). Yield: 244 mg (0.48 mmol, 61%); yellow solid of m.p. 202 °C; *υ*_max_(ATR)/cm^−1^ 3059, 2935, 2823, 1668, 1615, 1591, 1574, 1517, 1493, 1420, 1362, 1300, 1281, 1251, 1236, 1207, 1172, 1149, 1117, 1089, 1041, 1000, 974, 954, 936, 895, 848, 825, 791, 774, 741, 719, 698, 666, 602, 574; ^1^H NMR (500 MHz, DMSO-d_6_) *δ*4.67 (4 H, s), 7.3–7.4 (4 H, m), 7.51 (2 H, s), 7.6–7.9 (6 H, m); ^13^C NMR (125 MHz, DMSO-d_6_) *δ* 46.5, 116.6, 116.8, 118.1, 119.5, 119.7, 127.9, 130.5, 131.1, 131.6, 133.9, 135.4, 148.4, 148.5, 149.1, 150.4, 151.1, 163.8, 165.8, 183.9; *m*/*z* (%) 505 (28) [M^+^], 346 (100), 317 (37), 180 (50), 151 (92), 127 (26), 95 (39); anal. calcd. for C_25_H_16_F_5_NO_3_S: C, 59.41; H, 3.19. Found: C, 59.28; H, 3.14.

***(3E,5E)-3,5-Bis-(3,4-difluorobenzylidene)-1-(1-fluorobenzene-3-sulfonyl)-piperidone (4d)***

Analogously to the synthesis of **4b**, compound **4c** was obtained from 1-(3-fluorobenzenesulfonyl)-4-piperidinone (203 mg, 0.79 mmol) and 3,4-difluorobenzaldehyde (213 mg, 1.5 mmol) in EtOH (10 mL) and conc. HCl (1 mL). Yield: 160 mg (0.32 mmol, 40%); yellow solid of m.p. 203–204 °C; *υ*_max_(ATR)/cm^−1^ 3060, 1676, 1599, 1513, 1479, 1432, 1350, 1301, 1280, 1265, 1232, 1200, 1160, 1118, 1086, 1072, 1044, 994, 959, 944, 923, 881, 829, 812, 791, 777, 750, 718, 699, 688, 678, 638, 607; ^1^H NMR (300 MHz, DMSO-d_6_) *δ* 4.70 (4 H, s), 7.3–7.4 (4 H, m), 7.51 (2 H, s), 7.6–7.7 (6 H, m); ^13^C NMR (75.5 MHz, DMSO-d_6_) *δ* 46.3, 114.0–114.4 (m), 118.0–119.6 (m), 120.6–120.9 (m), 123.4, 127.7–127.9 (m), 131.4, 131.5–132.0 (m), 139.7, 147.7–151.8 (m), 159.8–163.3 (m), 184.0; *m*/*z* (%) 505 (22) [M^+^], 346 (100), 317 (23), 180 (30), 151 (74), 95 (36); anal. calcd. for C_25_H_16_F_5_NO_3_S: C, 59.41; H, 3.19. Found: C, 59.31; H, 3.13.

***(3E,5E)-3,5-Bis-(3,4-difluorobenzylidene)-1-(1-chlorobenzene-4-sulfonyl)-piperidone (4e)***

Analogously to the synthesis of **4b**, compound **4e** was obtained from 1-(4-chlorobenzenesulfonyl)-4-piperidinone (216 mg, 0.79 mmol) and 3,4-difluorobenzaldehyde (213 mg, 1.5 mmol) in EtOH (10 mL) and conc. HCl (1 mL). Yield: 123 mg (0.24 mmol, 30%); yellow solid of m.p. 232–233 °C; *υ*_max_(ATR)/cm^−1^ 3095, 2910, 1664, 1593, 1569, 1517, 1476, 1420, 1360, 1301, 1276, 1235, 1207, 1163, 1116, 1094, 1046, 1012, 996, 977, 950, 877, 848, 818, 775, 754, 717, 694, 649, 608; ^1^H NMR (300 MHz, DMSO-d_6_) *δ* 4.67 (4 H, s), 7.3–7.4 (2 H, m), 7.5–7.7 (10 H, m); ^13^C NMR (75.5 MHz, DMSO-d_6_) *δ* 46.4, 118.0, 118.2, 119.4, 119.7, 127.7, 127.8, 129.1, 129.6, 131.1, 131.5, 131.6, 135.4, 136.4, 138.5, 147.7, 147.9, 148.3, 148.5, 151.0, 151.1, 151.6, 151.8, 183.9; *m*/*z* (%) 523 (12) [M^+^], 521 (32) [M^+^], 346 (100), 317 (46), 180 (55), 152 (90), 151 (92), 111 (33); anal. calcd. for C_25_H_16_ClF_4_NO_3_S: C, 57.53; H, 3.09. Found: C, 57.39; H, 3.05.

***(3E,5E)-3,5-Bis-(3,4-difluorobenzylidene)-1-(1,2-dichlorobenzene-4-sulfonyl)-piperidone (4f)***

Analogously to the synthesis of **4b**, compound **4f** was obtained from 1-(3,4-dichlorobenzenesulfonyl)-4-piperidinone (243 mg, 0.79 mmol) and 3,4-difluorobenzaldehyde (213 mg, 1.5 mmol) in EtOH (10 mL) and conc. HCl (1 mL). Yield: 156 mg (0.28 mmol, 35%); yellow solid of m.p. 188–190 °C; ^1^H NMR (300 MHz, DMSO-d_6_) *δ* 4.74 (4 H, s), 7.3–7.4 (2 H, m), 7.4–7.7 (8 H, m), 7.84 (1 H, d, J = 8.5 Hz); ^13^C NMR (75.5 MHz, DMSO-d_6_) *δ* 46.4, 118.0–118.2 (m), 119.4–119.7 (m), 127.2, 127.9, 128.9, 130.9, 131.3–131.5 (m), 131.8, 132.3, 135.4, 136.7, 138.0, 147.7–151.8 (m), 183.9; *m*/*z* (%) 557 (11) [M^+^], 555 (13) [M^+^], 378 (2), 376 (3), 346 (100), 317 (17), 180 (24), 151 (42); anal. calcd. for C_25_H_15_Cl_2_F_4_NO_3_S: C, 53.97; H, 2.72. Found: C, 53.89; H, 2.67.

***(3E,5E)-3,5-Bis-(3,4-difluorobenzylidene)-1-(1,2-difluorobenzene-4-sulfonyl)-piperidone (4g)***

Analogously to the synthesis of **4b**, compound **4g** was obtained from 1-(3,4-difluorobenzenesulfonyl)-4-piperidinone (217 mg, 0.79 mmol) and 3,4-difluorobenzaldehyde (213 mg, 1.5 mmol) in EtOH (10 mL) and conc. HCl (1 mL). Yield: 128 mg (0.24 mmol, 32%); pale yellow solid of m.p. 193–194 °C; *υ*_max_(ATR)/cm^−1^ 3073, 2835, 1680, 1611, 1597, 1516, 1507, 1455, 1420, 1354, 1303, 1280, 1230, 1205, 1160, 1146, 1124, 1113, 1070, 1054, 993, 972, 952, 936, 914, 883, 871, 860, 839, 822, 790, 775, 735, 716, 694, 674, 638, 608; ^1^H NMR (300 MHz, DMSO-d_6_) *δ* 4.70 (4 H, s), 7.3–7.5 (3 H, m), 7.53 (2 H, s), 7.6–7.8 (6 H, m); ^13^C NMR (75.5 MHz, DMSO-d_6_) *δ* 46.4, 117.0–117.4 (m), 118.0–118.2 (m), 118.8–119.6 (m), 125.2–125.3 (m), 125.3, 127.8–127.9 (m), 131.1, 131.5, 134.8, 135.3, 147.7–151.8 (m), 184.0; *m*/*z* (%) 523 (37) [M^+^], 346 (100), 317 (28), 180 (35), 151 (67); anal. calcd. for C_25_H_15_F_6_NO_3_S: C, 57.37; H, 2.89. Found: C, 57.29; H, 2.84.

***(3E,5E)-3,5-Bis-(3,4-difluorobenzylidene)-1-cinnamoylpiperidone (4h)***

Compound **7** (112 mg, 0.32 mmol) was suspended in acetone and treated with cinnamoyl chloride (82 mg, 0.49 mmol). K_2_CO_3_ (223 mg, 1.61 mmol, dissolved in 2 mL H_2_O) was added and the reaction mixture was stirred at room temperature for 24 h. Water (20 mL) was added and the formed precipitate was collected, washed with water and water/methanol and dried in vacuum. Yield: 115 mg (0.24 mmol, 75%); yellow solid of m.p. 169–170 °C; *υ*_max_(ATR)/cm^−1^ 3062, 1679, 1645, 1602, 1513, 1454, 1431, 1326, 1301, 1265, 1234, 1212, 1195, 1168, 1150, 1115, 997, 958, 940, 927, 879, 853, 826, 794, 762, 744, 706, 681; ^1^H NMR (300 MHz, CDCl_3_) *δ* 4.8–4.9 (4 H, br s), 6.41 (1 H, d, J = 15.4 Hz), 7.1–7.3 (11 H, m), 7.52 (1 H, d, J = 15.4 Hz), 7.72 (2 H, s); ^13^C NMR (75.5 MHz, CDCl_3_) *δ* 46.1, 115.8, 117.9, 118.1, 119.0, 119.2, 126.9, 127.6, 128.8, 130.1, 131.5, 132.7, 134.6, 135.9, 144.0, 148.7, 148.9, 149.3, 149.4, 152.0, 152.6, 152.8, 165.8, 186.4; *m*/*z* (%) 477 (96) [M^+^], 346 (46), 151 (14), 131 (100), 103 (54). 77 (16); anal. calcd. for C_28_H_19_F_4_NO_2_: C, 70.44; H, 4.01. Found: C, 70.31; H, 3.97.

***(3E,5E)-3,5-Bis-(3,5-difluorobenzylidene)-1-(1-methoxybenzene-4-sulfonyl)-piperidone (5b)***

Analogously to the synthesis of **4b**, compound **5b** was obtained from 1-(4-methoxybenzenesulfonyl)-4-piperidinone (213 mg, 0.79 mmol) and 3,5-difluorobenzaldehyde (213 mg, 1.5 mmol) in EtOH (10 mL) and conc. HCl (1 mL). Yield: 130 mg (0.25 mmol, 32%); yellow solid of m.p. 241–242 °C; *υ*_max_(ATR)/cm^−1^ 3088, 2964, 2841, 1672, 1615, 1592, 1497, 1471, 1434, 1383, 1350, 1315, 1253, 1236, 1220, 1188, 1165, 1152, 1122, 1094, 1051, 1034, 1024, 997, 963, 944, 887, 845, 803, 773, 750, 719, 671, 647, 630; ^1^H NMR (300 MHz, DMSO-d_6_) *δ* 3.86 (3 H, s), 4.63 (4 H, s), 7.04 (2 H, d, J = 9.0 Hz), 7.2–7.3 (4 H, m), 7.3–7.5 (4 H, m), 7.52 (2 H, s); ^13^C NMR (75.5 MHz, DMSO-d_6_) *δ* 46.3, 55.8, 104.7–105.4 (m), 113.1–113.3 (m), 114.6, 128.9, 129.5, 132.6, 135.0, 137.2, 137.4, 160.6–160.8 (m), 163.0, 163.9–164.1 (m), 183.9; *m*/*z* (%) 517 (31) [M^+^], 346 (100), 171 (33), 151 (51), 107 (23), 77 (20); anal. calcd. for C_26_H_19_F_4_NO_4_S: C, 60.35; H, 3.70. Found: C, 60.28; H, 3.63.

***(3E,5E)-3,5-Bis-(3,5-difluorobenzylidene)-1-(1-fluorobenzene-4-sulfonyl)-piperidone (5c)***

Analogously to the synthesis of **4b**, compound **5c** was obtained from 1-(4-fluorobenzenesulfonyl)-4-piperidinone (203 mg, 0.79 mmol) and 3,5-difluorobenzaldehyde (213 mg, 1.5 mmol) in EtOH (10 mL) and conc. HCl (1 mL). Yield: 120 mg (0.24 mmol, 30%); yellow solid of m.p. 264–265 °C; *υ*_max_(ATR)/cm^−1^ 3077, 1677, 1617, 1590, 1493, 1468, 1433, 1380, 1352, 1314, 1289, 1238, 1219, 1168, 1157, 1121, 1088, 1051, 1031, 999, 959, 949, 930, 880, 867, 851, 832, 809, 757, 739, 716, 678, 665, 646, 624; ^1^H NMR (300 MHz, DMSO-d_6_) *δ* 4.70 (4 H, s), 7.2–7.3 (4 H, m), 7.3–7.4 (4 H, m), 7.51 (2 H, s), 7.5–7.6 (2 H, m); ^13^C NMR (75.5 MHz, DMSO-d_6_) *δ* 46.4, 104.8–105.5 (m), 113.2–113.5 (m), 116.5–116.8 (m), 130.3, 132.3, 134.0, 135.2, 137.0, 137.3, 160.6–160.8 (m), 163.1, 163.9–164.1 (m), 166.4, 183.8; *m*/*z* (%) 505 (24) [M^+^], 346 (100), 180 (13), 151 (46), 95 (15); anal. calcd. for C_25_H_16_F_5_NO_3_S: C, 59.41; H, 3.19. Found: C, 59.27; H, 3.11.

***(3E,5E)-3,5-Bis-(3,5-difluorobenzylidene)-1-(1-fluorobenzene-3-sulfonyl)-piperidone (5d)***

Analogously to the synthesis of **4b**, compound **5d** was obtained from 1-(3-fluorobenzenesulfonyl)-4-piperidinone (166 mg, 0.65 mmol) and 3,5-difluorobenzaldehyde (174 mg, 1.23 mmol) in EtOH (10 mL) and conc. HCl (1 mL). Yield: 107 mg (0.21 mmol, 32%); yellow solid of m.p. 185–187 °C; *υ*_max_(ATR)/cm^−1^ 3080, 1676, 1616, 1607, 1591, 1477, 1435, 1351, 1313, 1273, 1234, 1217, 1162, 1120, 1082, 1051, 1028, 998, 959, 948, 897, 866, 841, 815, 787, 756, 739, 714, 695, 676, 667, 636; ^1^H NMR (300 MHz, DMSO-d_6_) *δ* 4.72 (4 H, s), 7.2–7.4 (8 H, m), 7.51 (2 H, s), 7.6–7.7 (2 H, m); ^13^C NMR (75.5 MHz, DMSO-d_6_) *δ* 46.3, 104.9–105.5 (m), 113.2–113.5 (m), 114.1–114.4 (m), 120.7–121.0 (m), 123.3, 131.9, 132.3, 135.1, 137.0–137.3 (m), 139.8, 160.0–164.1 (m), 184.0; *m*/*z* (%) 505 (60) [M^+^], 346 (100), 180 (33), 151 (96), 95 (41); anal. calcd. for C_25_H_16_F_5_NO_3_S: C, 59.41; H, 3.19. Found: C, 59.33; H, 3.16.

***(3E,5E)-3,5-Bis-(3,5-difluorobenzylidene)-1-(1-chlorobenzene-4-sulfonyl)-piperidone (5e)***

Analogously to the synthesis of **4b**, compound **5e** was obtained from 1-(4-chlorobenzenesulfonyl)-4-piperidinone (216 mg, 0.79 mmol) and 3,5-difluorobenzaldehyde (213 mg, 1.5 mmol) in EtOH (10 mL) and conc. HCl (1 mL). Yield: 130 mg (0.25 mmol, 32%); yellow solid of m.p. 261 °C; *υ*_max_(ATR)/cm^−1^ 3087, 1679, 1617, 1591, 1475, 1433, 1396, 1352, 1319, 1280, 1237, 1220, 1168, 1120, 1090, 1048, 1032, 1011, 997, 956, 862, 848, 835, 808, 755, 714, 669, 655, 647, 601; ^1^H NMR (300 MHz, DMSO-d_6_) *δ* 4.70 (4 H, s), 7.2–7.3 (4 H, m), 7.3–7.4 (2 H, m), 7.5–7.6 (4 H, m), 7.6–7.7 (2 H, m); ^13^C NMR (75.5 MHz, DMSO-d_6_) *δ* 46.4, 104.8–105.5 (m), 113.2–113.5 (m), 129.1, 129.6, 132.3, 136.5, 137.0, 138.5, 160.6–160.8 (m), 163.9–164.1 (m), 183.9; *m*/*z* (%) 523 (9) [M^+^], 521 (23) [M^+^], 346 (100), 180 (15), 151 (51), 111 (15); anal. calcd. for C_25_H_16_ClF_4_NO_3_S: C, 57.53; H, 3.09. Found: C, 57.44; H, 3.06.

***(3E,5E)-3,5-Bis-(3,5-difluorobenzylidene)-1-(1,2-dichlorobenzene-4-sulfonyl)-piperidone x EtOH (5f)***

Analogously to the synthesis of **4b**, compound **5f** was obtained from 1-(3,4-dichlorobenzenesulfonyl)-4-piperidinone (243 mg, 0.79 mmol) and 3,5-difluorobenzaldehyde (213 mg, 1.5 mmol) in EtOH (10 mL) and conc. HCl (1 mL). Yield: 142 mg 602.43 (0.24 mmol, 30%); yellow solid of m.p. 196–197 °C; *υ*_max_(ATR)/cm^−1^ 3550, 3101, 3049, 1004, 2974, 2921, 2864, 1675, 1612, 1598, 1511, 1474, 1432, 1371, 1351, 1319, 1240, 1222, 1170, 1146, 1122, 1101, 1069, 1049, 1031, 998, 956, 930, 876, 858, 826, 805, 758, 739, 714, 701, 666, 657, 613; ^1^H NMR (300 MHz, DMSO-d_6_) *δ* 1.05 (3 H, t, J = 6.2 Hz), 3.3–3.5 (2 H, m), 4.34 (1 H, t, J = 5.1 Hz), 4.75 (4 H, s), 7.2–7.3 (4 H, m), 7.4–7.6 (5 H, m), 7.68 (1 H, s), 7.86 (1 H, d, J = 8.5 Hz); ^13^C NMR (75.5 MHz, DMSO-d_6_) *δ* 18.5, 46.4, 56.0, 104.9–105.6 (m), 113.3–113.6 (m), 127.2, 128.9, 131.8, 132.1–132.3 (m), 135.2, 136.8–137.2 (m), 138.1, 160.6–164.1 (m), 183.9; *m*/*z* (%) 557 (17) [M^+^ − EtOH], 555 (25) [M^+^ − EtOH], 346 (97), 180 (43), 151 (100), 145 (40), 127 (34); anal. calcd. for C_27_H_21_Cl_2_F_4_NO_4_S: C, 53.83; H, 3.51. Found: C, 53.71; H, 3.48.

***(7E,9E)-7,9-Bis-(3,4-difluorobenzylidene)-1,4-dioxaspiro[4.5]decan-8-one (6b)—Typical procedure***

1,4-Cyclohexanedione monoethylene acetal (156 mg, 1 mmol) and 3,4-difluorobenzaldehyde (284 mg, 2.0 mmol) were dissolved in methanol (10 mL) and NaOH (200 mg, dissolved in 2 mL H_2_O) was added. The reaction mixture was stirred at room temperature for 1 h. The formed precipitate was collected and dried in vacuum. Yield: 177 mg (0.44 mmol, 44%); yellow solid of m.p. 186–188 °C; *ν*_max_ (ATR)/cm^−1^: 3049, 2970, 2946, 2879, 1677, 1611, 1597, 1515, 1422, 1341, 1306, 1264, 1227, 1216, 1200, 1150, 1116, 1101, 1045, 997, 968, 946, 929, 895, 876, 834, 820, 787, 775, 751, 712, 693, 620; ^1^H NMR (300 MHz, DMSO-d_6_): δ 3.1–3.2 (4 H, m), 3.8–3.9 (4 H, m), 7.4–7.6 (4 H, m), 7.6–7.7 (4 H, m); ^13^C NMR (75 MHz, DMSO-d_6_): δ 37.2, 64.3, 105.8, 117.6–119.2 (m), 127.5–127.6 (m), 132.4–132.6 (m), 133.8, 134.3, 135.3, 147.5–148.0 (m), 150.8–151.3 (m), 187.1; *m*/*z* (%) 404 (96) [M^+^], 319 (23), 280 (30), 223 (22), 151 (72), 141 (32), 99 (100); anal. calcd. for C_22_H_16_F_4_O_3_: C, 65.35; H, 3.99. Found: C, 65.27; H, 3.94.

***(7E,9E)-7,9-Bis-(3,5-difluorobenzylidene)-1,4-dioxaspiro[4.5]decan-8-one (6c)***

Analogously to the synthesis of **6b**, compound **6c** was obtained from 1,4-cyclohexanedione monoethylene acetal (156 mg, 1 mmol) and 3,5-difluorobenzaldehyde (284 mg, 2.0 mmol) in methanol (10 mL) and NaOH (200 mg, dissolved in 2 mL H_2_O). Yield: 163 mg (0.40 mmol, 40%); yellow solid of m.p. 186–188 °C; *ν*_max_ (ATR)/cm^−1^: 3079, 2994, 2952, 2880, 1677, 1619, 1593, 1471, 1434, 1345, 1322, 1269, 1235, 1214, 1151, 1120, 1075, 1048, 1030, 1011, 990, 976, 949, 932, 920, 864, 848, 827, 772, 753, 708, 676, 645, 609; ^1^H NMR (300 MHz, DMSO-d_6_): δ 3.1–3.2 (4 H, m), 3.8–3.9 (4 H, m), 7.2–7.3 (6 H, m), 7.63 (2 H, s); ^13^C NMR (75 MHz, DMSO-d_6_): δ 37.0, 64.3, 104.1–104.7 (m), 105.7, 112.9–113.3 (m), 135.1, 138.1–138.4 (m), 160.5–164.0 (m), 187.1; *m*/*z* (%) 404 (52) [M^+^], 280 (46), 151 (41), 141 (46), 99 (100), 86 (19), 55 (24); anal. calcd. for C_22_H_16_F_4_O_3_: C, 65.35; H, 3.99. Found: C, 65.22; H, 3.91.

### 4.4. Anticancer Activity

#### 4.4.1. Cell Lines and Culture Conditions

The pancreatic cancer cell lines MiaPaCa-2 (ATCC CRL-1420), S2-007 (RRID: CVCL_B279; a clone of the SUIT-2 line originally established by Iwamura et al.) [38], and Panc-1 (ATCC CRL-1469), and the human leukemia cell lines THP-1 (ATCC TIB-202) and Jurkat (clone E6-1, ATCC TIB-152) were provided as a gift by Dr. Shrikant Anant’s laboratory. MiaPaCa-2, S2-007, and Panc-1 were cultured in complete Dulbecco’s Modified Eagle Medium (DMEM; Corning, Tewksbury, MA, USA), supplemented with 4.5 g/L glucose, L-glutamine, and sodium pyruvate; 10% heat-inactivated fetal bovine serum (FBS; Sigma-Aldrich, St. Louis, MO, USA); and 1% antibiotic–antimycotic solution (Corning, Tewksbury, MA, USA). THP-1 and Jurkat cells were cultured in complete RPMI-1640 medium prepared the same way. All cell lines were maintained at 37 °C in a humidified incubator with 5% CO_2_ and used within 15 passages.

#### 4.4.2. Antiproliferation Assay

A total of 5 × 10^3^ MiaPaCa-2, S2-007, and Panc-1 cells/well were added to 96-well plates. After 24 h of plating, the cells were treated with increasing concentrations of curcuminoids (0–10 µM). An enzymatic hexosaminidase assay was used to record the viability of pancreatic cancer cells at different time points [39]. For THP-1 and Jurkat cells, a CCK-8 assay (Abcam #ab228554, Waltham, MA, USA) was used [40]. Briefly, 1 × 10^4^ THP-1 and Jurkat cells/well were plated and treated with curcuminoids after 24 h of plating. The CCK-8 reagent was incubated with THP-1 and Jurkat cells for 60 min at 37 °C, and the absorbance (OD value) was recorded at 450 nm. Percent inhibition of proliferation was estimated by comparing cell viability in the treated groups with that of untreated cells.

#### 4.4.3. Spheroid Growth Inhibition Assay

In ultra-low-attachment plates (Corning, Tewksbury, MA, USA), 500 MiaPaCa-2 or S2-007 cells were seeded in each well with spheroid medium [DMEM supplemented with EGF (20 ng/mL), FGF (20 ng/mL), heparin (4 µg/mL), B27 supplement (50×; 10 mL per 500 mL medium; Invitrogen, Carlsbad, CA, USA), and 1% penicillin/streptomycin]. After 24 h, spheroids were treated with **3a** at its respective IC_50_ concentration. Spheroids were counted and imaged after 7 days [41]. To evaluate self-renewal, primary spheroids from vehicle- and **3a**-treated wells were collected, dissociated into single cells with trypsin, counted, and re-plated at 500 cells per well in fresh spheroid medium without **3a**. Secondary spheroids were counted and imaged after 5 days [30,31].

#### 4.4.4. Colony Formation Assay

In total, 500 S2-007 cells and MiaPaCa-2 cells were seeded in each well of 6-well plates. Cells were allowed to attach for 24 h before treatment with **3a** at its IC_50_ and ½IC_50_ concentrations. After 48 h, the drug-containing medium was replaced with drug-free medium to terminate drug exposure, and cells were allowed to grow and form colonies for 10–12 days. At the end of the experiment, colonies were washed with PBS three times, fixed with 10% formalin solution (Fisher Scientific #23-305510, Pittsburgh, PA, USA), and stained with 1% crystal violet (Sigma-Aldrich#C6158, St. Louis, MO, USA) in 10% ethanol for 15 min. Colonies were then washed with water, dried at room temperature, counted, and imaged. The number of colonies in treated cells was compared with untreated cells [41].

#### 4.4.5. Apoptosis Assay

For Annexin V/PI staining, 1×10^5^ MiaPaCa-2 or Panc-1 cells were seeded in 6-well plates and allowed to attach for 24 h. The cells were then treated with vehicle (DMSO) or **3a** at IC_50_ (260 nM MiaPaCa-2, 560 nM Panc-1) for 48 h. Floating and adherent cells were trypsinized, collected, washed twice with cold PBS, and resuspended in Annexin V binding buffer. The cells were stained with Annexin V-FITC and propidium iodide (PI) using the Dead Cell Apoptosis Kit with Annexin V for Flow Cytometry (ThermoFisher, catalog number V13242), according to the manufacturer’s instructions and incubated for 15 min at room temperature in the dark. Samples were analyzed on a Attune NxT flow cytometer (ThermoFisher Scientific, Waltham, MA, USA), acquiring 10,000 events per sample. Data were analyzed with FlowJo Version 10.10.2 (BD Biosciences, Ashland, OR, USA). Cells were classified as live (Annexin V^−^/PI^−^), early apoptotic (Annexin V^+^/PI^−^), late apoptotic (Annexin^+^/PI^+^), or necrotic (Annexin V^−^/PI^+^) [30].

To study the caspase3/7 activity in pancreatic cancer cells (MiaPaCa-2 and S2-007) after **3a** treatment, we used the Apo-ONE Homogeneous Caspase-3/7 Assay kit (Promega Corporation, Madison, WI, USA). We followed the manufacturer’s instructions to perform this assay.

#### 4.4.6. Western Blot

A total of 5 × 10^5^ MiaPaCa-2 and S2-007 cells were seeded in 10 cm dishes for Western blot analysis. Twenty-four hours after plating, cells were treated with vehicle or **3a** at its IC_50_ concentration. After 24 h and 48 h of treatment, media were aspirated, and cells were washed with PBS, lysed, and sonicated in lysis buffer containing a protease inhibitor cocktail (Thermo Scientific, Rockford, IL, USA). Cell lysates were centrifuged at 6000 rpm for 10 min at 4 °C, and total protein concentration was determined using the BCA method (Thermo Scientific, Rockford, IL, USA). Equal amounts of protein were separated by SDS-PAGE and transferred onto PVDF membranes (Immobilon, Millipore, Bedford, MA, USA) for 2 h at 90 V. Membranes were blocked in 5% skimmed milk for 1 h and washed three times with TBST for 5 min each. Membranes were then incubated with primary antibodies overnight at 4 °C on a shaker, washed the next day, probed with the appropriate secondary antibodies for 1 h, and washed again before chemiluminescent detection. Blots were developed using chemiluminescence reagents (GE Healthcare, Piscataway, NJ, USA) and imaged using a ChemiDoc-XRS+ instrument (Bio-Rad Laboratories, Hercules, CA, USA); protein levels were quantified using Image Lab software (Image Lab 6.1, Bio-Rad Laboratories, Inc.). Bcl-2 (CST#4223), Bax (CST#2772), Bcl-Xl (CST #2762), p-STAT3 (CST#4113S), total STAT3 (CST#9139), and cyclin D1 (CST#55506) antibodies were obtained from Cell Signaling Technology (CST; Beverly, MA, USA); GAPDH (sc-365062) antibody was obtained from Santa Cruz Biotechnology (Santa Cruz, CA, USA). All antibodies were used at a 1:1000 dilution in 5% BSA in TBST.

#### 4.4.7. Cellular Thermal Shift Assay (CETSA)

Cellular thermal shift assay (CETSA) was used to assess how compound **3a** affects the stability of STAT3 in PDAC cells, specifically MiaPaCa-2 cells. Initially, cells were cultured until they reached 70–80% confluency. Following growth, cells were prepared using a lysis buffer. Further, cells were centrifuged at 4 °C for 10 min at 6000 rpm. The lysates were then treated with either DMSO (as a control, <1% concentration) or compound **3a** at 20 µM for 4 h on a rotating shaker. Post-treatment, the lysates were transferred to polymerase chain reaction (PCR) tubes and heated for 3 min at various temperature gradients (40–58 °C, 2 °C intervals) to denature the proteins. This was followed by centrifugation for 20 min to separate the soluble fractions from the precipitated proteins. The supernatant was carefully collected, mixed with 2X Laemmli buffer, and then boiled at 70 °C for 10 min. A Western blot was performed using an STAT3 antibody (CST #9139,1:1000 dilution) [30,31,42]. Band intensities were quantified with Image Lab software and normalized to the 44 °C sample of the respective group.

### 4.5. Molecular Docking

Molecular docking was performed using AutoDock Vina 1.1.2 to study the interaction of active curcuminoids with the STAT3 protein (PDB ID: 6NJS) [43,44]. This PDB structure has a resolution of 2.70 Å and contains a co-crystallized ligand, making it well suited for studying STAT3 inhibitors [45]. The 3D grid was designed around the SH2 domain, encompassing the amino acids interacting with the co-crystallized ligand, with a grid spacing of 1.0 Å and dimensions of 56 × 82 × 68 points (Å). Preparation of the curcuminoid ligand and the STAT3 protein was conducted using the default parameters of AutoDock Tools 1.5.7 (MGLTools); Kollman united-atom charges were added to the STAT3 protein and Gasteiger charges to the curcuminoid ligand. Docking was performed using AutoDock Vina’s default search algorithm. Ten predicted conformations were generated, and the most favorable conformation located within the SH2 domain was selected on the basis of binding energy and hydrogen bond formation. The **3a** (A19)–STAT3 complexes were visualized using the educational PyMOL program (https://pymol.org/2/, accessed 28 July 2026) [46]. The docking protocol was validated by redocking the co-crystallized ligand of 6NJS under identical conditions; a near-native pose was recovered among the generated solutions (heavy-atom RMSD 1.83 Å; 0.29 Å for the anchoring phosphonate group).

### 4.6. Statistical Analysis

All quantitative data are expressed as mean ± standard deviation (SD) unless otherwise noted. Statistical analyses were performed using GraphPad Prism (version 10.6.1; GraphPad Software, Boston, MA, USA). For comparisons between two groups (e.g., vehicle-treated vs. **3a**-treated), an unpaired two-tailed Student’s *t*-test was used. For comparisons among three or more groups, a one-way analysis of variance (ANOVA) was performed followed by Bonferroni–Šídák correction for multiple comparisons. CETSA melting curves were fitted by nonlinear regression (Boltzmann sigmoidal equation) to determine the melting temperature (T_m_). Curves for vehicle- and **3a**-treated cells were compared with the extra sum-of-squares F-test, and individual temperatures were compared by unpaired two-tailed Student’s *t*-test. Densitometric data were normalized to the respective loading control (GAPDH or total STAT3) and expressed relative to the vehicle control. Unless otherwise stated, experiments were performed in three independent replicates; the number of replicates (n) and what each data point represents are given in the figure legends. *p*-value < 0.05 was considered to represent statistical significance; exact *p*-values are reported in the figures.

## 5. Conclusions

This study identified the *N*-tosyl-substituted curcuminoid **3a** as a promising agent against KRAS-mutant PDAC based on its strong antiproliferative and cytotoxic activities, and on its notable STAT3-inhibitory mechanism. Thus, this work supports two options. On the one hand, **3a** could be developed as a new treatment for PDAC, which would require additional pre-clinical experiments, including in vivo models. On the other hand, the structure of **3a** can be further chemically optimized or formulated to meet criteria associated with general toxicity, pharmacokinetics, and/or bioavailability, if needed. Both options can pave the way to a promising new therapy for PDACs.

## Figures and Tables

**Table 1:** Antiproliferative activity of test compounds against MiaPaCa-2 and S2-007 pancreatic cancer cells, expressed as IC_50_ (µM) ^1^ after 48 h of treatment, as determined by a hexosaminidase assay. 5-Fluorouracil (5-FU) served as a positive control.

Compound	MiaPaCa-2	S2-007
**3a**	0.26 ± 0.13	0.22 ± 0.05
**3b**	>10	1.18 ± 0.92
**3c**	1.54 ± 0.31	1.54 ± 0.28
**3d**	0.73 ± 0.28	0.97 ± 0.42
**3e**	0.47 ± 0.01	0.55 ± 0.06
**3f**	2.11 ± 0.06	4.08 ± 0.46
**3g**	1.53 ± 0.38	2.70 ± 0.81
**3h**	>10	3.03 ± 2.11
**3i**	1.43 ± 0.39	3.25 ± 0.36
**3j**	1.13 ± 0.30	1.54 ± 0.20
**4a**	0.83 ± 0.18	0.55 ± 0.04
**4b**	0.73 ± 0.14	0.58 ± 0.04
**4c**	0.81 ± 0.06	1.06 ± 0.08
**4d**	0.71 ± 0.17	0.94 ± 0.36
**4e**	0.44 ± 0.06	0.54 ± 0.08
**4f**	0.49 ± 0.04	0.53 ± 0.03
**4g**	0.99 ± 0.12	1.16 ± 0.24
**4h**	1.34 ± 0.32	2.02 ± 0.82
**1a**	0.70 ± 0.23	0.67 ± 0.14
**5b**	3.92 ± 0.61	3.76 ± 1.07
**5c**	1.94 ± 0.54	2.41 ± 0.74
**5d**	>10	>10
**5e**	1.65 ± 0.26	0.76 ± 0.17
**5f**	3.61 ± 0.39	1.63 ± 0.56
**6a**	>10	4.68 ± 4.21
**6b**	>10	>10
**6c**	>10	>10
**5-FU**	2.19 ± 0.09	2.84 ± 0.42

^1^ IC_50_ values ± SD were calculated from the dose–response curves of three independent experiments.

**Table 2:** Time-dependent antiproliferative activity of **3a** against KRAS-mutant PDAC cells (MiaPaCa-2, S2-007, Panc-1) and the human leukemia cell lines Jurkat and THP-1 cells, expressed as IC_50_ (µM) ^1^, as determined by a hexosaminidase assay (PDAC lines) or CCK-8 assay (Jurkat and THP-1 cells).

Time	MiaPaCa-2	S2-007	Panc-1	Jurkat	THP-1
24 h	2.09 ± 0.46	>2.5	>2.5	>10	>10
48 h	0.26 ± 0.13 ^2^	0.22 ± 0.05 ^2^	0.56 ± 0.044	5.59 ± 0.84	9.23 ± 0.21
72 h	0.14 ± 0.01	0.13 ± 0.01	0.13 ± 0.01	3.44 ± 0.25	4.75 ± 0.15

^1^ IC_50_ values ± SD were calculated from the dose–response curves of three independent experiments. ^2^ See Table 1 for these 48 h values of **3a**.

## Data Availability

Original data associated with this article can be obtained from the authors upon reasonable request.

## Supplementary Materials

The following supporting information can be downloaded at https://www.mdpi.com/article/10.3390/ijms272110013/s1.

## Abbreviations

The following abbreviations are used in this manuscript:

Bax	Bcl-2-associated X protein
Bcl-2	B-cell lymphoma 2
EGFR	Epidermal growth factor receptor
FOLFIRINOX	Folinic acid plus 5-FU plus irinotecan plus oxaliplatin
5-FU	5-Fluorouracil
HER2	Human epidermal growth factor receptor 2
JAK	Janus kinase
PDAC	Pancreatic ductal adenocarcinoma
KRAS	Kirsten rat sarcoma
SH2	Src homology 2
STAT3	Signal transducer and activator of transcription 3
