# Supplementary material for: Activity of Fluoro-Substituted 3,5-Di[(E)-Benzylidene]piperidin-4-Ones with N-Arylsulfonyl Appendages Against KRAS-Mutant Pancreatic Ductal Adenocarcinoma Cells
Source: Int J Mol Sci. 2026 Nov 8;27(21):10013. doi: 10.3390/ijms272110013 (PMC13763989; doi:10.3390/ijms272110013)
Supplement: Supplementary file 1 [file ijms-27-10013-s001.zip › ijms-4610666-supplementary.pdf]

Supplementary Data

Activity of Fluoro-Substituted 3,5-Di[(*E*)-Benzylidene]piperidin-4-ones with *N*-Arylsulfonyl Appendages Against KRAS-Mutant Pancreatic Ductal Adenocarcinoma Cells

Hindole Ghosh [1], Duha Qamar Azaz [1], Addhyan Saxena [1], Nicole Bodenhausen [1], Rainer Schobert [2], Bernhard Biersack [2,*] and Prasad Dandawate [1,*]

[1] Cancer Biology, University of Kansas Medical Center, 3901 Rainbow Boulevard, Kansas City, KS 66160, USA; hghosh@kumc.edu (H.G.); duhaq.azaz@gmail.com (D.Q.A.);
addhyansaxena@gmail.com (A.S.); nibo2020@icloud.com (N.B.)

[2] Organic Chemistry 1, University of Bayreuth, Universitaetsstrasse 30, 95440 Bayreuth, Germany;
rainer.schobert@uni-bayreuth.de

* Correspondence: bernhard.biersack@yahoo.com (B.B.); pdandawate@kumc.edu (P.D.)

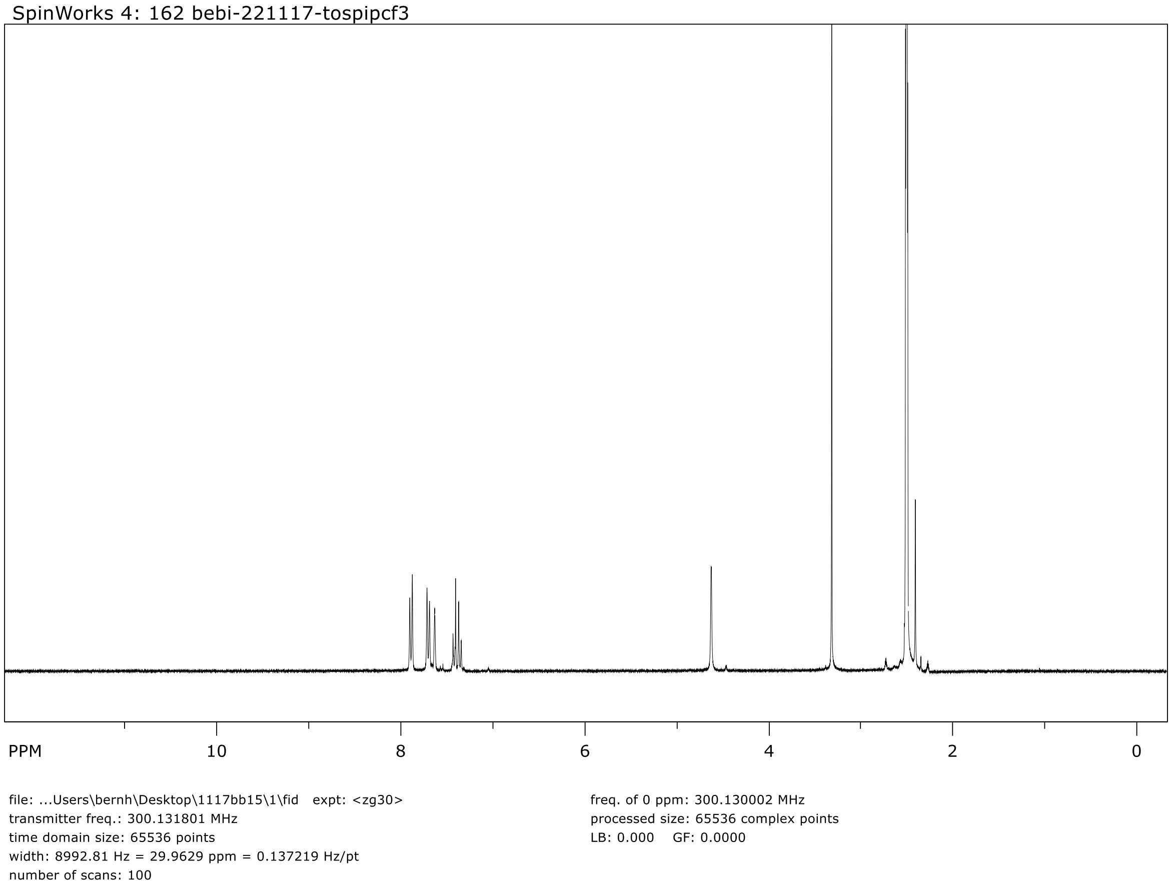

Figure S1. ^{1}H NMR spectrum of **3a**.

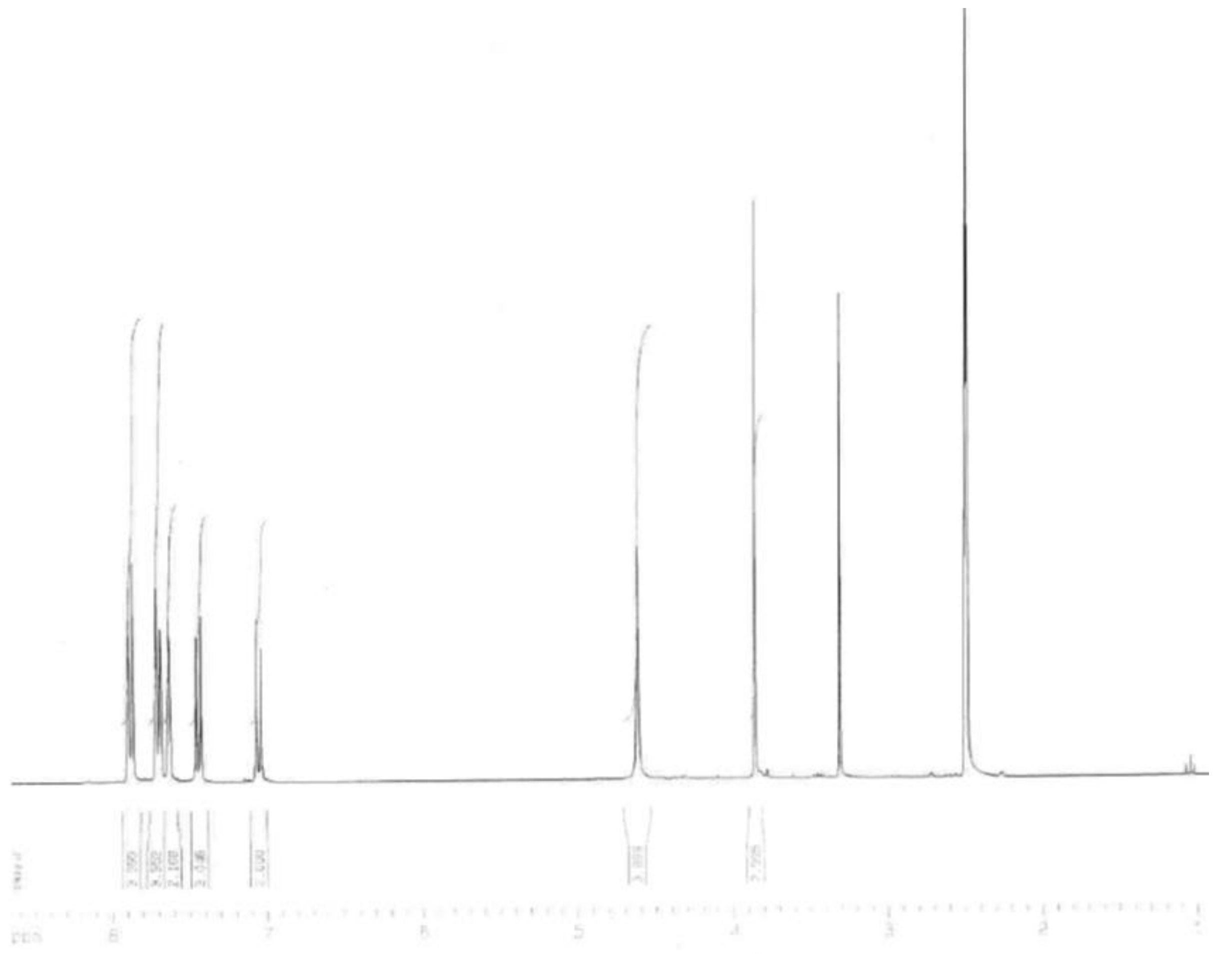

Figure S2. ^{1}H NMR spectrum of **3b**.

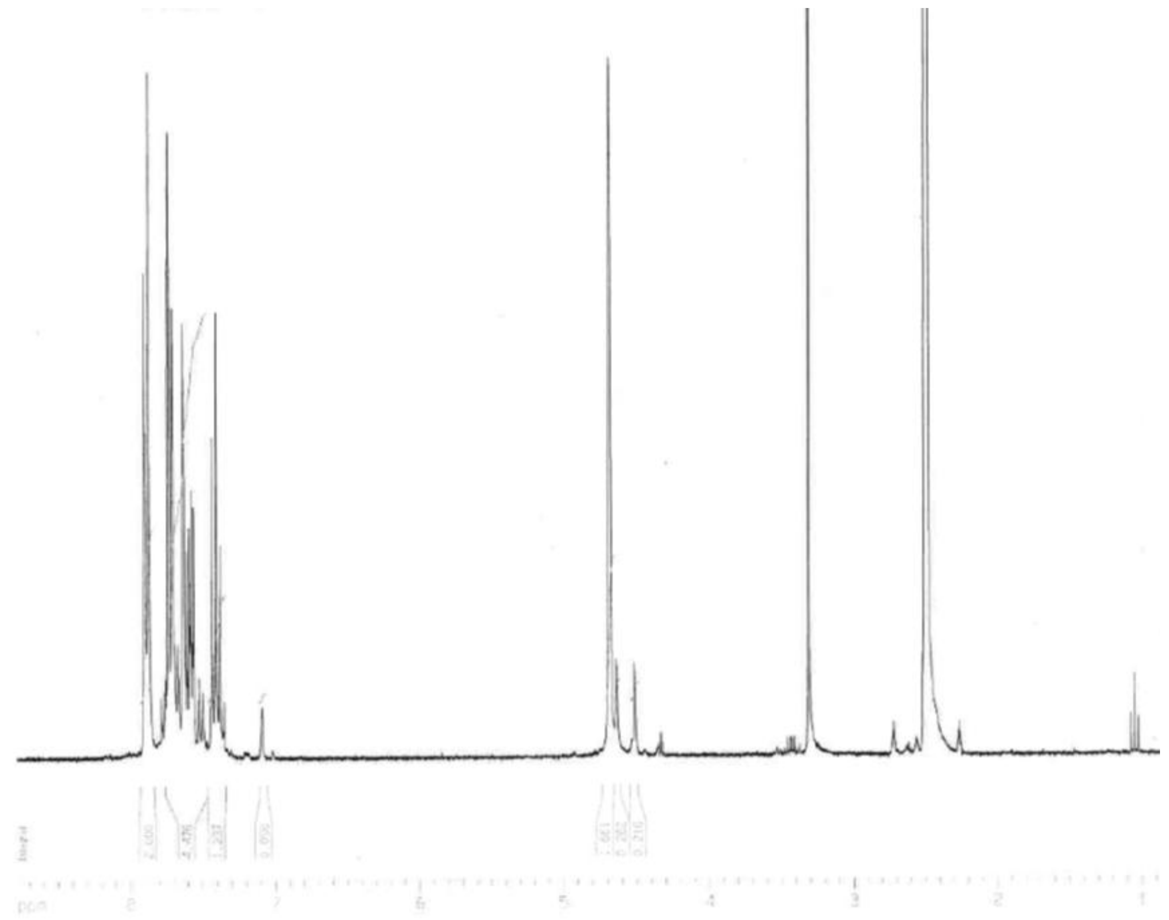

Figure S3. ^{1}H NMR spectrum of **3c**.

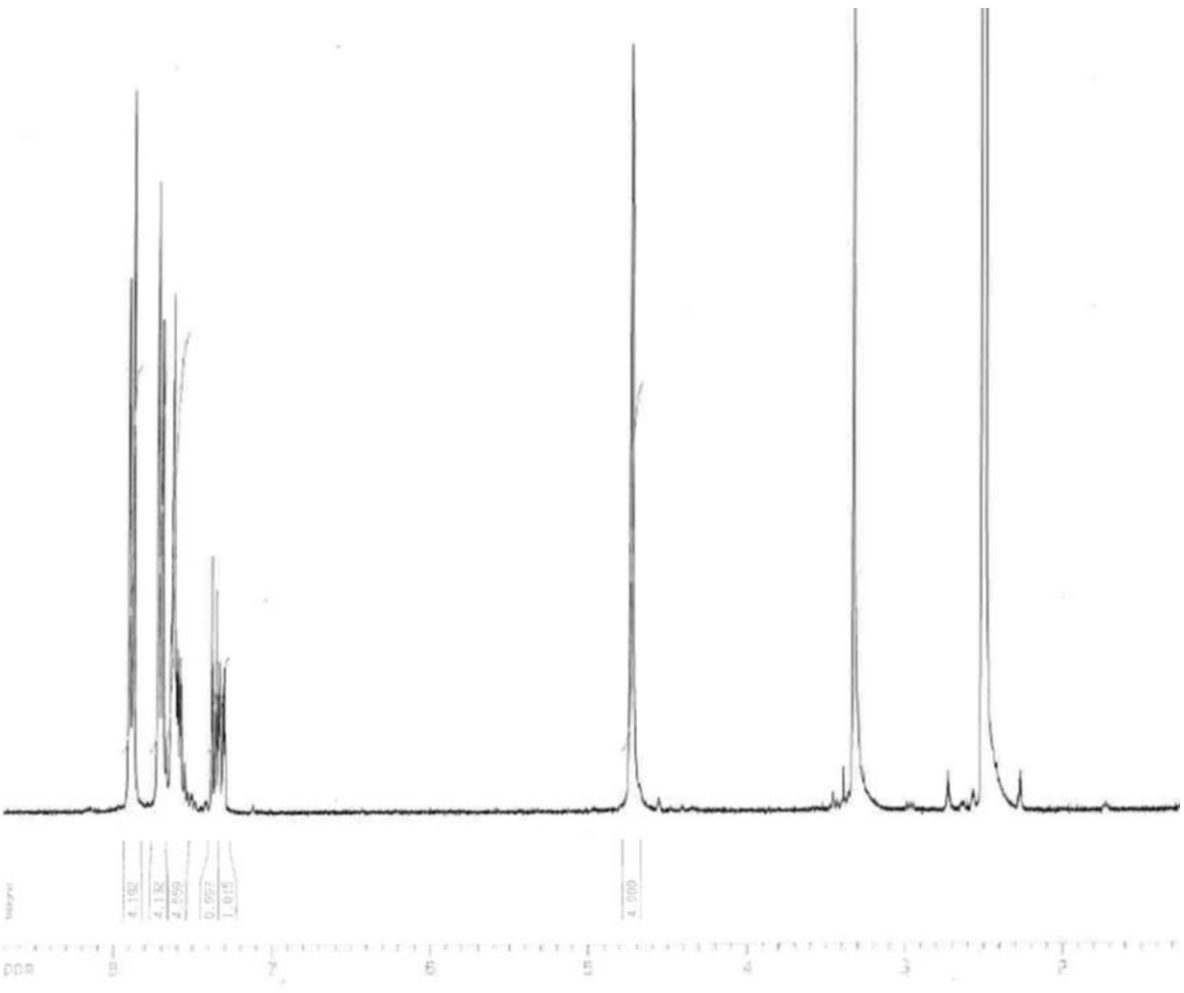

Figure S4. ^{1}H NMR spectrum of **3d**.

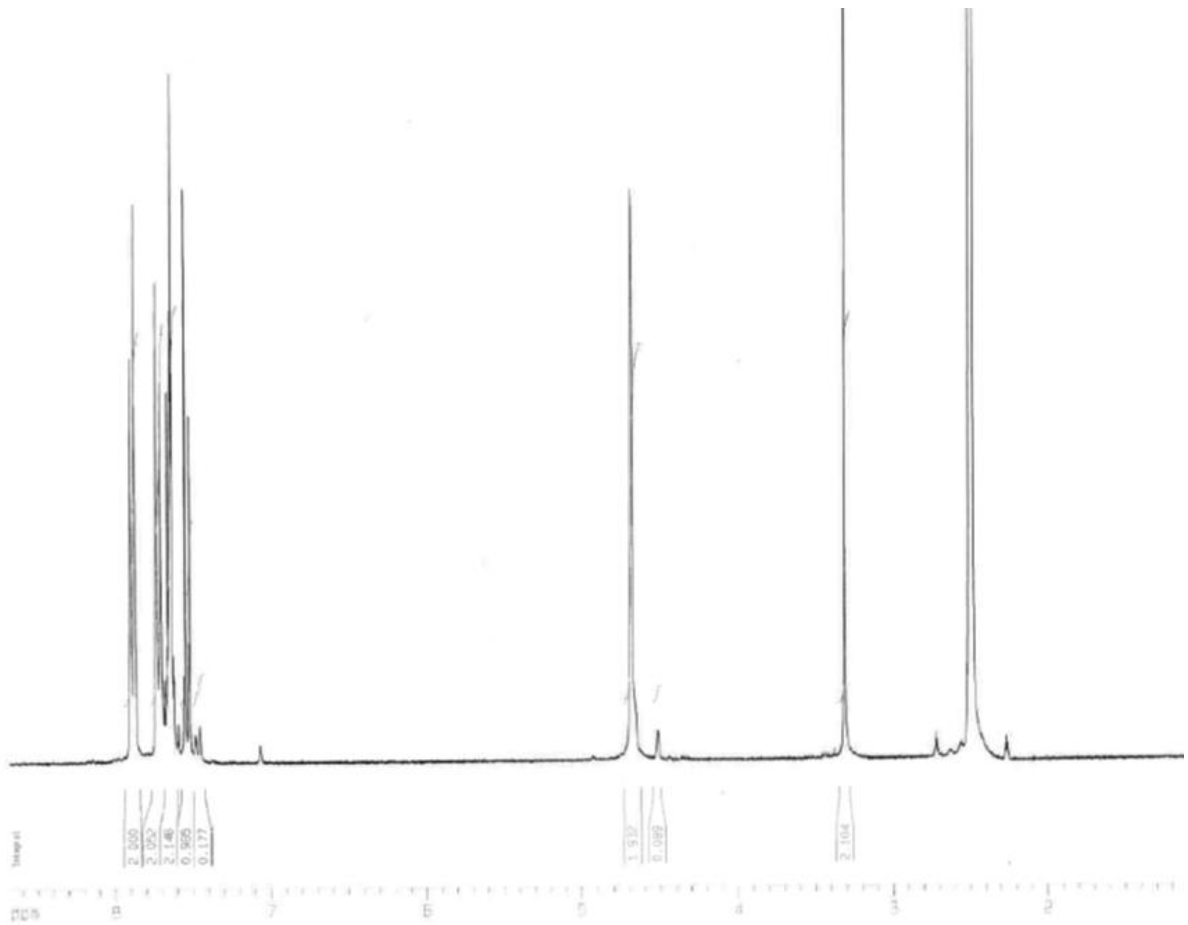

Figure S5. ^{1}H NMR spectrum of **3e**.

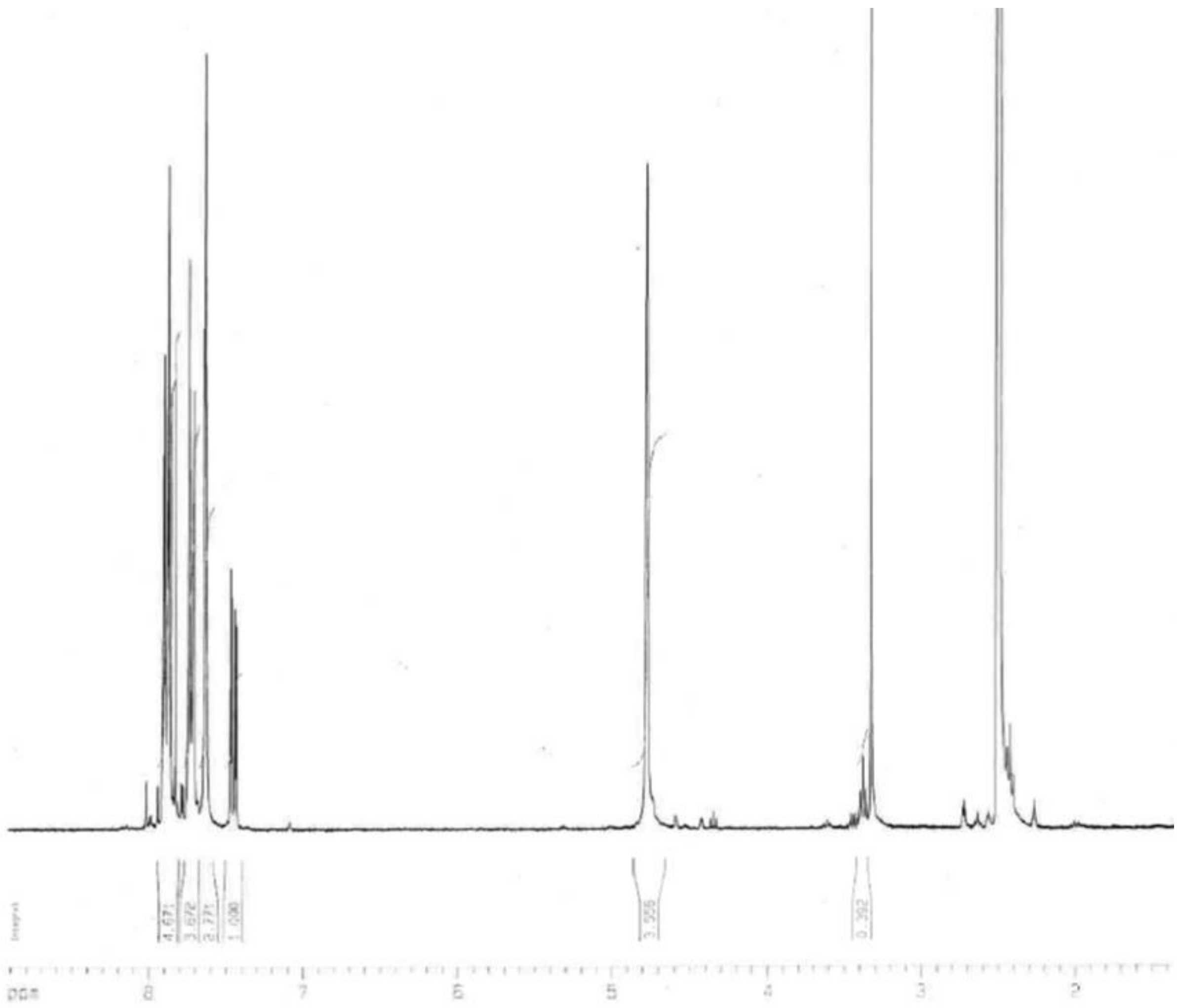

Figure S6. ^{1}H NMR spectrum of **3f**.

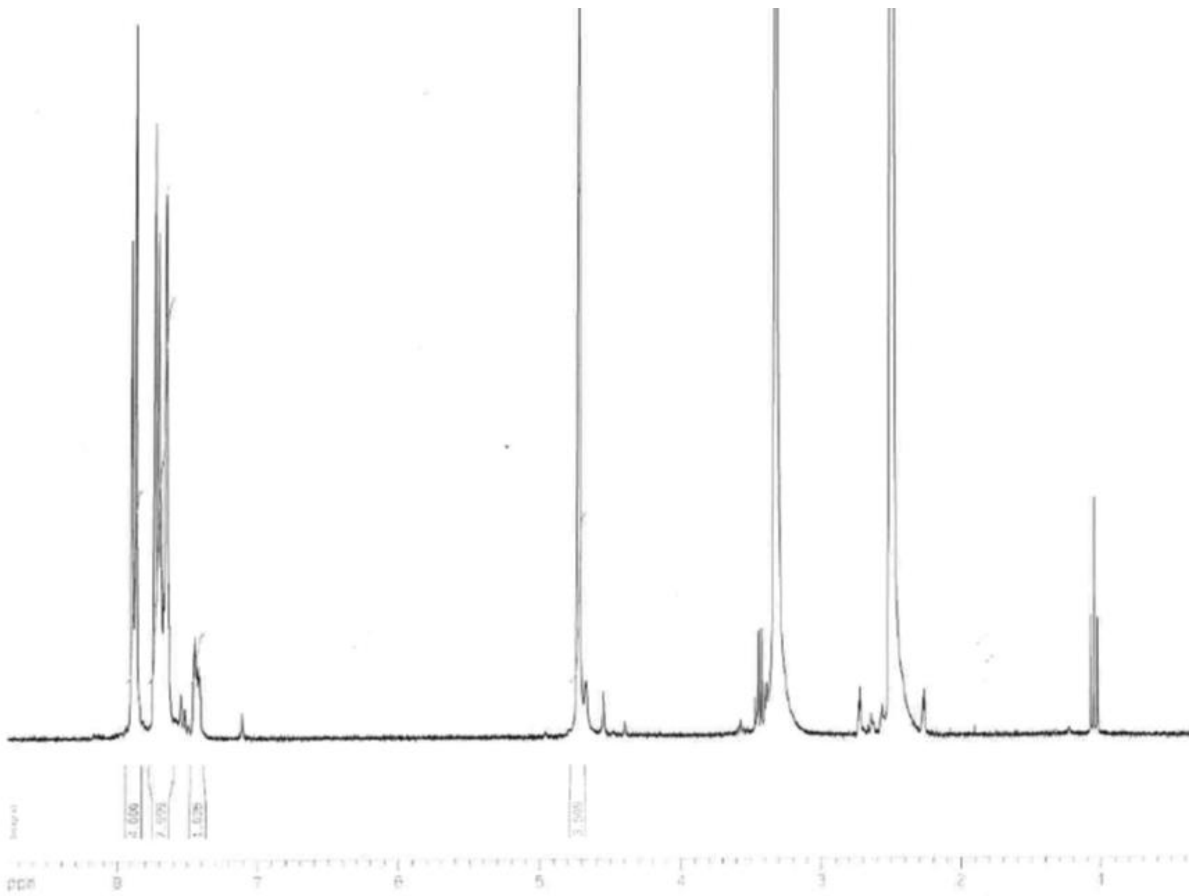

Figure S7. ^{1}H NMR spectrum of **3g**.

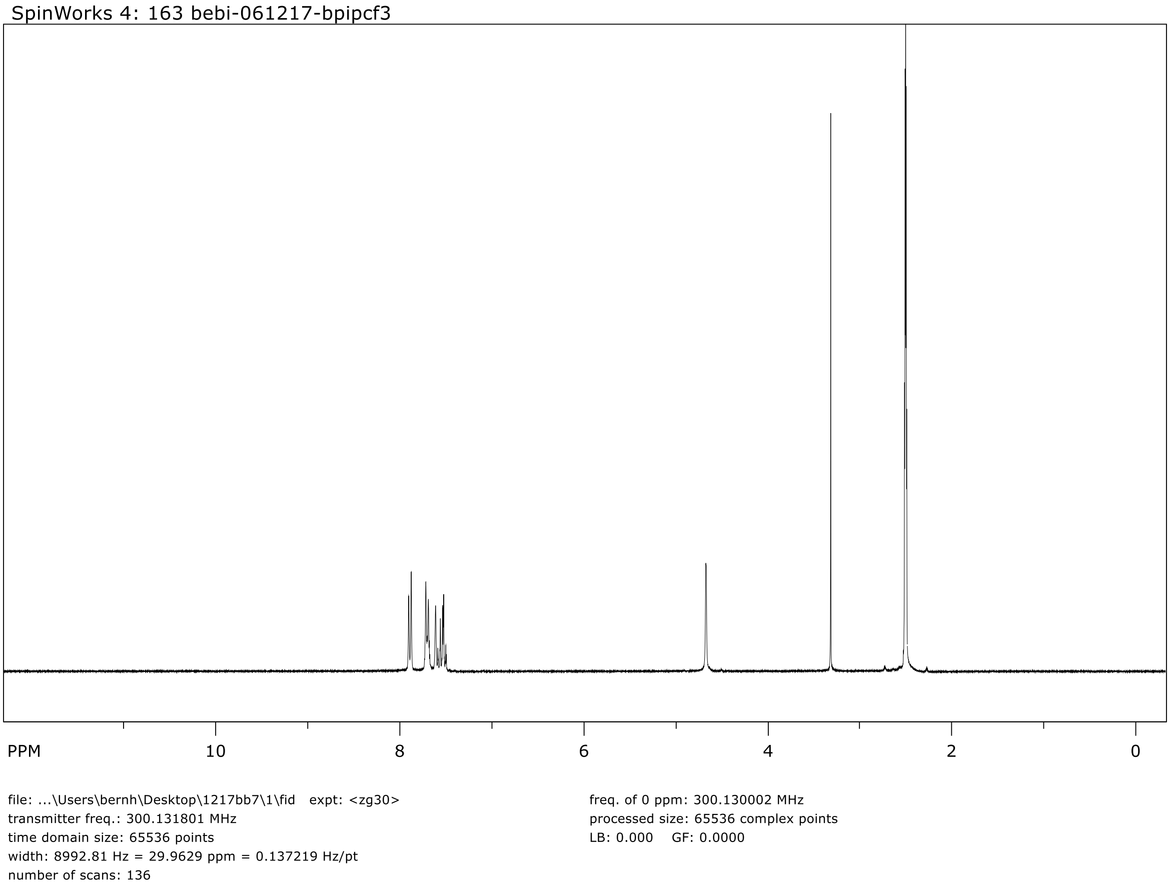

Figure S8. ^{1}H NMR spectrum of **3h**.

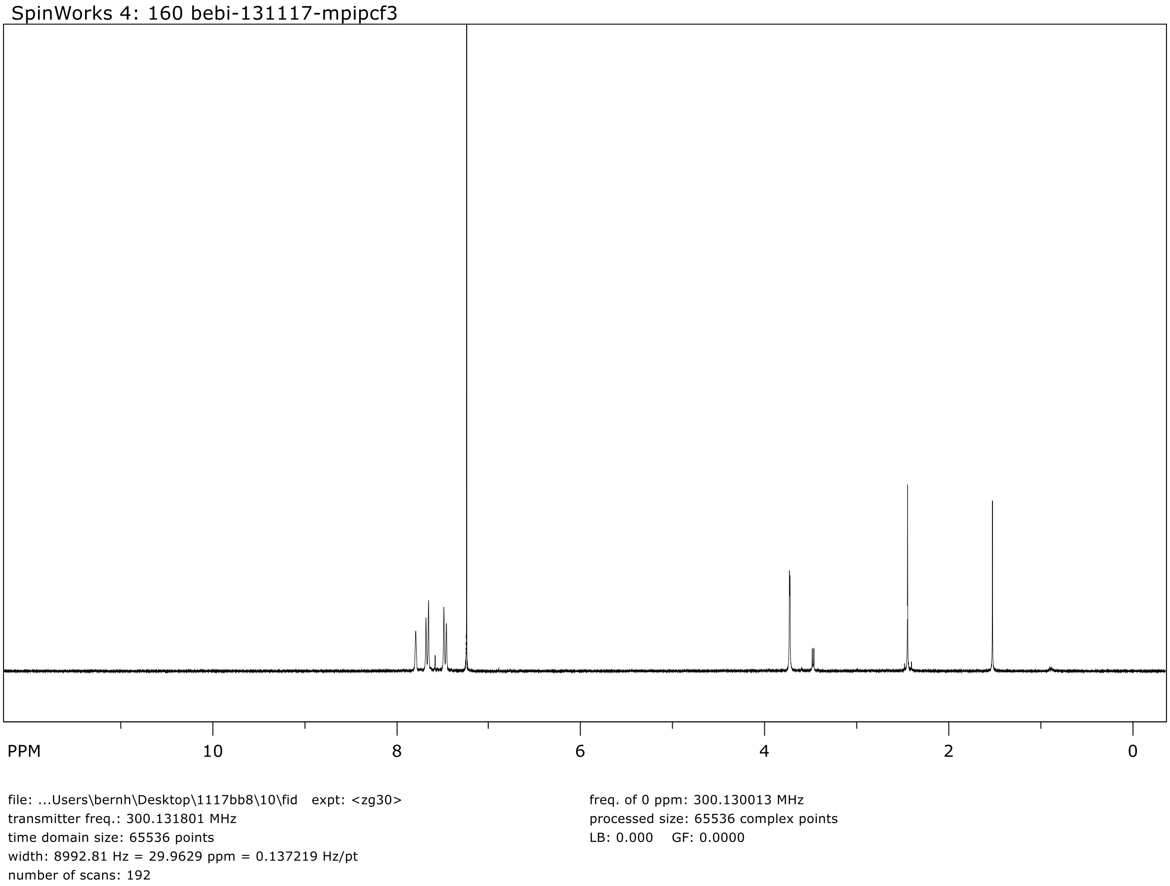

Figure S9. ^{1}H NMR spectrum of **3i**.

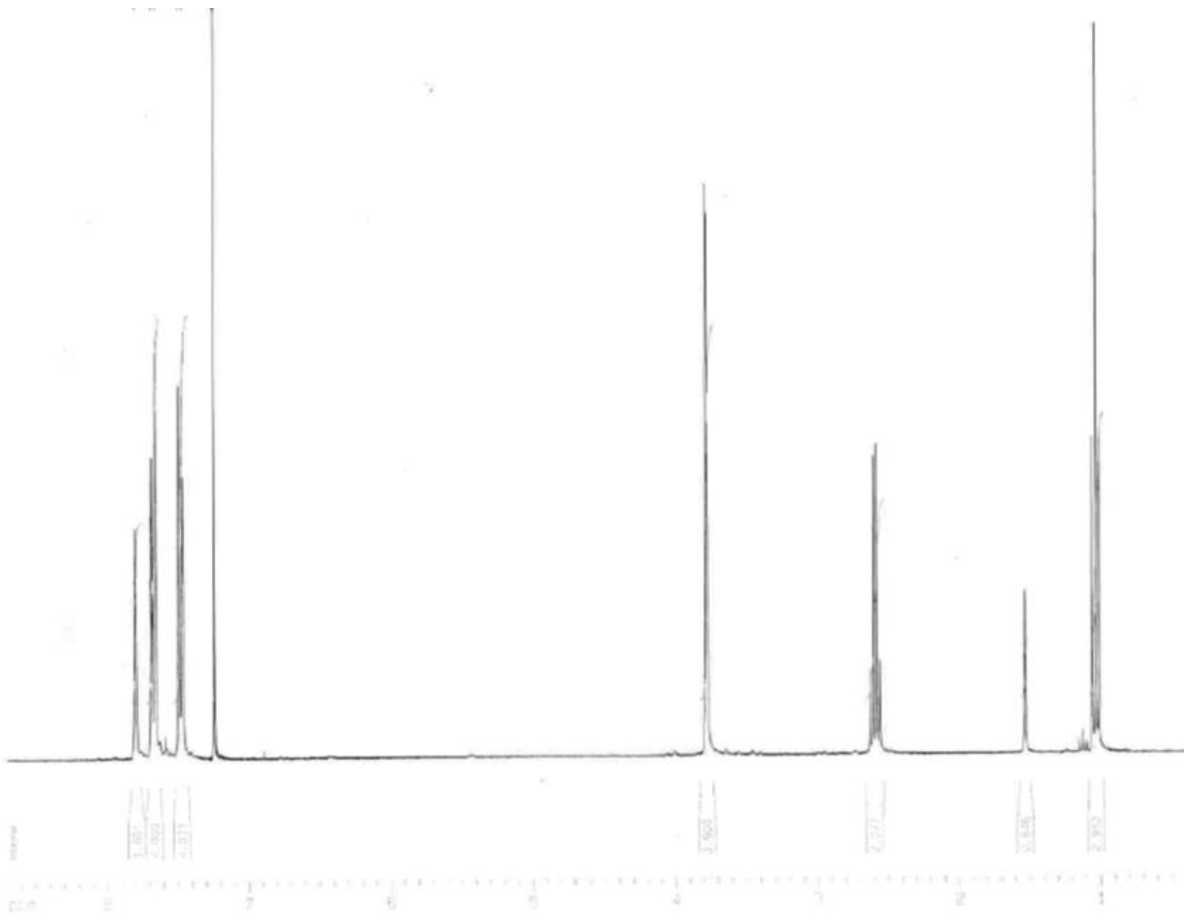

Figure S10. ^{1}H NMR spectrum of **3j**.

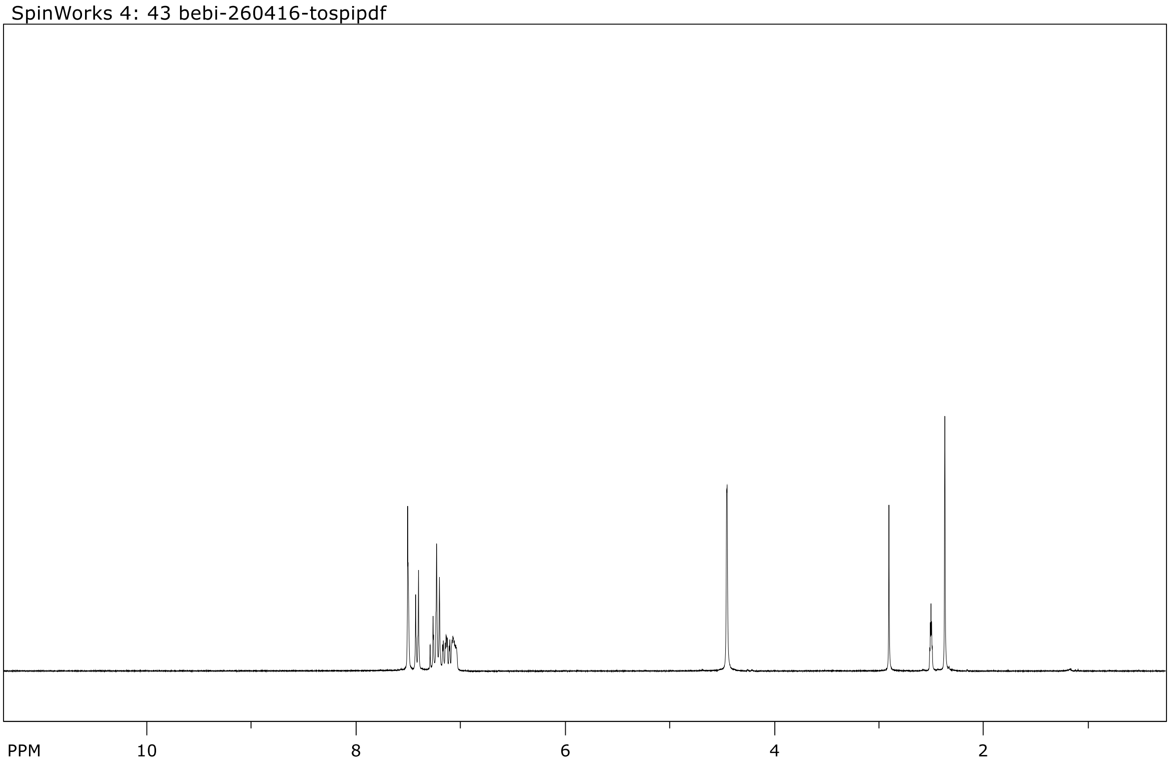

file: ...ers\bernh\Desktop\0416bb16\100\fid expt: <zg30>
transmitter freq.: 300.131801 MHz
time domain size: 65536 points
width: 8992.81 Hz = 29.9629 ppm = 0.137219 Hz/pt
number of scans: 100

freq. of 0 ppm: 300.131429 MHz
processed size: 65536 complex points
LB: 0.000 GF: 0.0000

Figure S11. ^{1}H NMR spectrum of **4a**.

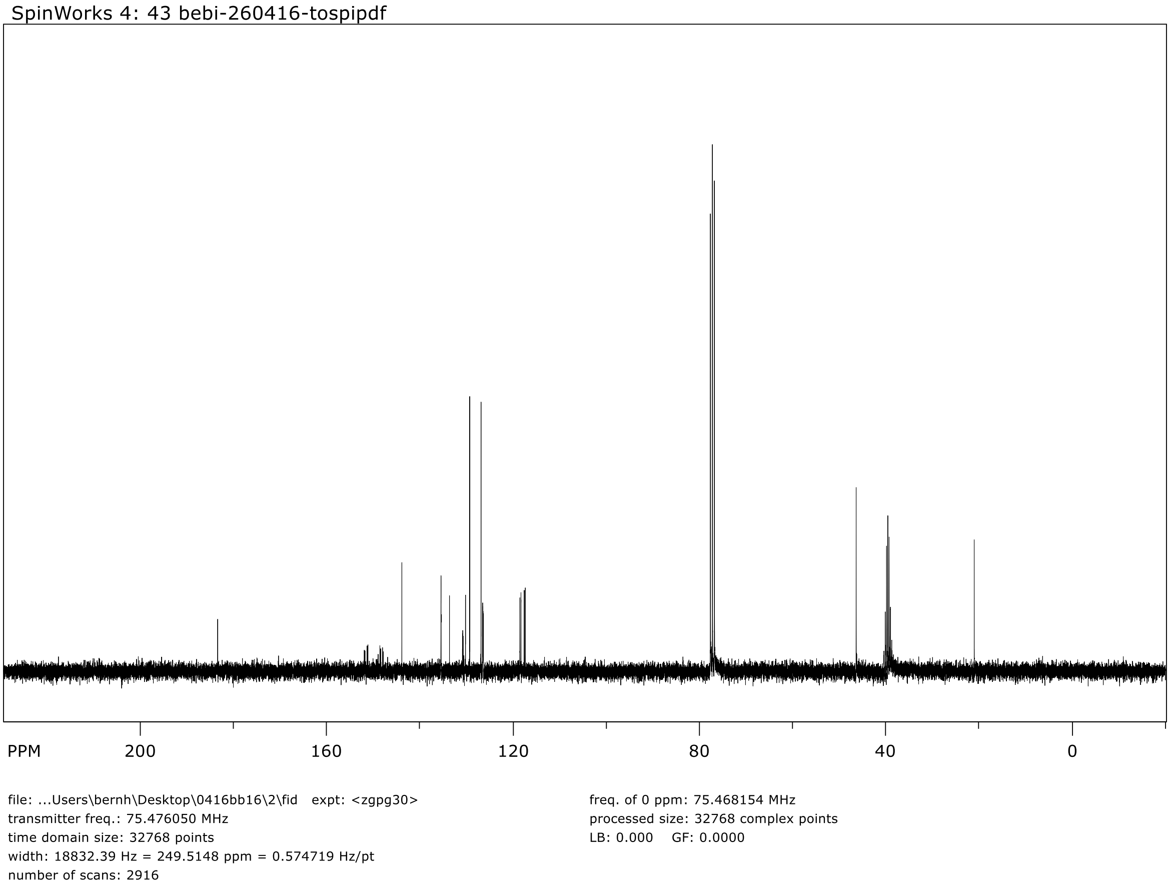

Figure S12. ^{13}C NMR spectrum of **4a**.

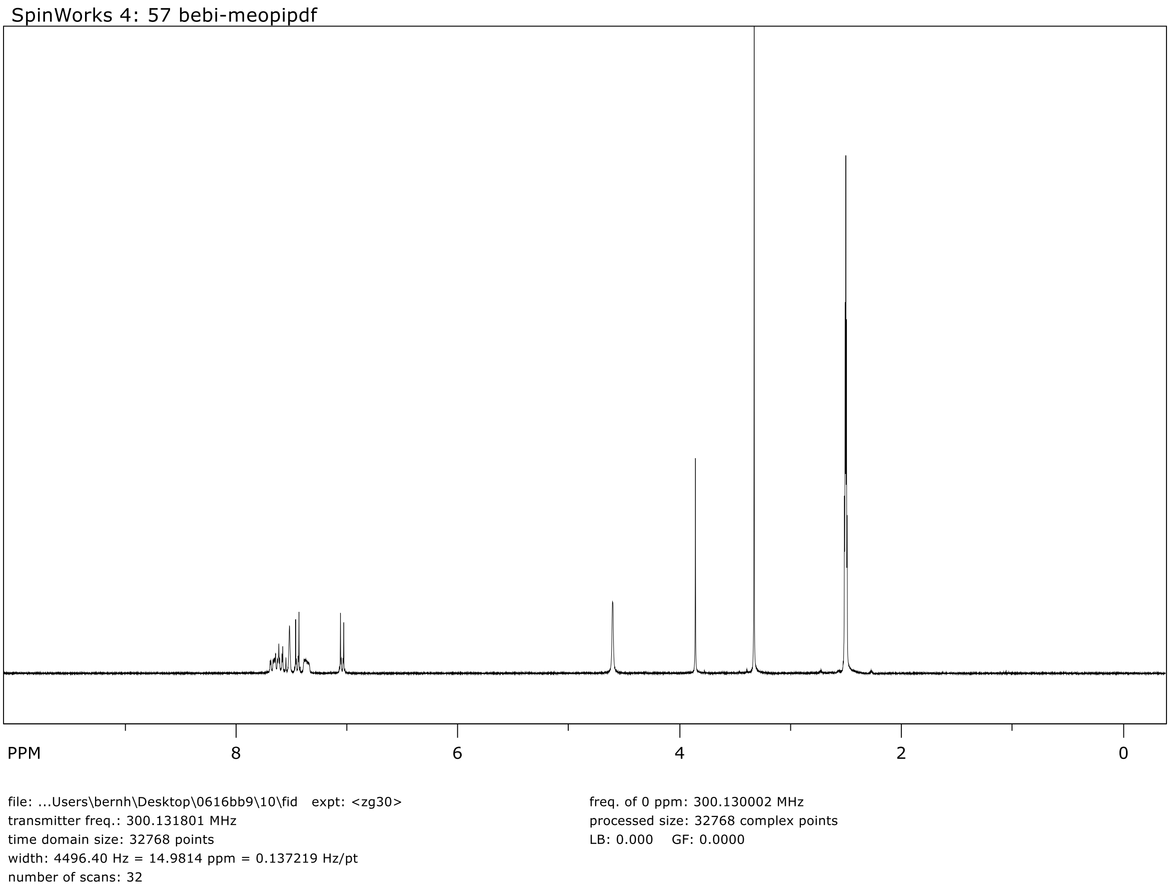

Figure S13. ^{1}H NMR spectrum of **4b**.

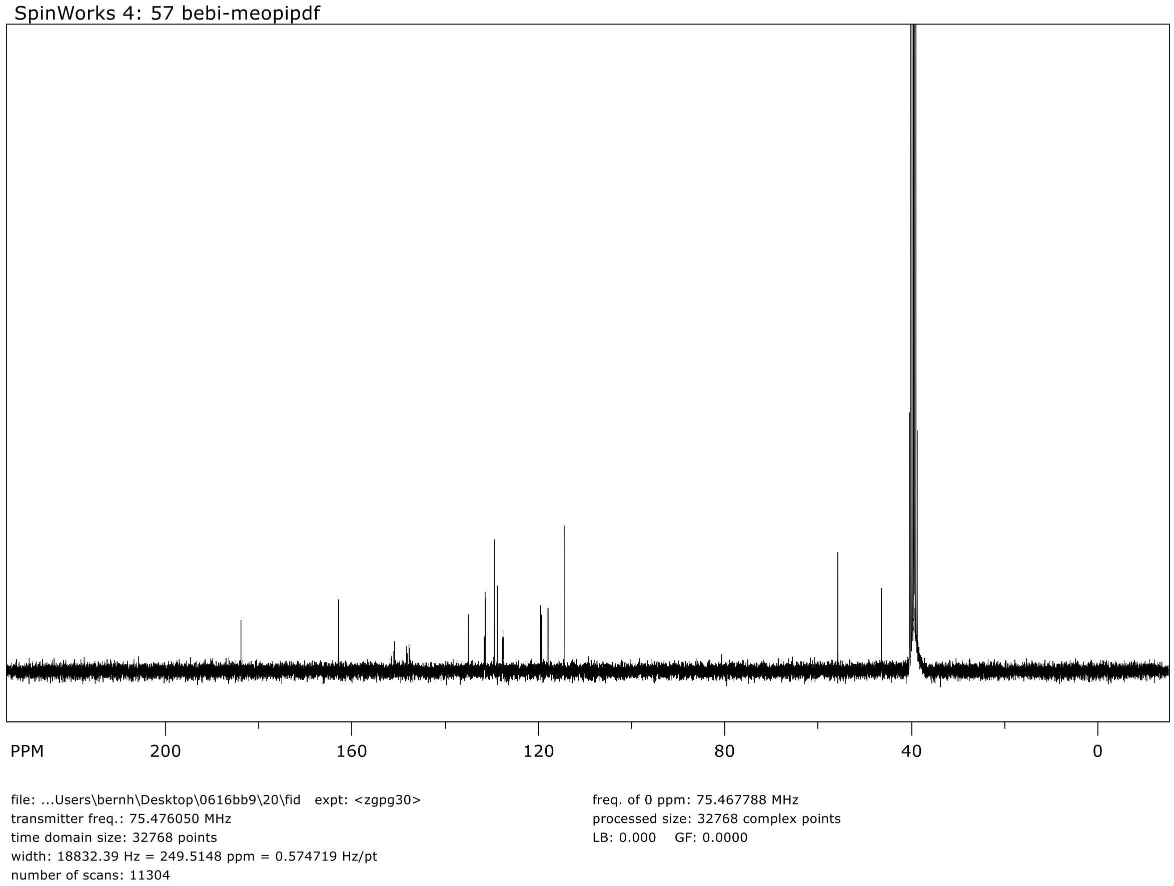

Figure S14. ^{13}C NMR spectrum of **4b**.

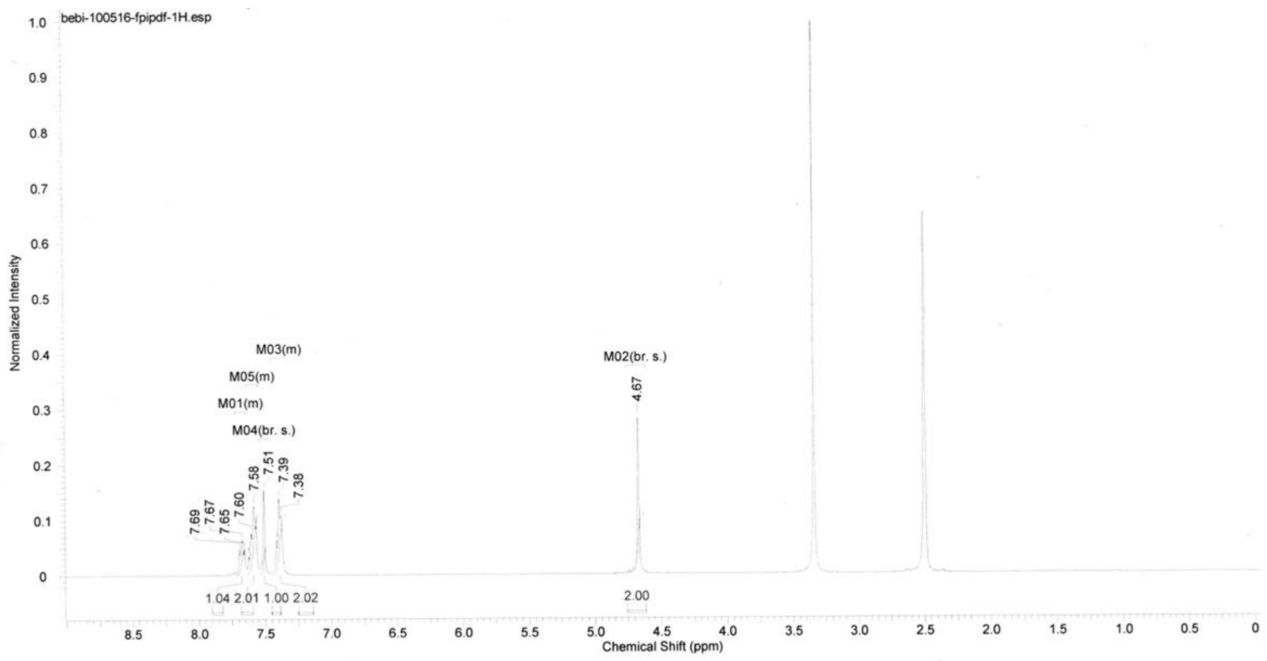

Figure S15. ^{1}H NMR spectrum of **4c**.

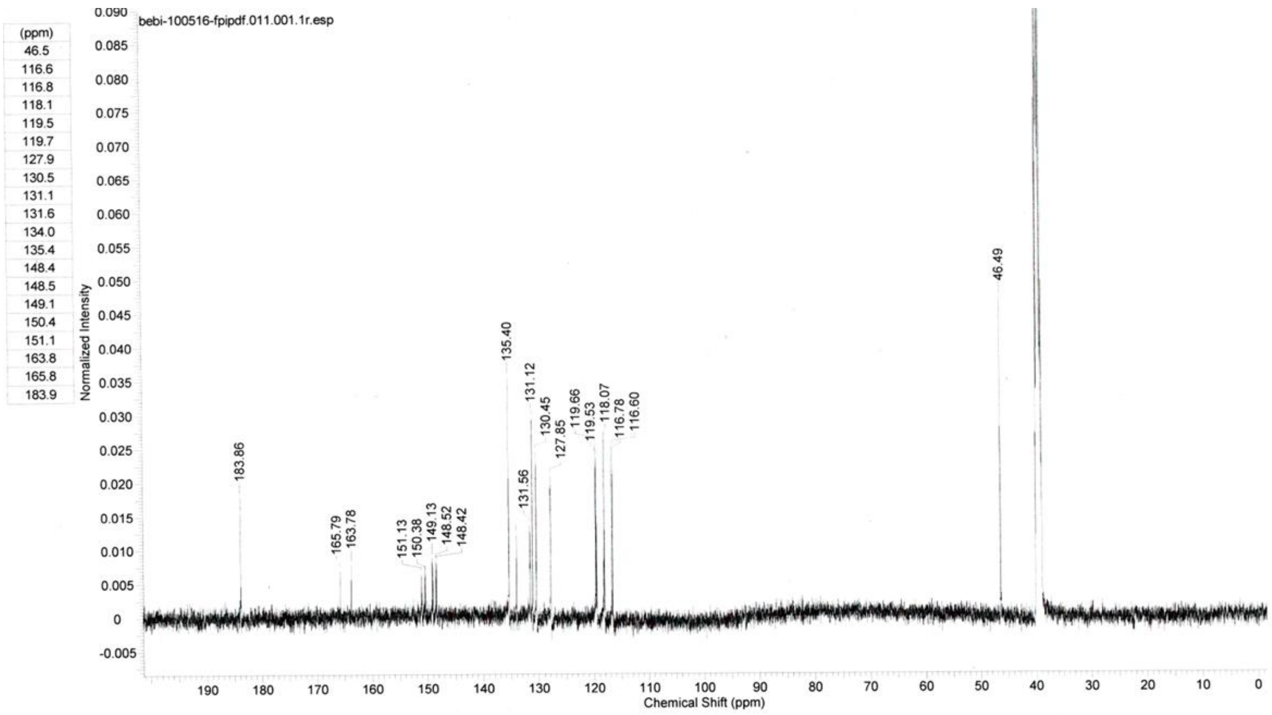

Figure S16. ^{13}C NMR spectrum of **4c**.

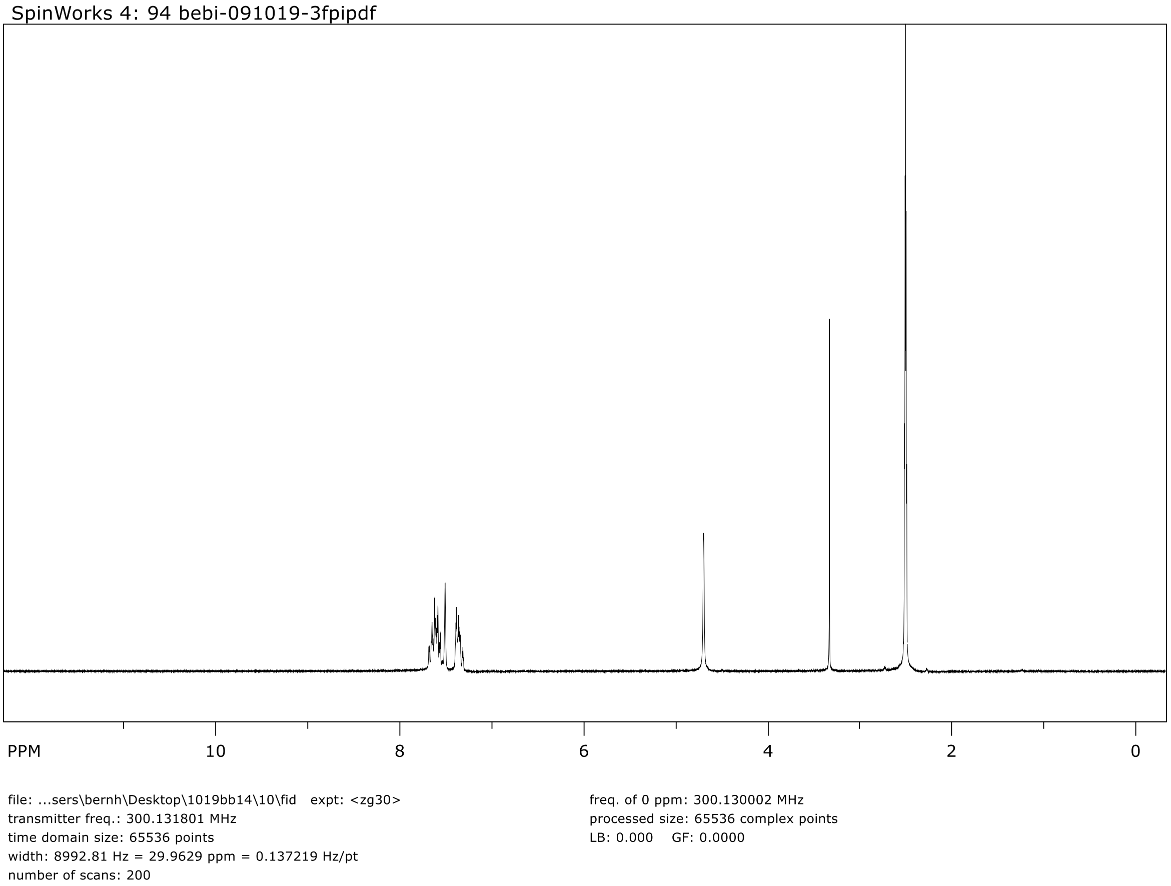

Figure S17. ^{1}H NMR spectrum of **4d**.

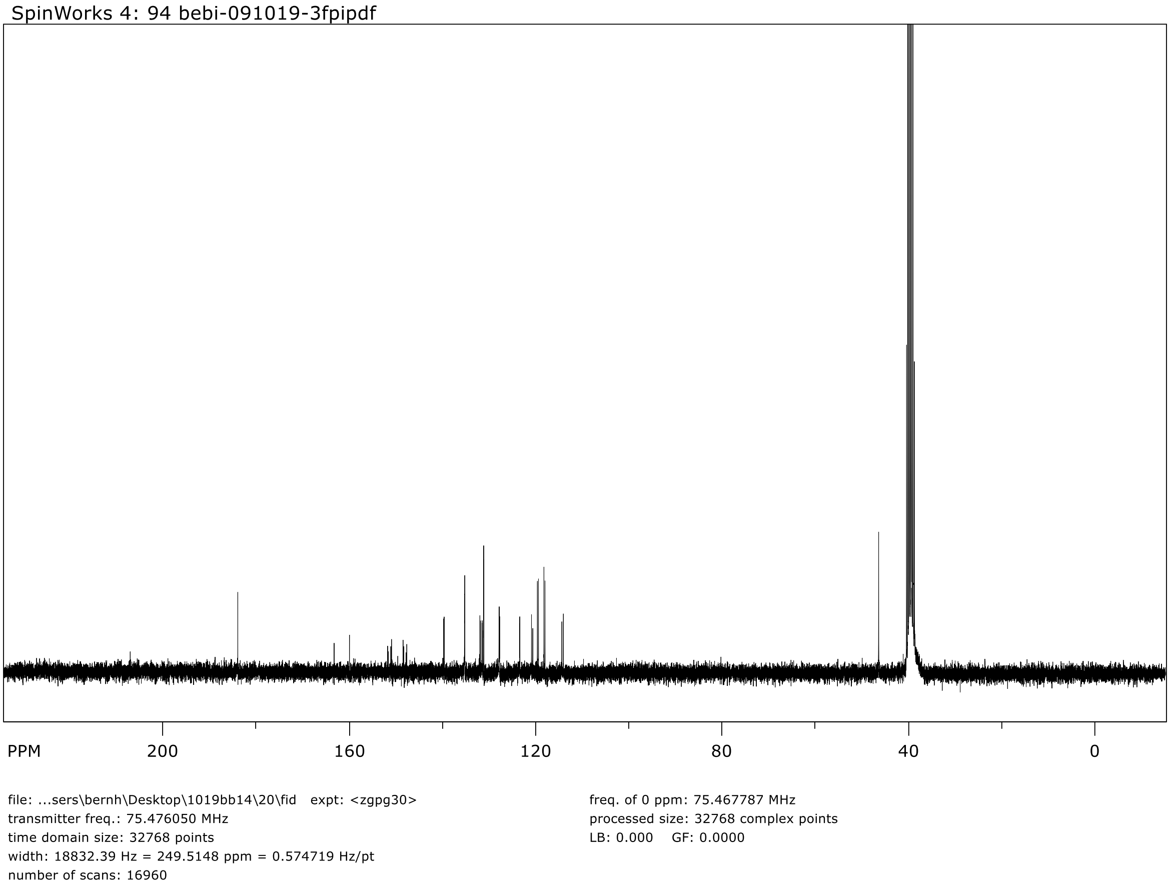

Figure S18. ^{13}C NMR spectrum of **4d**.

SpinWorks 4: 51 bebi-140616-clpipdf

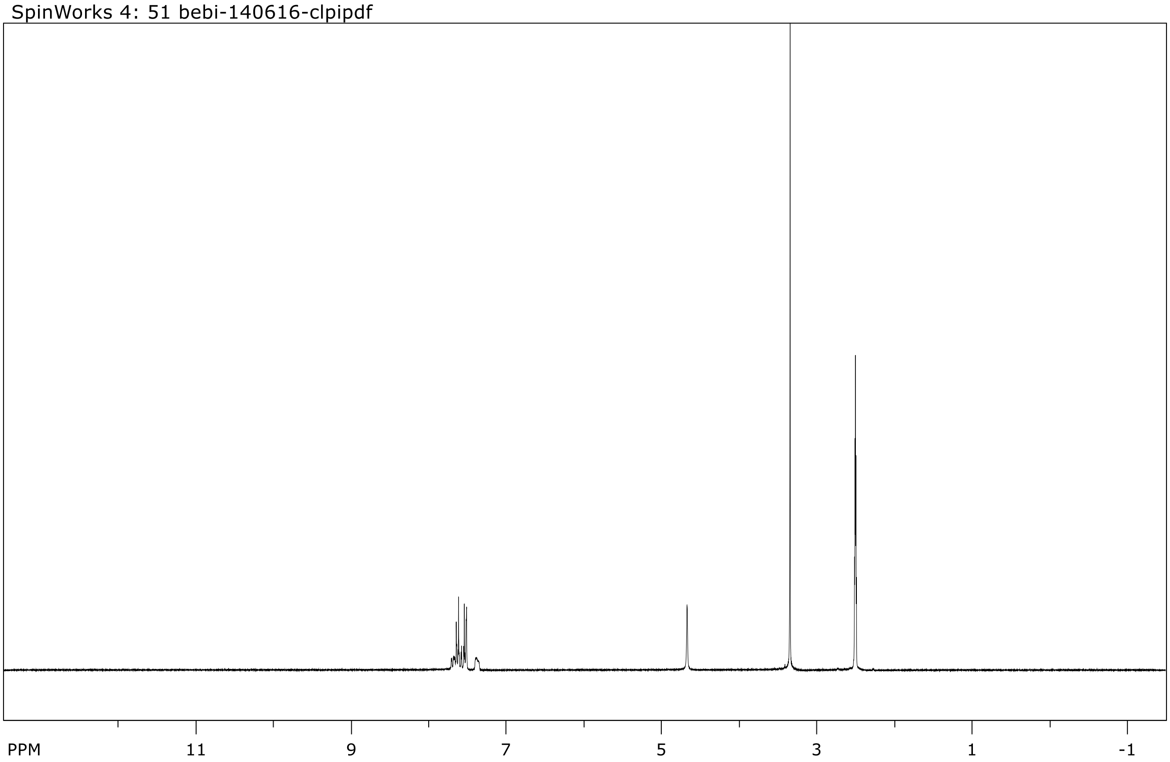

file: ...Users\bernh\Desktop\0616bb5\10\fid expt: <zg30>
transmitter freq.: 300.131801 MHz
time domain size: 32768 points
width: 4496.40 Hz = 14.9814 ppm = 0.137219 Hz/pt
number of scans: 44

freq. of 0 ppm: 300.130002 MHz
processed size: 32768 complex points
LB: 0.000 GF: 0.0000

Figure S19. ^{1}H NMR spectrum of **4e**.

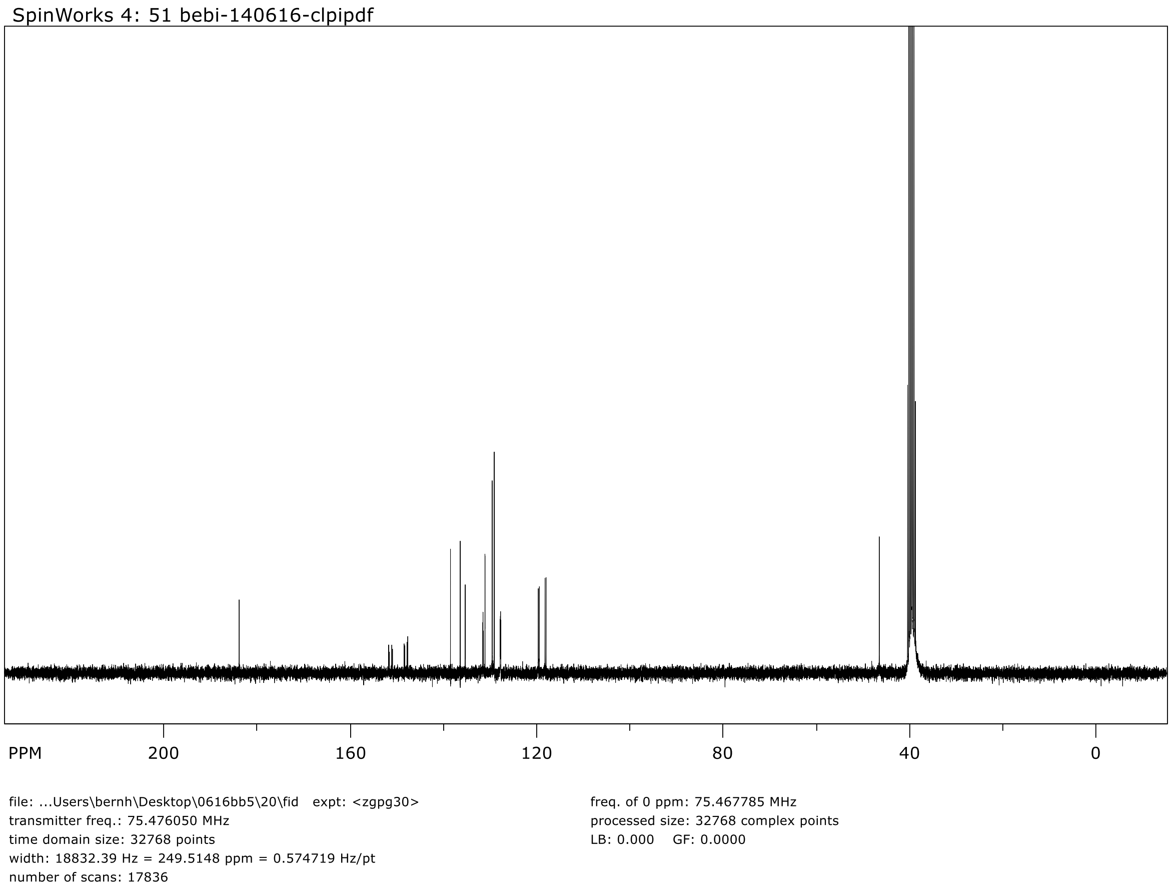

Figure S20. ^{13}C NMR spectrum of **4e**.

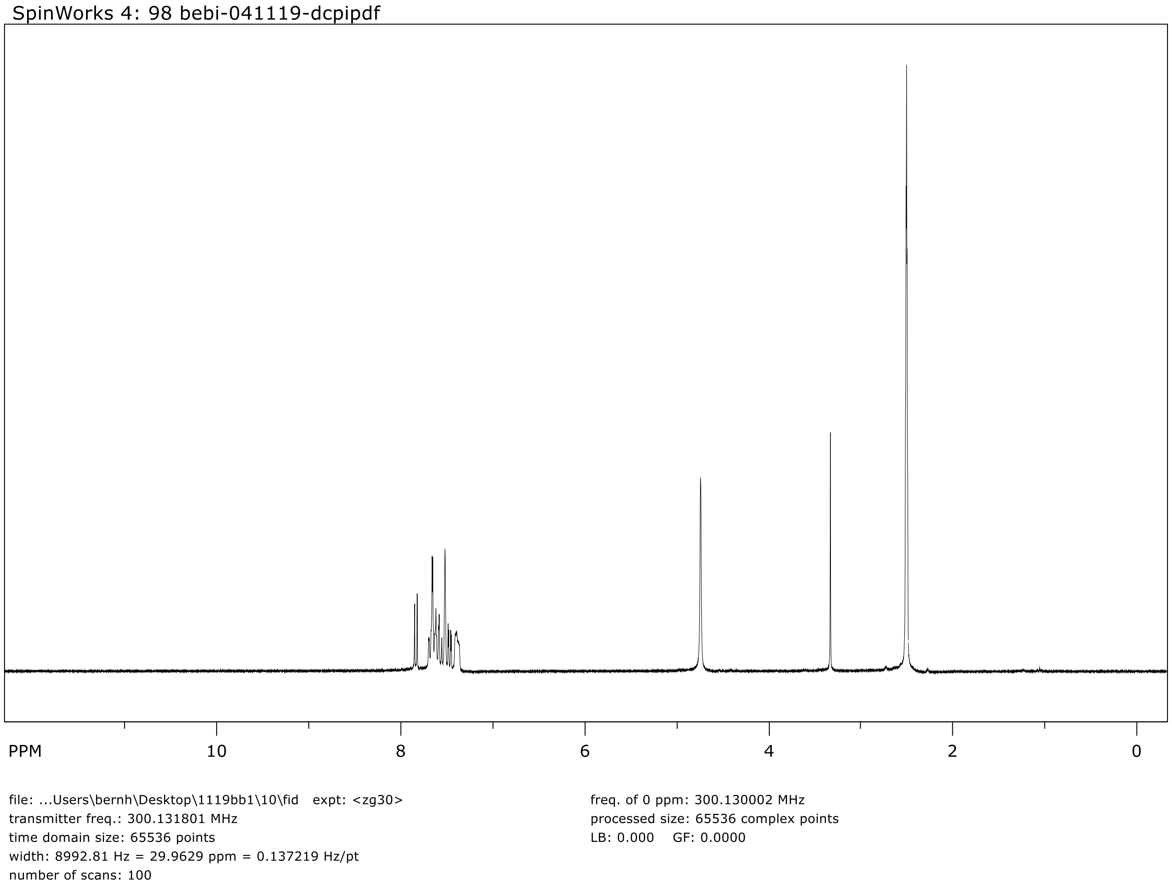

Figure S21. ^{1}H NMR spectrum of **4f**.

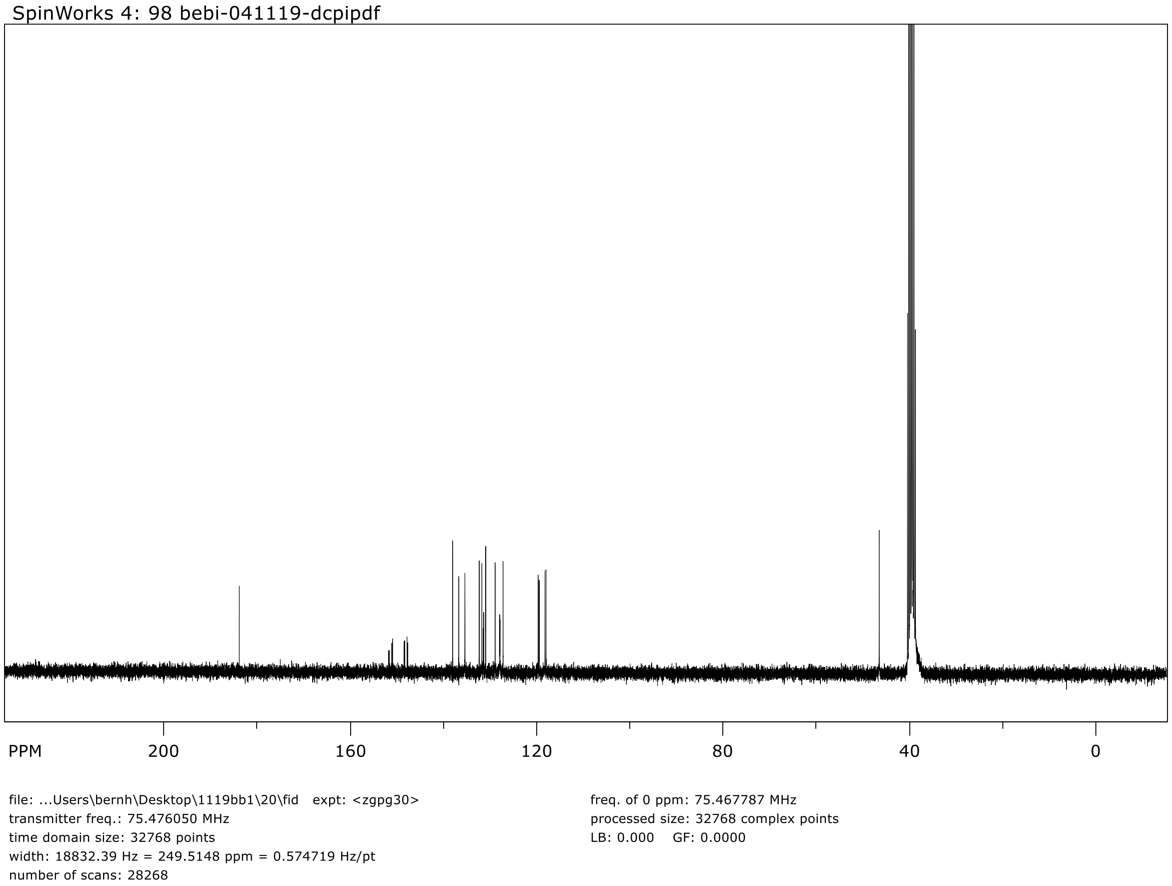

Figure S22. ^{13}C NMR spectrum of **4f**.

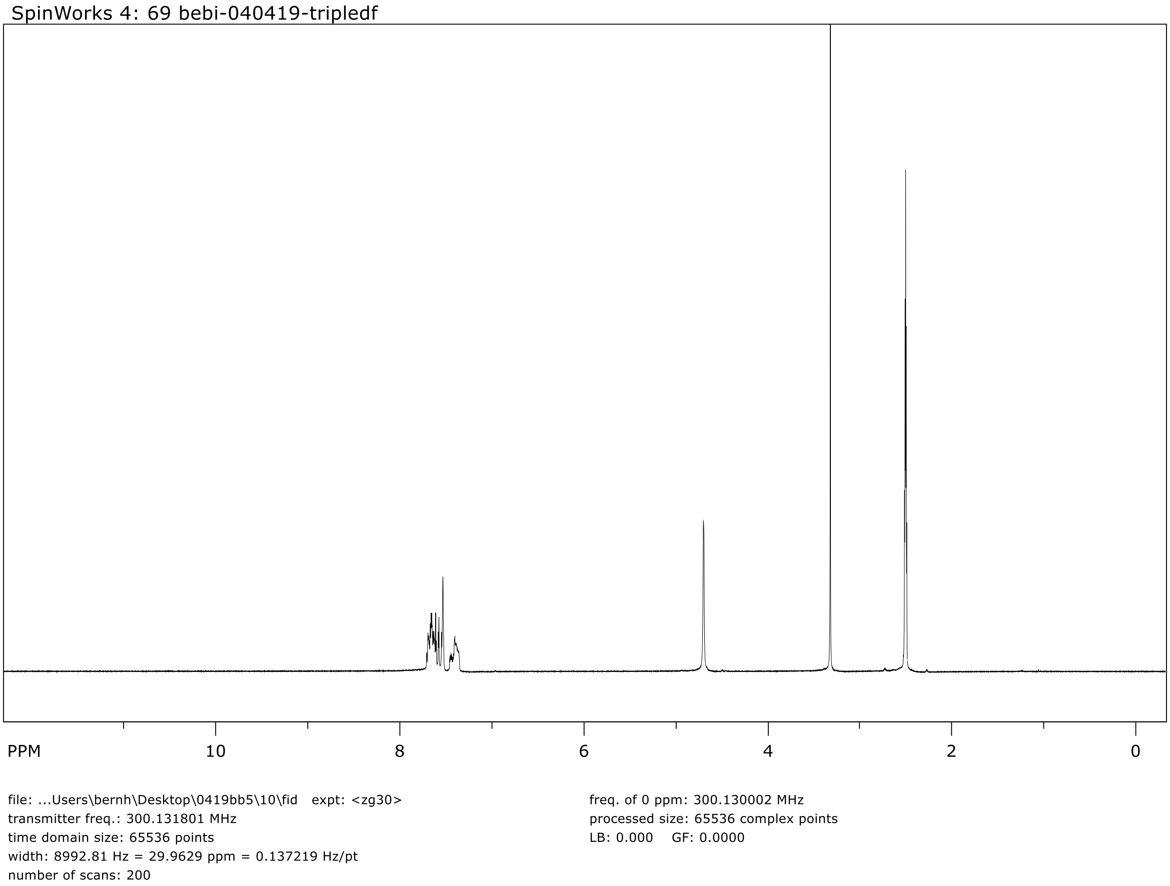

Figure S23. ^{1}H NMR spectrum of **4g**.

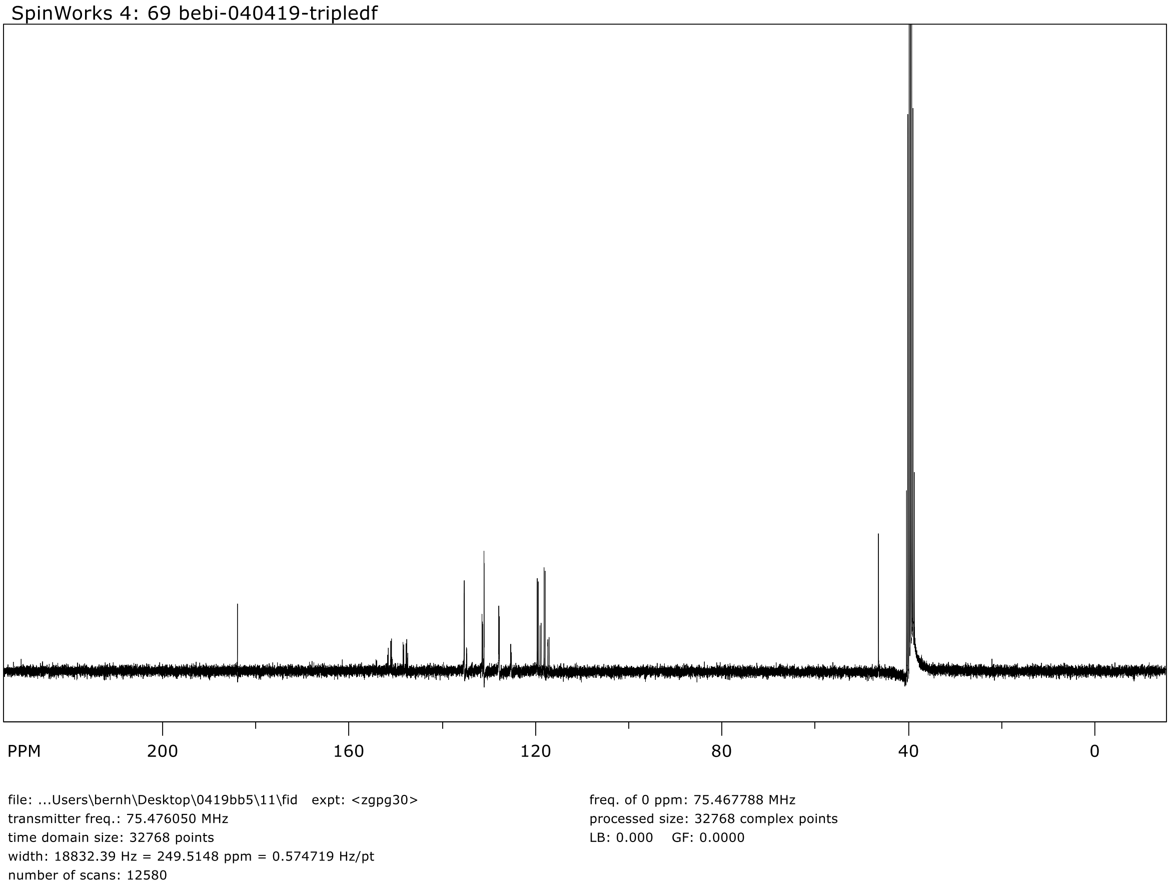

Figure S24. ^{13}C NMR spectrum of **4g**.

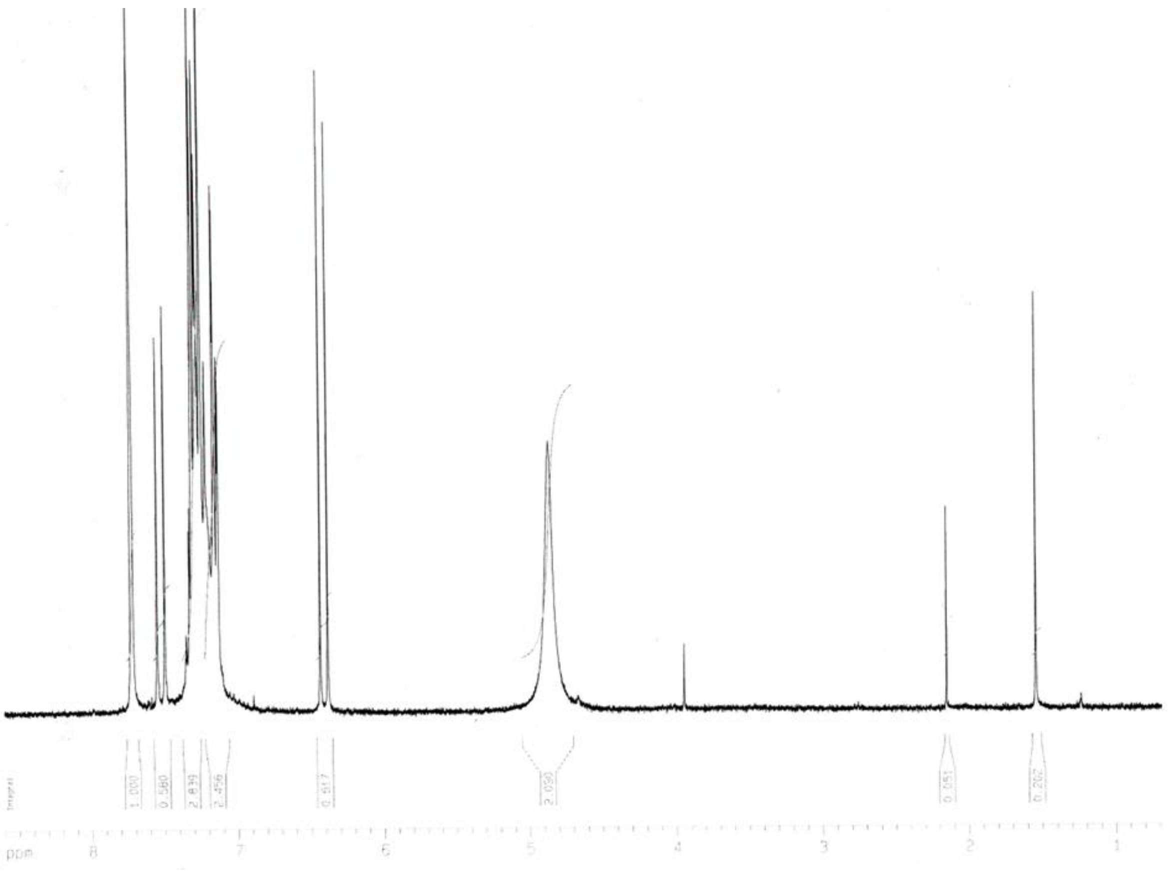

Figure S25. ^{1}H NMR spectrum of **4h**.

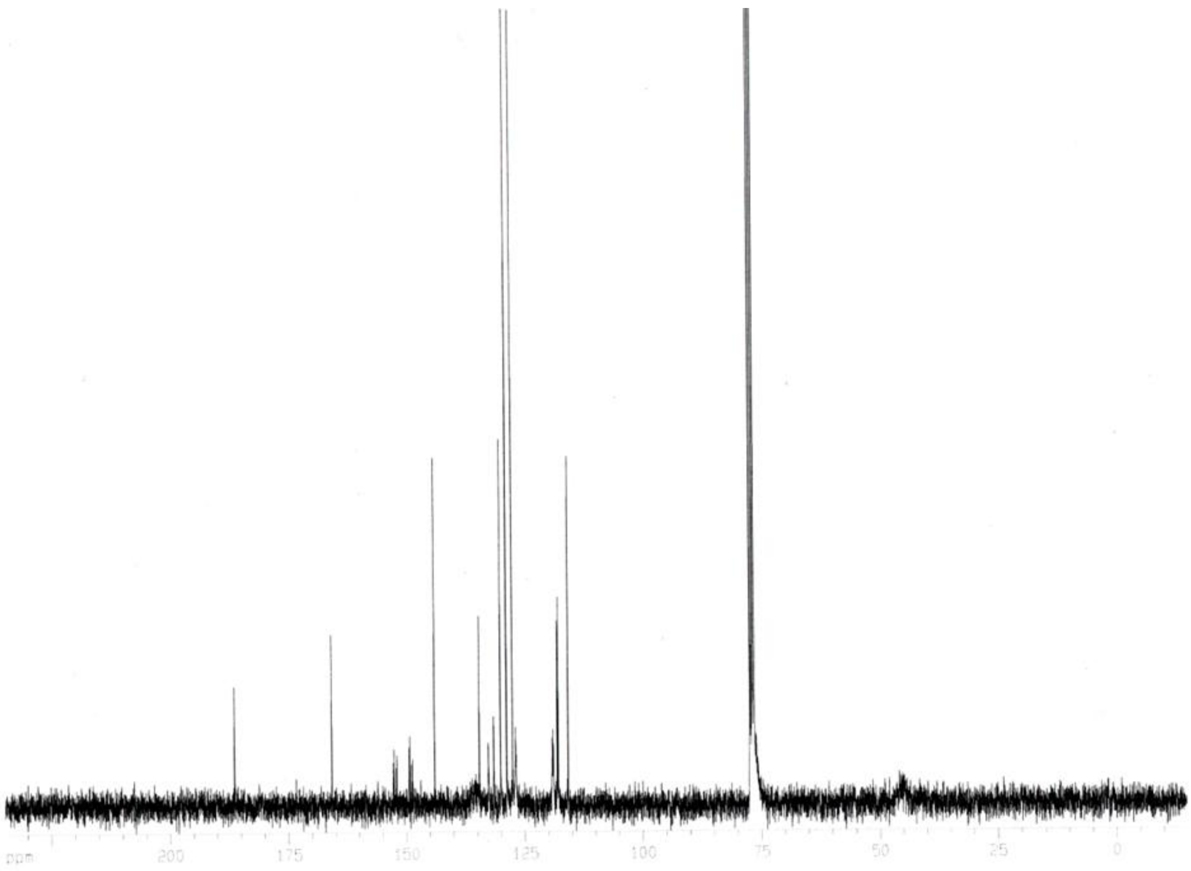

Figure S26. ^{1}H NMR spectrum of **4h**.

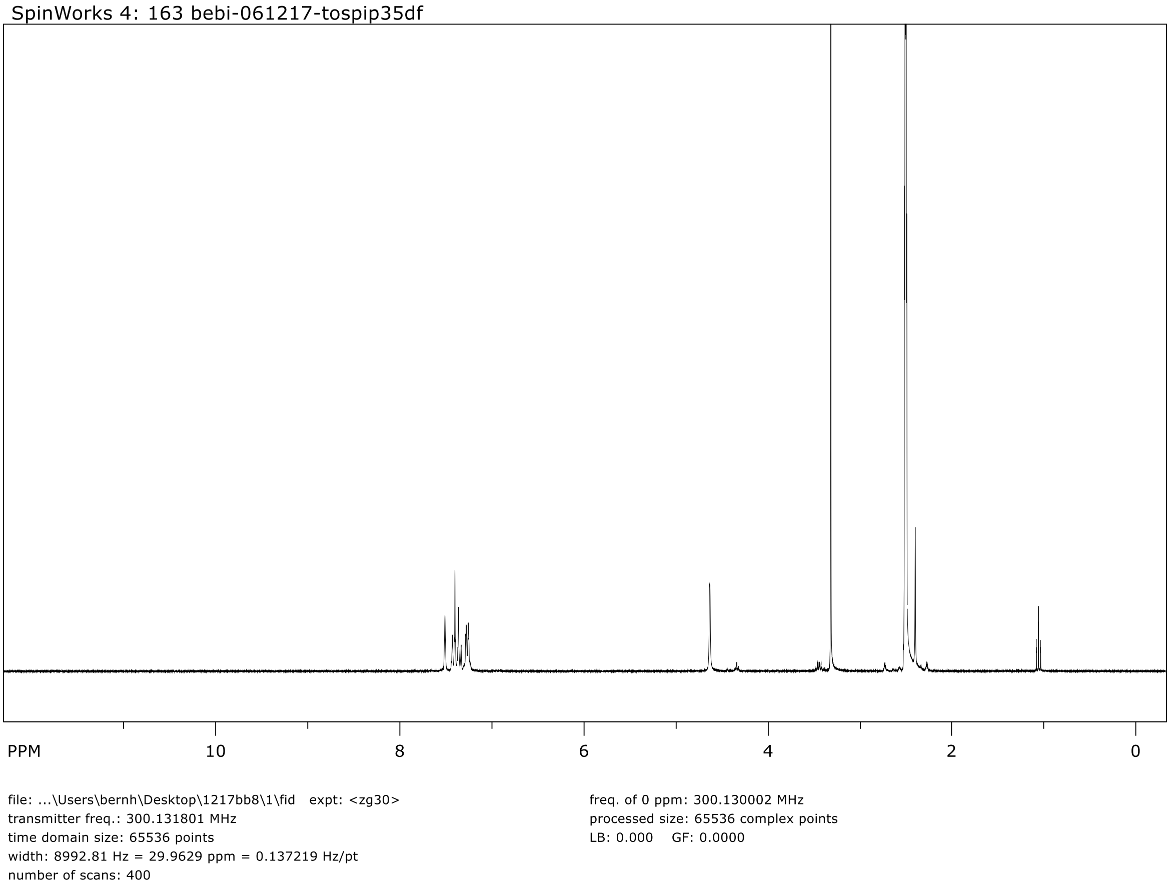

Figure S27. ^{1}H NMR spectrum of **1a**.

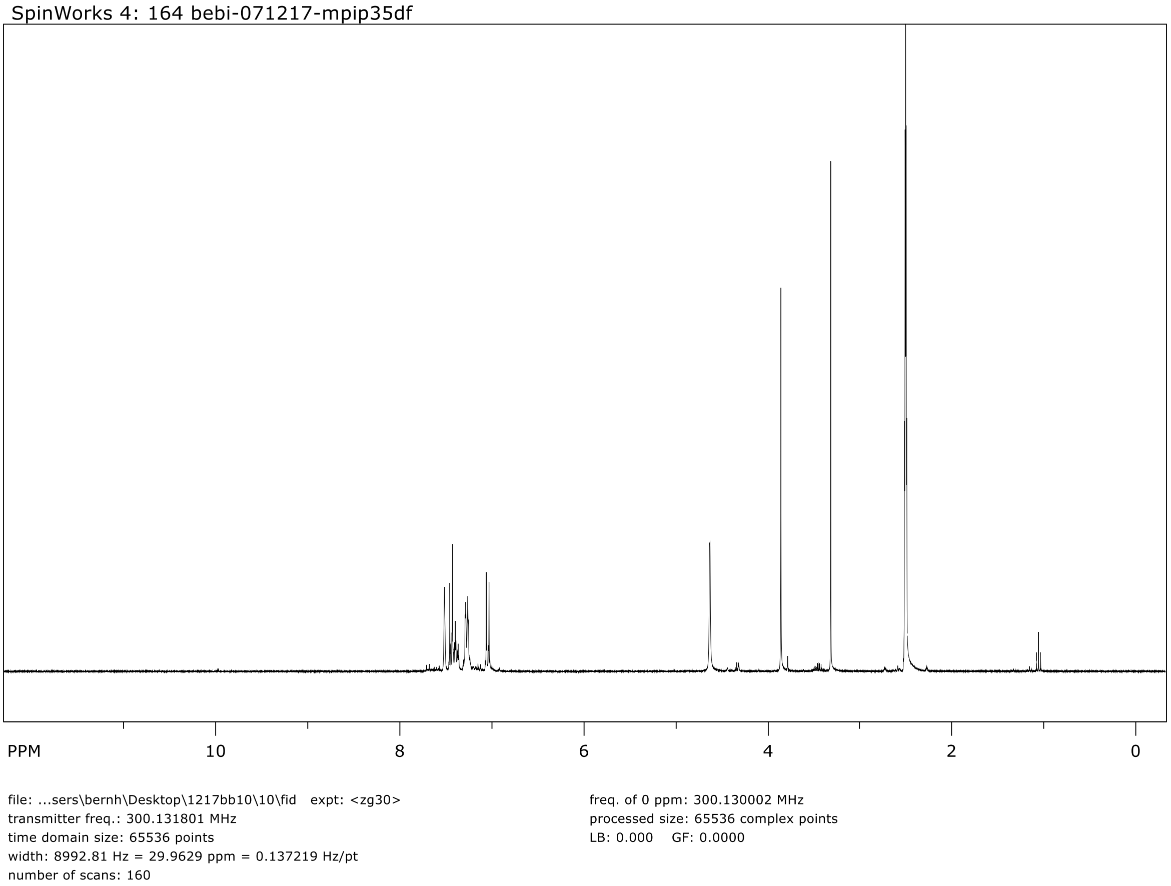

Figure S28. ^{1}H NMR spectrum of **5b**.

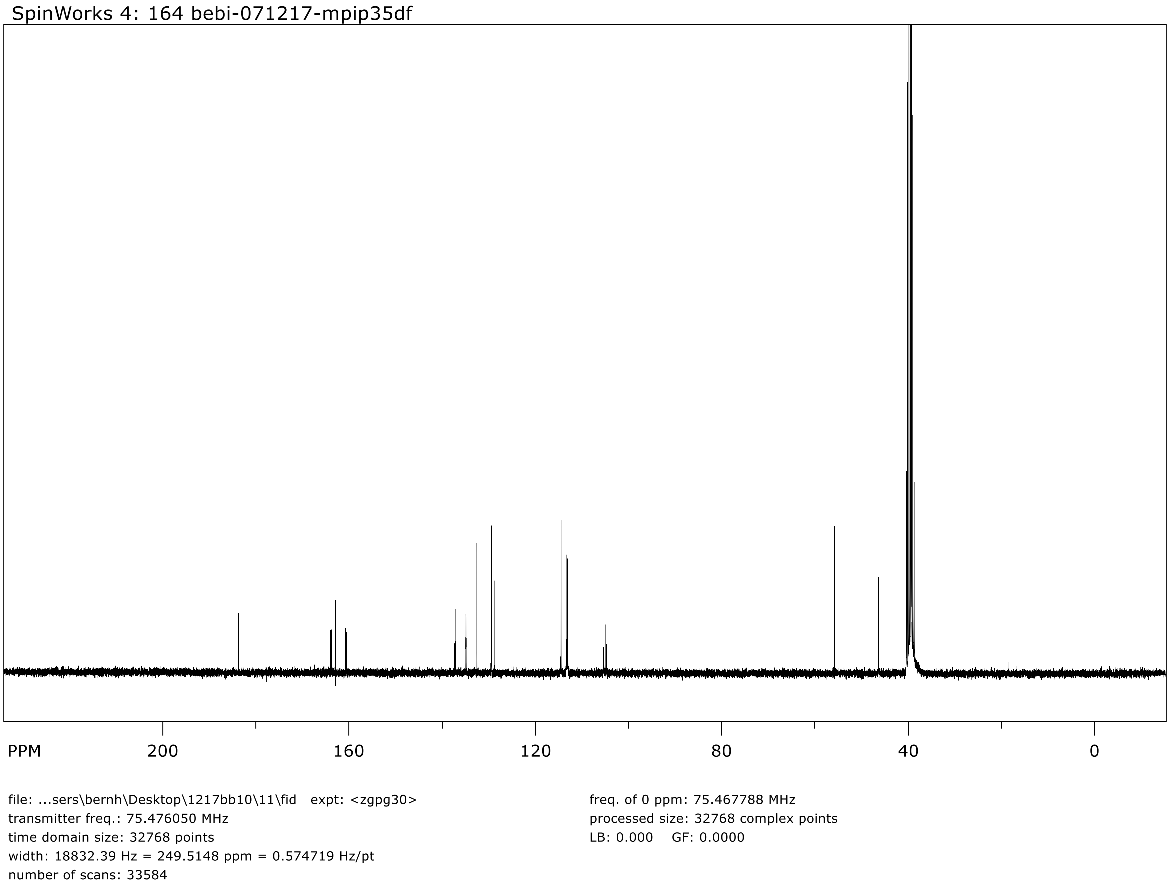

Figure S29. ^{13}C NMR spectrum of **5b**.

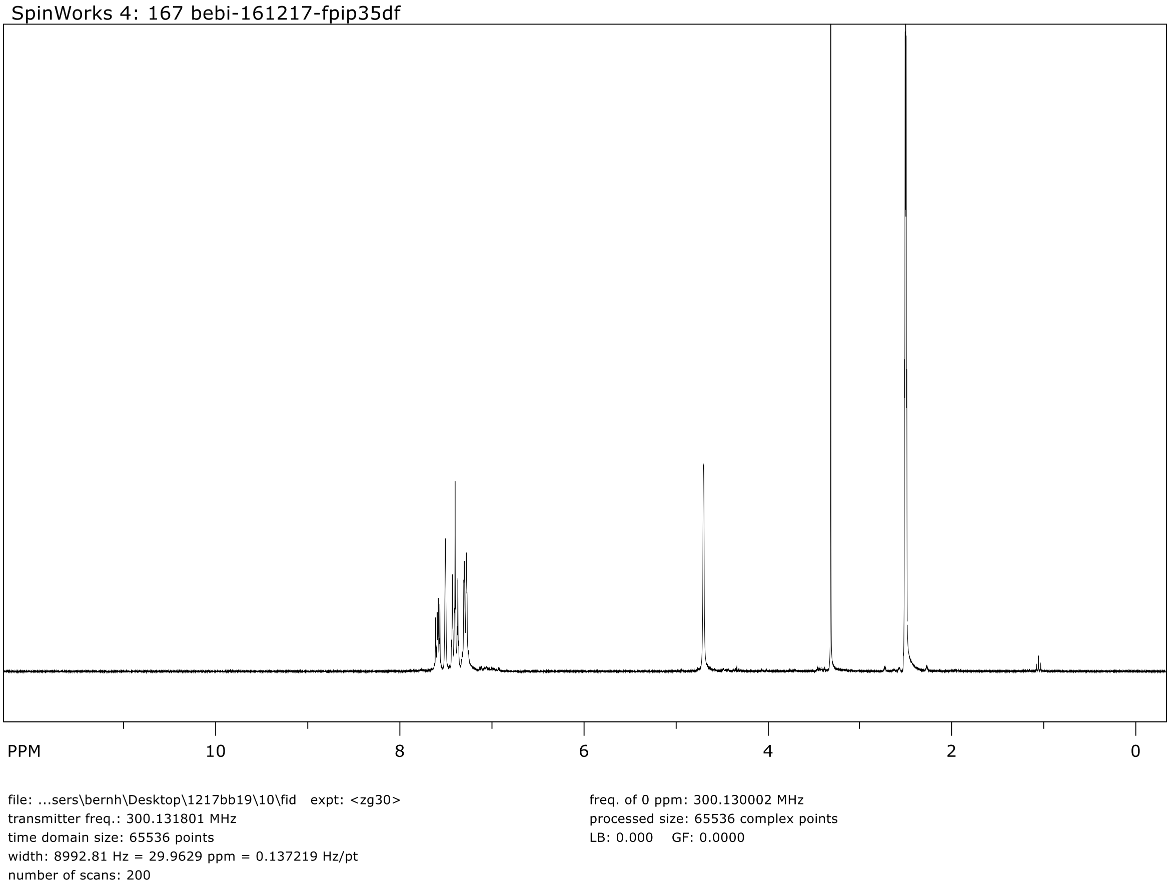

Figure S30. ^{1}H NMR spectrum of **5c**.

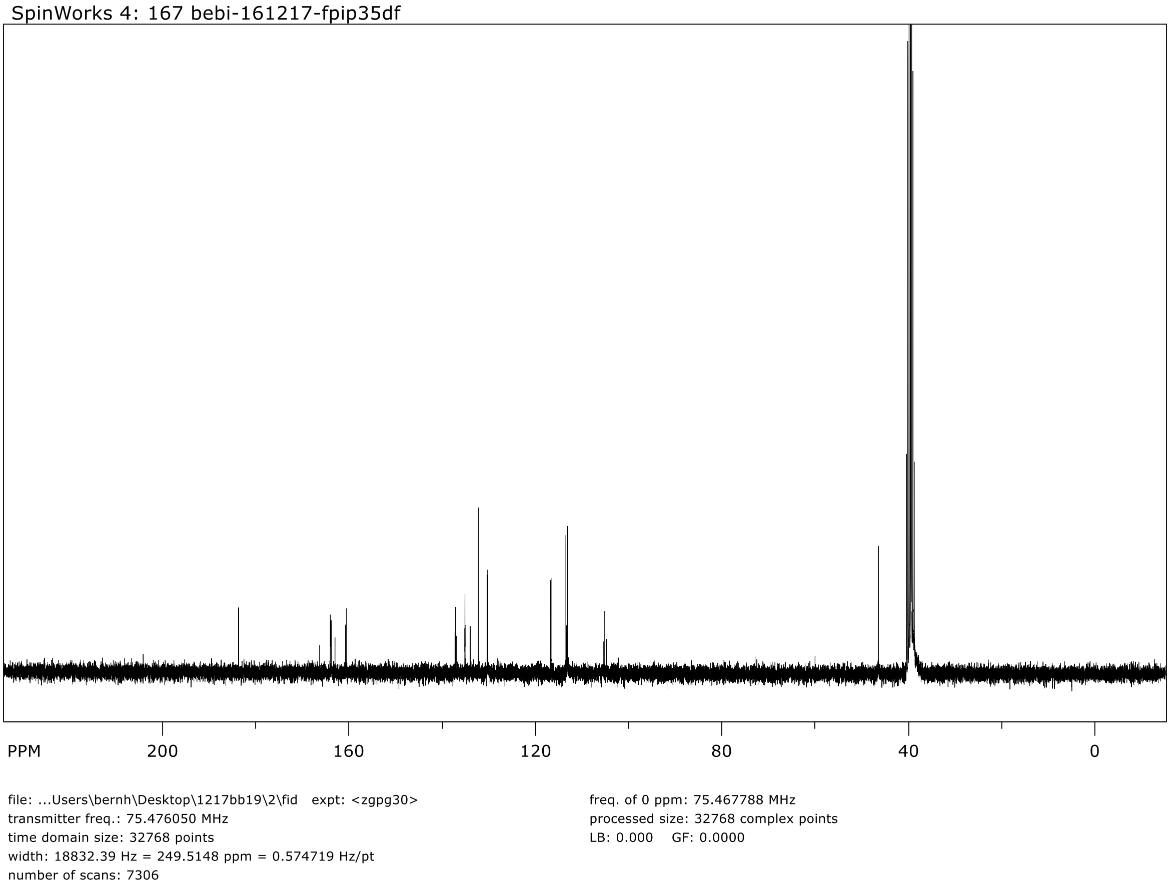

Figure S31. ^{13}C NMR spectrum of **5c**.

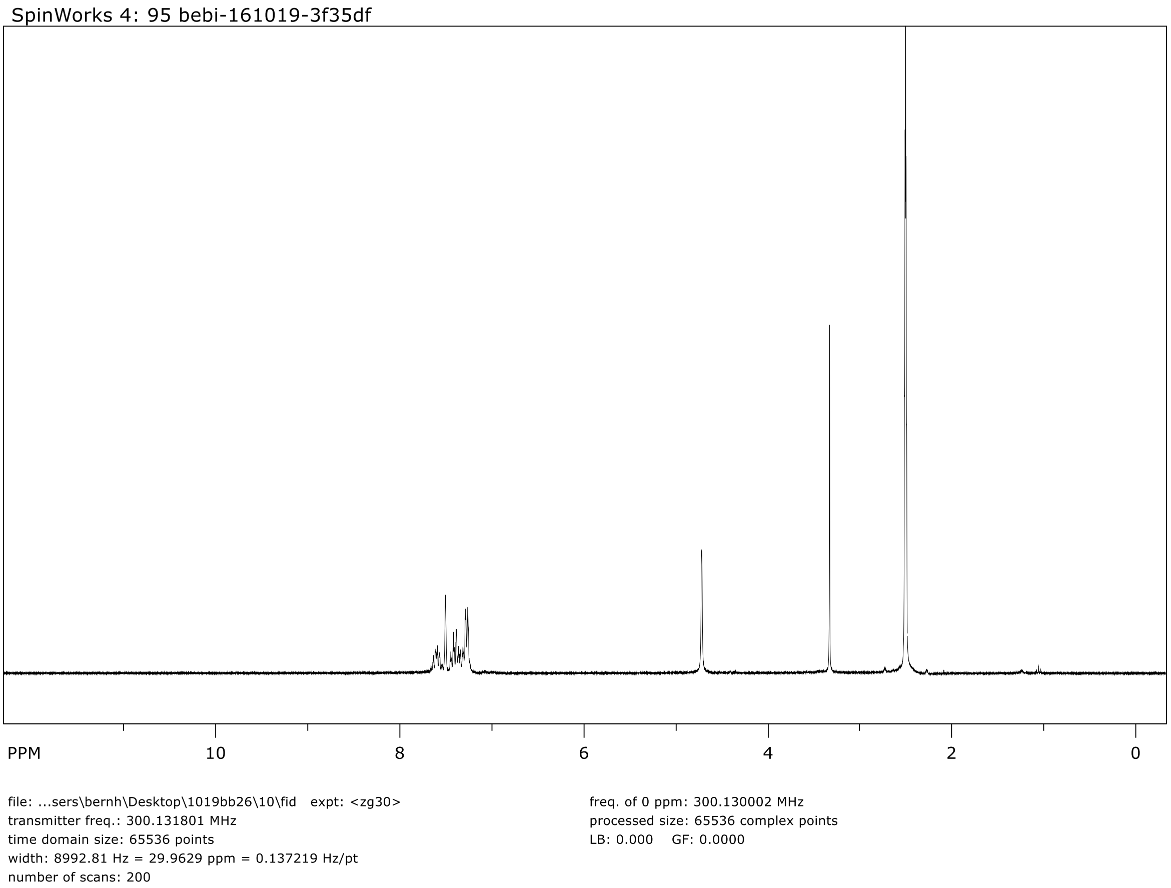

Figure S32. ^{1}H NMR spectrum of **5d**.

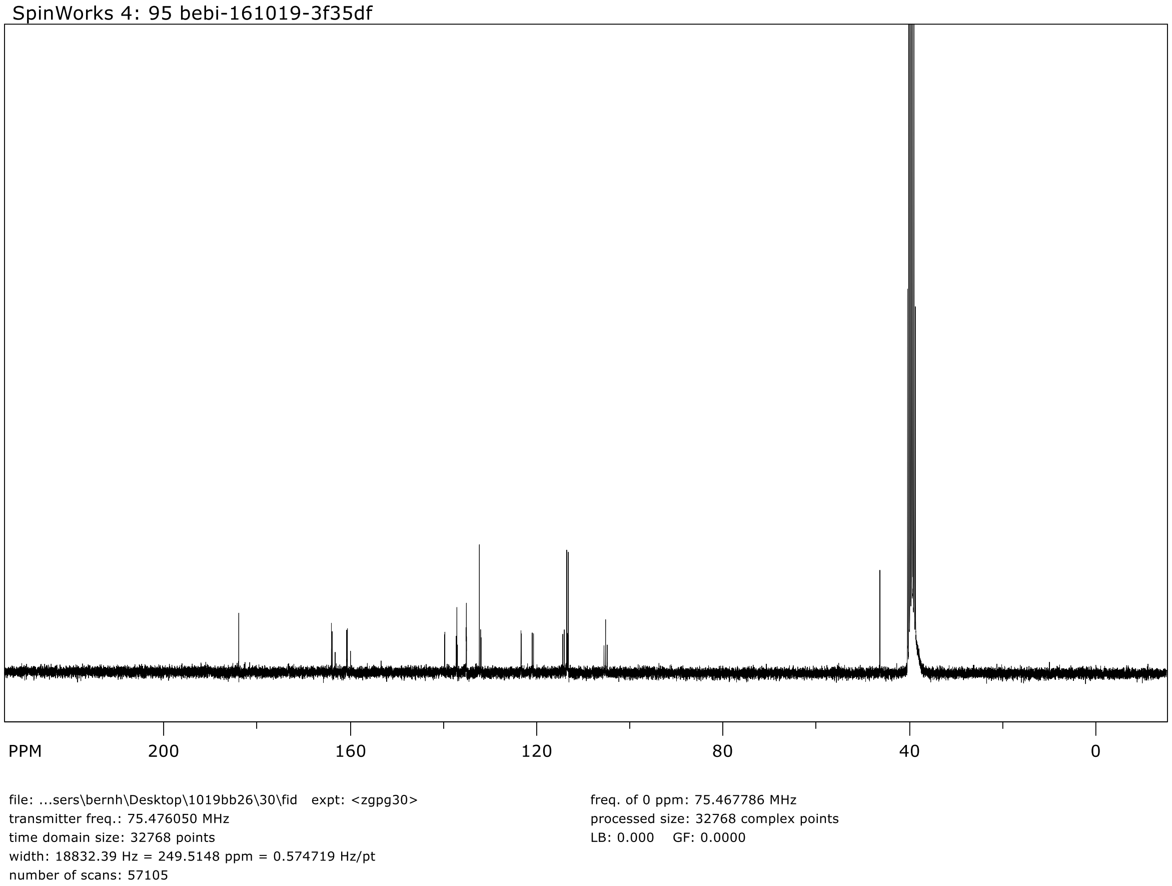

Figure S33. ^{13}C NMR spectrum of **5d**.

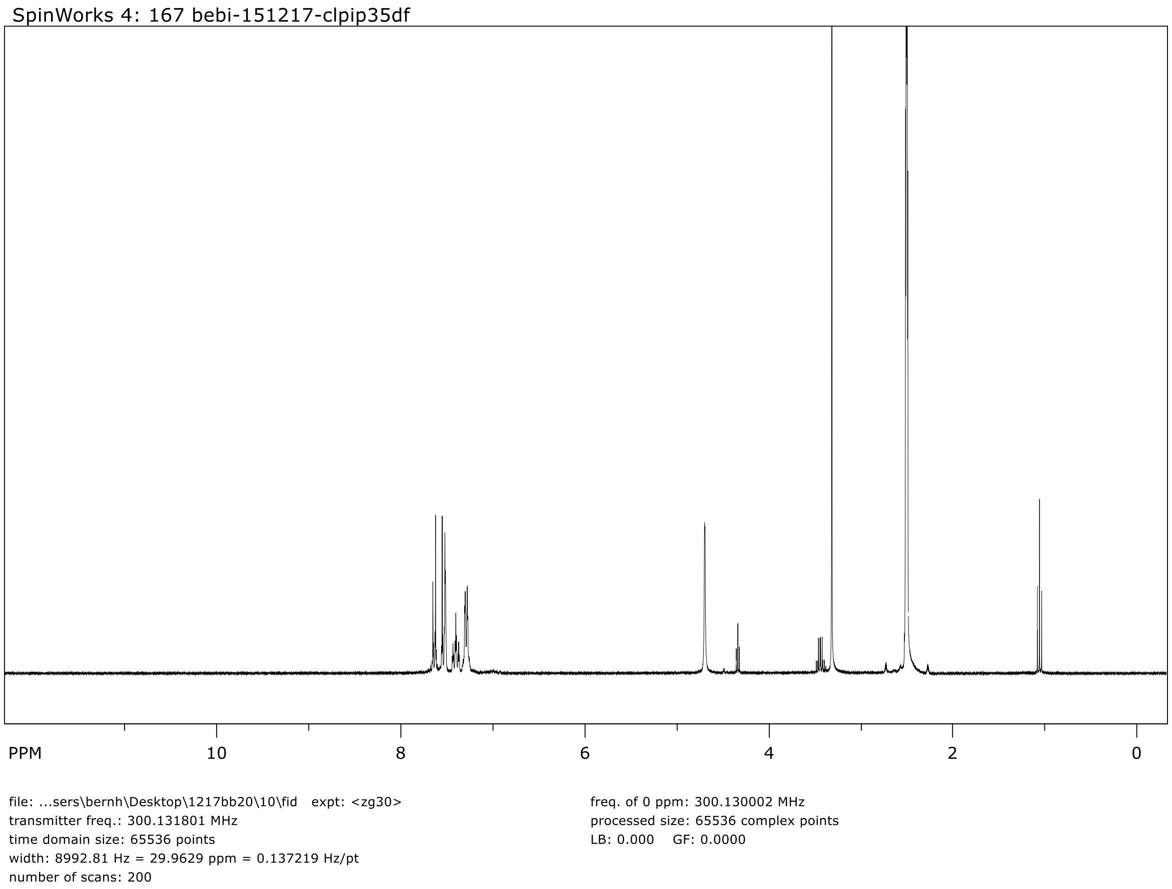

Figure S34. ^{1}H NMR spectrum of **5e**.

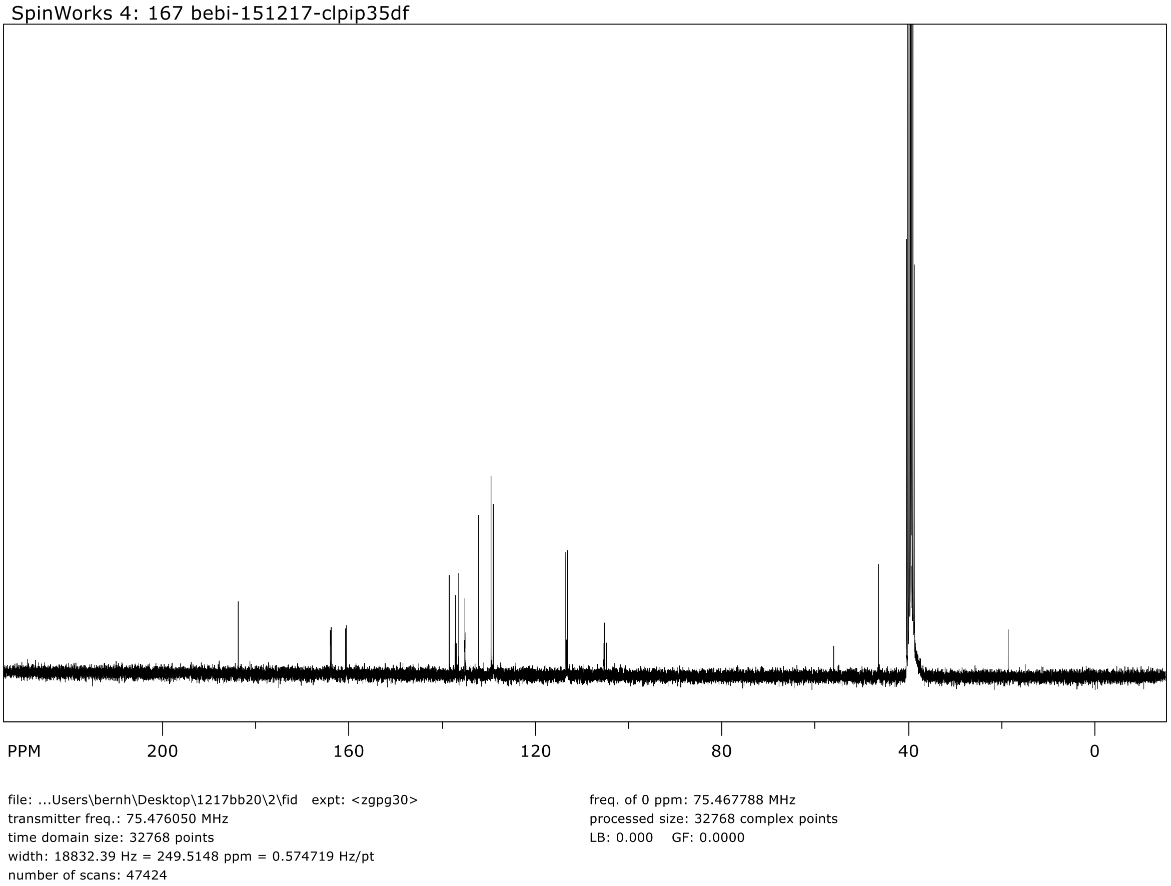

Figure S35. ^{13}C NMR spectrum of **5e**.

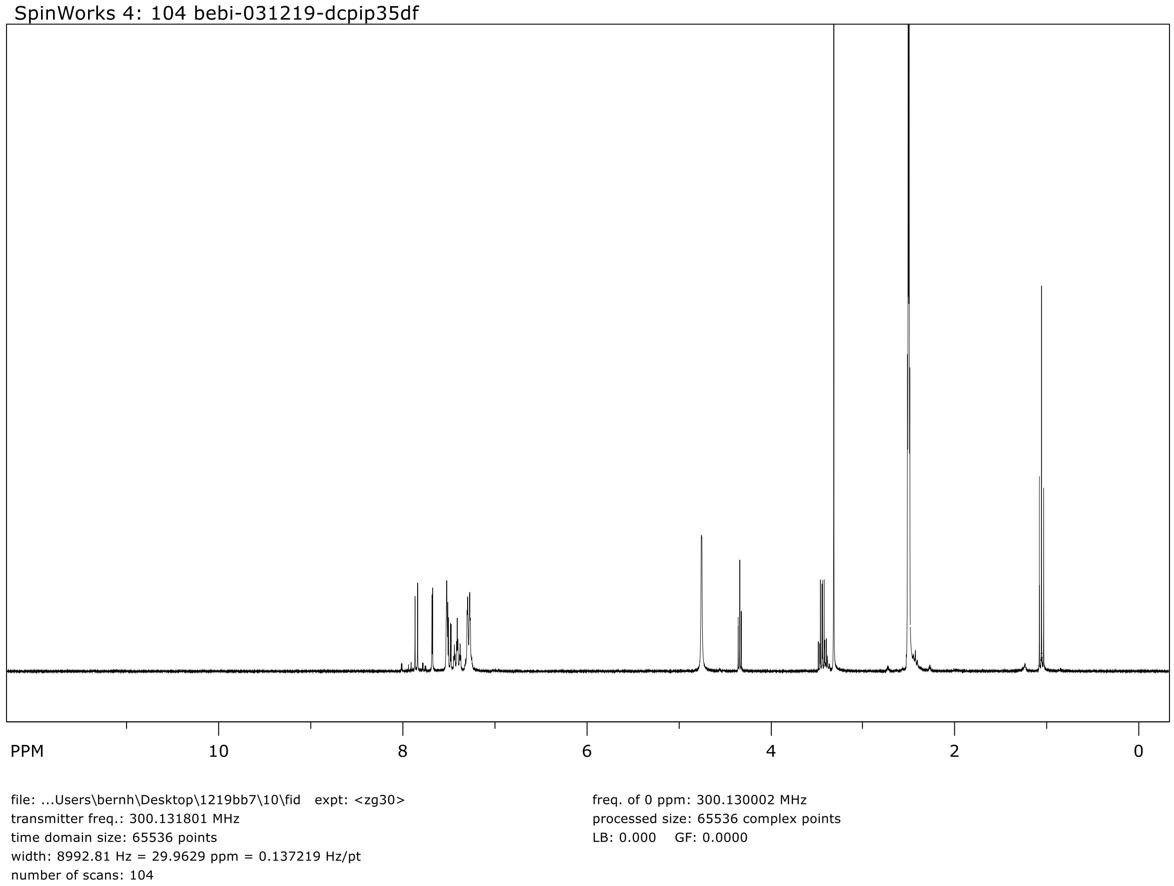

Figure S36. ^{1}H NMR spectrum of **5f**.

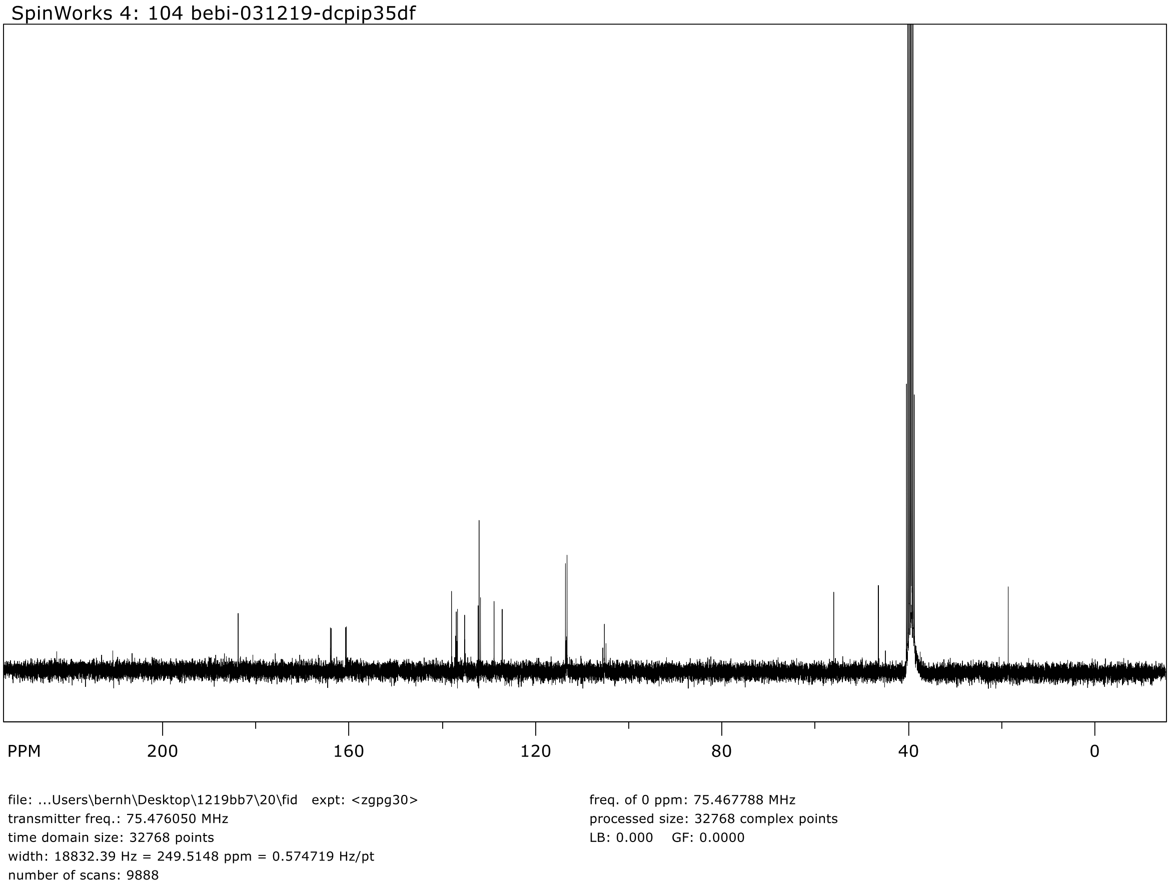

Figure S37. ^{13}C NMR spectrum of **5f**.

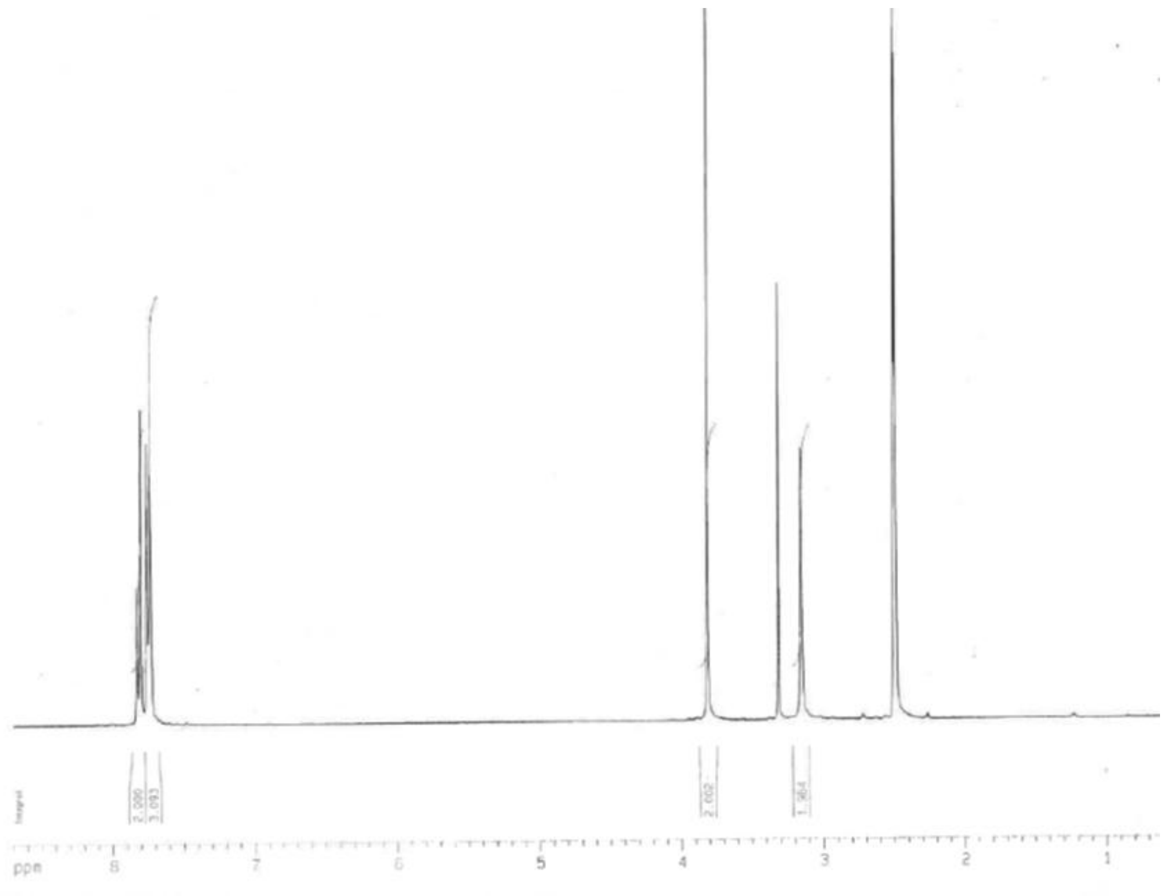

Figure 38. ^{1}H NMR spectrum of **6a**.

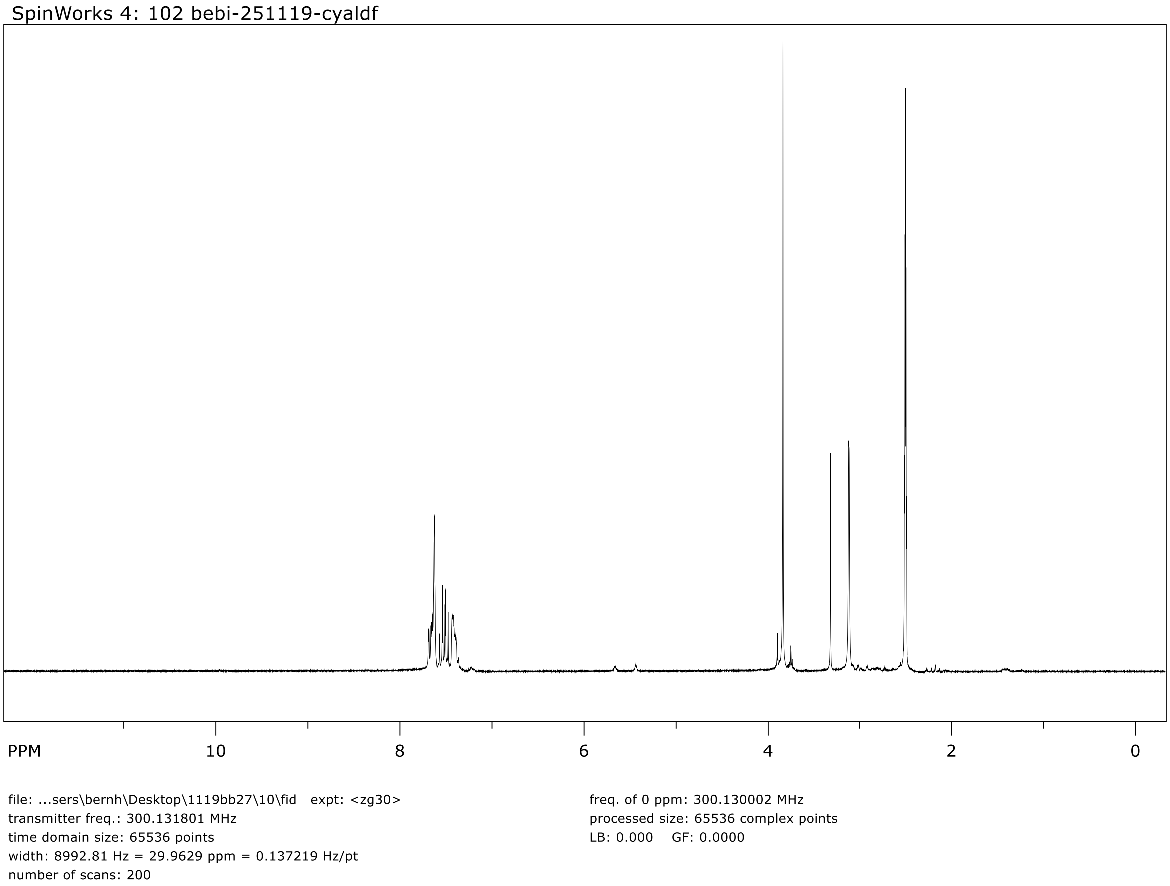

Figure S39. ^{1}H NMR spectrum of **6b**.

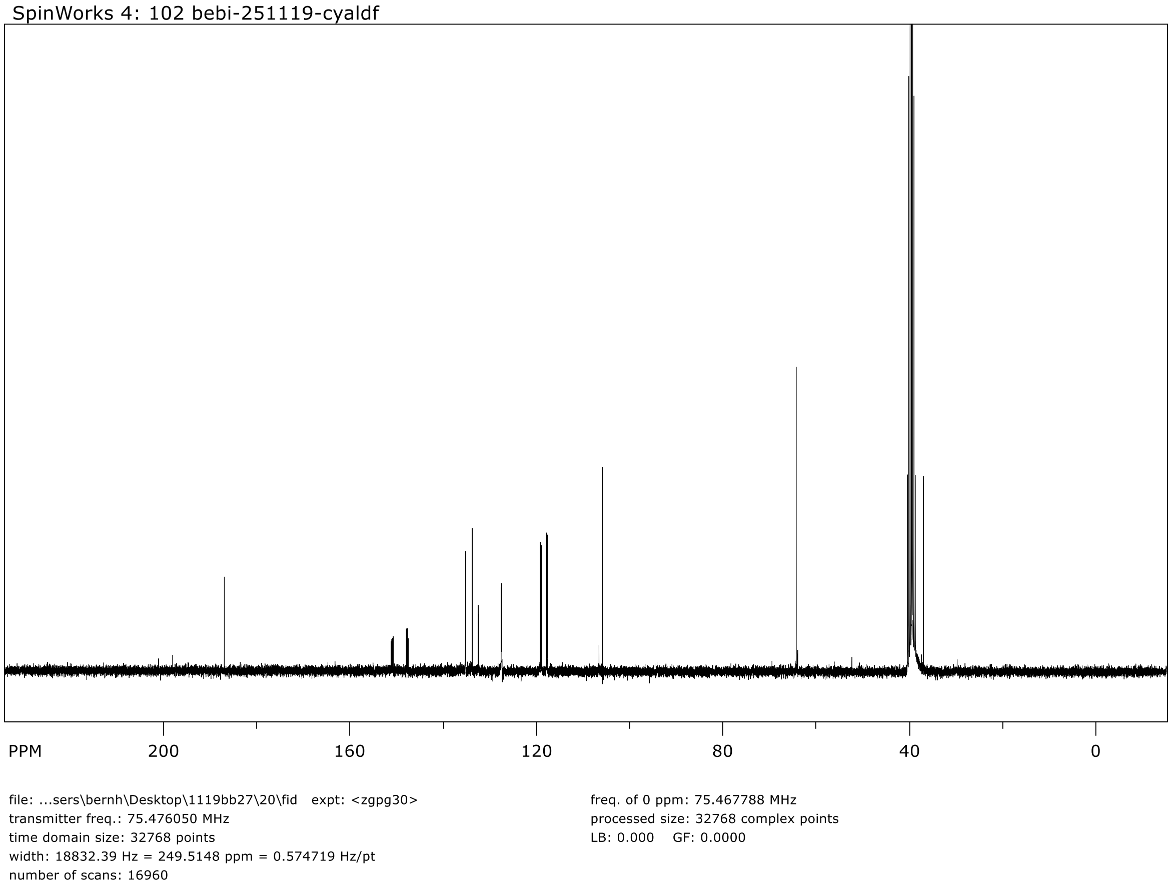

Figure S40. ^{13}C NMR spectrum of **6b**.

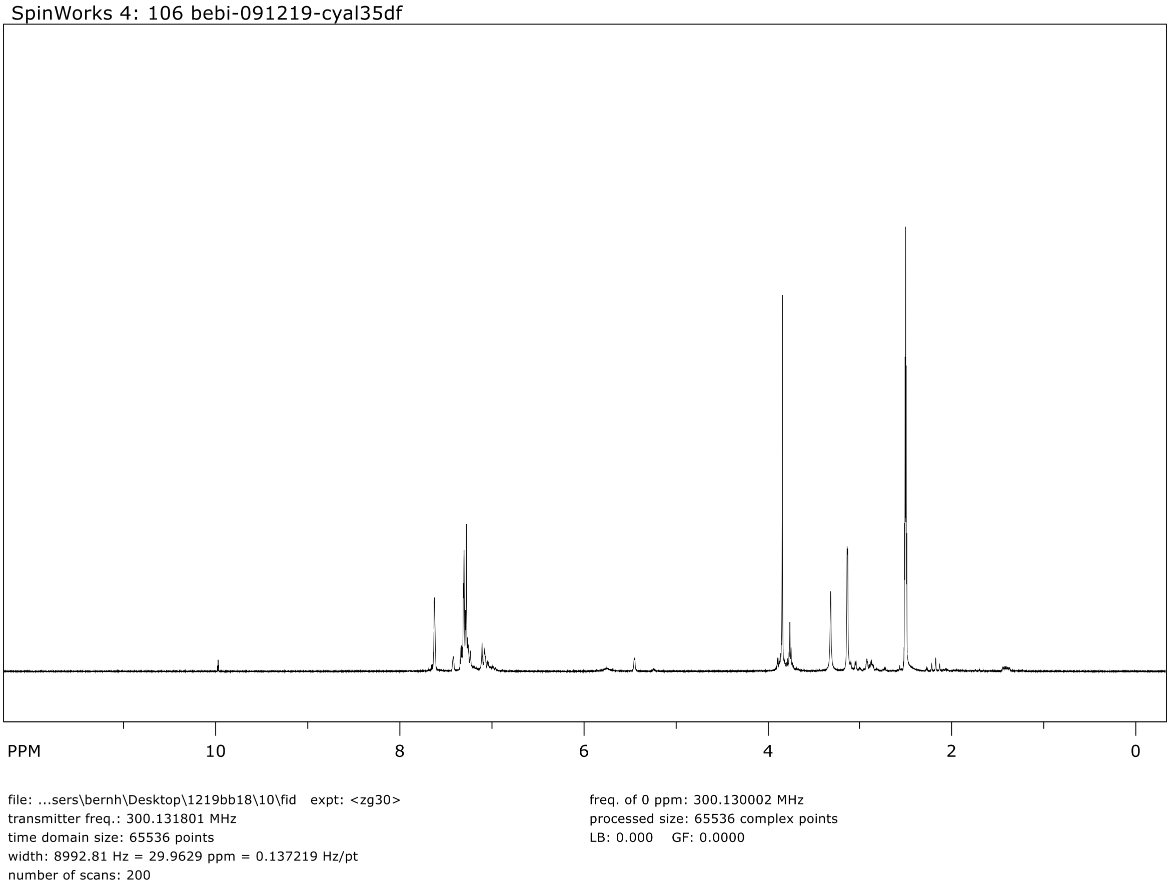

Figure S41. ^{1}H NMR spectrum of **6c**.

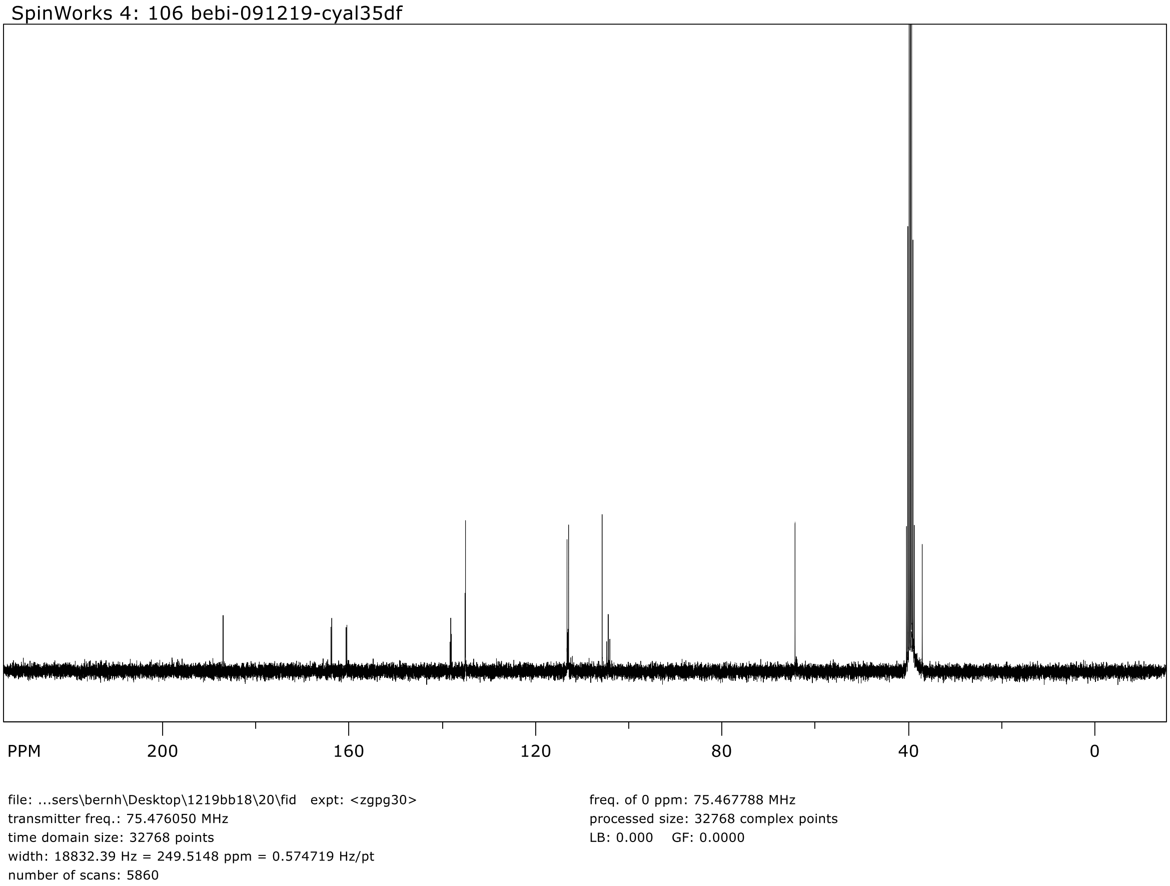

Figure S42. ^{13}C NMR spectrum of **6c**.

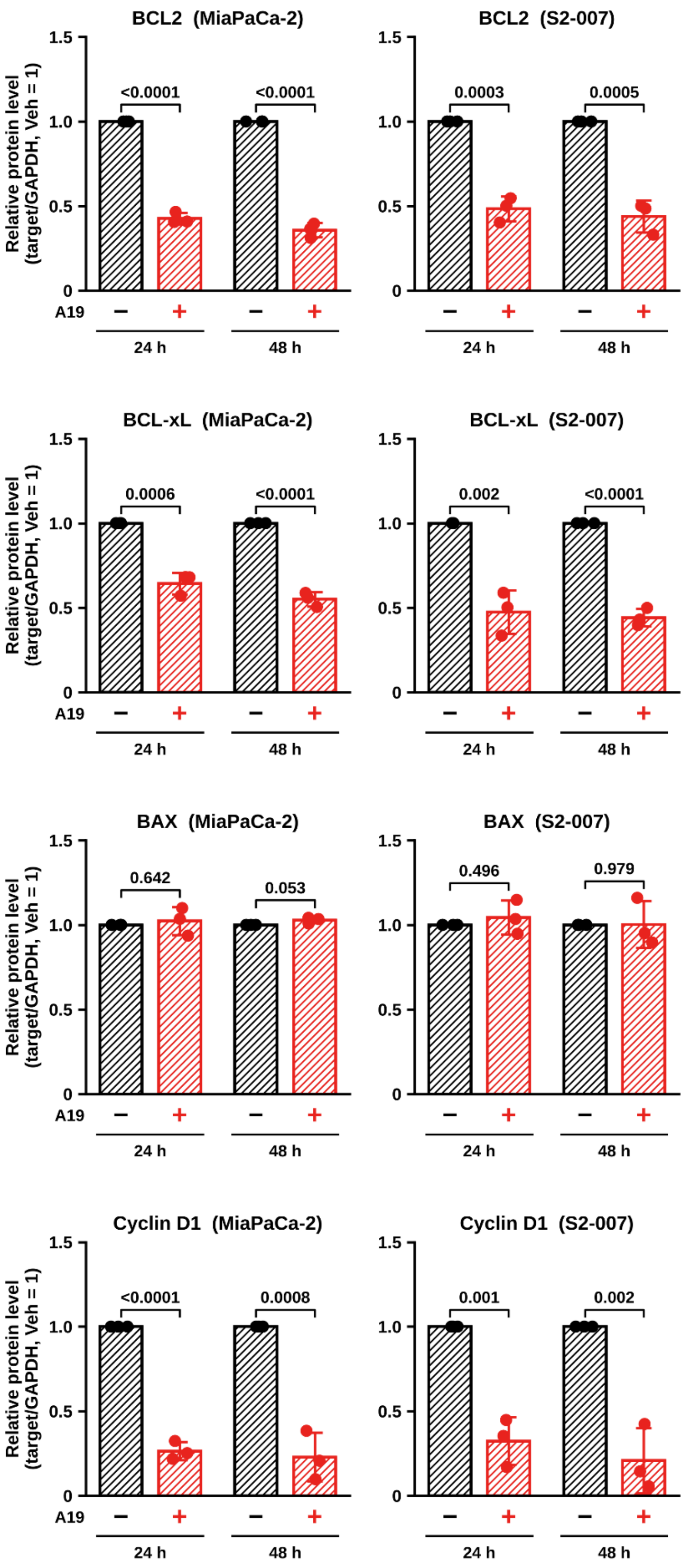

Figure S43. Densitometric quantification of the Western blots shown in Figure 4D. MiaPaCa-2 and S2-007 cells were treated with vehicle (–) or **3a** (A19) at its IC_{50} concentration (+) for 24 h or 48 h. Bcl-2, Bax, Bcl-Xl, and cyclin D1 band intensities were normalized to the corresponding GAPDH loading control and expressed relative to the time-matched vehicle control (set to 1). Data are presented as mean ± SD

of three independent experiments, with individual data points shown. p-values were determined by unpaired two-tailed Student's t-test comparing **3a**-treated cells with the time-matched Vehicle control.

Table S1. Selectivity index (SI) of **3a** (A19) for KRAS-mutant PDAC cells relative to Jurkat and THP-1 cells. SI = IC_{50} (Jurkat or THP1) / IC_{50} (PDAC cells).

	**MiaPaCa-2**	**S2-007**	**Panc-1**
**Jurkat, 48 h**	21.50±11.22	25.41±6.92	9.98±1.69
**Jurkat, 72 h**	24.57±2.50	26.46±2.80	26.46±2.80
**THP-1, 48 h**	35.50±17.77	41.95±9.58	16.48±1.35
**THP-1, 72 h**	33.93±2.65	36.54±3.04	36.54±3.04
